# Neoadjuvant Chemotherapy With Cisplatin Up‐Regulates GSDMD to Enhance Oral Squamous Cell Carcinoma Metastasis Through MMP14‐Mediated EMT Activation

**DOI:** 10.1002/advs.202501149

**Published:** 2025-04-03

**Authors:** Zixian Huang, Qiming Jiang, Qianyu Zhang, Nan Lu, Xi Rui, Rui Chen, Yan Wang, Yuepeng Wang, Xiaoding Xu, Zhiquan Huang

**Affiliations:** ^1^ Department of Oral and Maxillofacial Surgery Sun Yat‐sen Memorial Hospital, Sun Yat‐sen University Guangzhou Guangdong 510120 China; ^2^ Guangdong Provincial Key Laboratory of Malignant Tumor Epigenetics and Gene Regulation, Medical Research Center Sun Yat‐Sen Memorial Hospital, Sun Yat‐Sen University Guangzhou 510120 China; ^3^ Nanhai Translational Innovation Center of Precision Immunology Sun Yat‐Sen Memorial Hospital Foshan 528200 China; ^4^ Hospital of stomatology, the First Affiliated Hospital of Jinan University School of Stomatology, Jinan University Guangzhou 510632 China

**Keywords:** cervical lymph node metastasis, epithelial‒mesenchymal transition, gasdermin D, neoadjuvant chemotherapy, oral squamous cell carcinoma

## Abstract

Neoadjuvant chemotherapy has been widely used for the treatment of solid tumors. However, clinical observations have shown that patients with oral squamous cell carcinoma (OSCC) who are receiving neoadjuvant chemotherapy with cisplatin still face issues such as a poor lymph node response and even lymph node progression, but the underlying mechanisms remain unidentified. In this work, it is found that low‐dose cisplatin promoted oral squamous cell carcinoma migration, invasion and lymph node metastasis, and gasdermin D (GSDMD) is identified as a potential regulator. GSDMD interacted with MMP14, promoting its expression and epithelial‒mesenchymal transition (EMT) activation without activating pyroptosis. Moreover, pH‐responsive nanoparticles (NPs) for the systemic delivery of a GSDMD siRNA (siGSDMD) is developed and showed that this NP‐delivered siGSDMD can effectively inhibit OSCC tumor growth and metastasis via the efficient silencing of GSDMD expression in vivo. This findings indicate that GSDMD can be a biomarker to predict the prognosis of OSCC patients receiving neoadjuvant chemotherapy and that NP‐mediated GSDMD silencing can be a promising strategy for the treatment of patients with advanced OSCC receiving neoadjuvant chemotherapy with cisplatin.

## Graphical abstract

Oral squamous cell carcinoma cells exhibit upregulated expression of GSDMD upon exposure to low‐dose cisplatin chemotherapy, which subsequently interacts with MMP14 through its N‐terminal domain, activating the epithelial‒mesenchymal transition (EMT) process and promoting the lymph node metastasis of oral squamous cell carcinoma. Targeted delivery of siGSDMD via NPs can inhibit the cisplatin‐induced metastasis of oral squamous cell carcinoma.

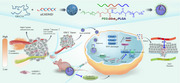

## 1 Introduction

Neoadjuvant chemotherapy has been widely used for the treatment of solid tumors.^[^ ^1^, ^2^ ^]^ Several phase II and III clinical trials have shown that the application of neoadjuvant chemotherapy is beneficial for improving the prognosis of patients with cancers, such as triple‐negative breast cancer and non‐small cell lung cancer.^[^ ^2^, ^3^, ^4^ ^]^ However, the benefit of neoadjuvant chemotherapy for oral squamous cell carcinoma (OSCC) patients remains elusive. Indeed, neoadjuvant chemotherapy can effectively reduce the tumor size, decrease the surgery risk, and facilitate the preservation of important head and neck tissues.^[^ ^4^ ^]^ Moreover, clinical observations have revealed that ≈40%–60% of OSCC patients who receive neoadjuvant chemotherapy achieve a major pathological response.^[^ ^5^, ^6^, ^7^ ^]^ However, the response of metastatic lymph nodes to neoadjuvant therapy is significantly lower than that of primary tumors, and some OSCC patients exhibit local invasive tumor microfoci around the primary tumor.^[^ ^8^, ^9^ ^]^ Additionally, some patients experience a progression of neck lymph node metastases,^[^ ^10^ ^]^ and half of the patients with lymph node metastases exhibit extracapsular invasion,^[^ ^11^ ^]^ suggesting a potentially unfavorable lymph node status in OSCC patients receiving neoadjuvant therapy. This clinical phenomenon contradicts the original intention of neoadjuvant chemotherapy to create better surgical conditions by reducing the tumor burden and warrants further investigation into its potential regulatory mechanisms.

Postchemotherapy metastasis and tumor recurrence are caused mainly by the progression of resistant and dormant lesions.^[^ ^12^ ^]^ However, emerging evidence has shown that chemotherapy itself can alter tumor biology and induce tumor metastasis.^[^ ^13^ ^]^ Chemotherapy can promote the metastasis of breast and liver cancers through various mechanisms, such as alterations in the tumor microenvironment, activation of the CCL20‒CCR6 pathway in tumor‐associated macrophages (TAMs), and an enhancement of VEGF signaling pathway activity in tumor cells.^[^ ^14^, ^15^, ^16^ ^]^ In OSCC, tumor cells with increased migration and invasion abilities may significantly increase the risk of tumor metastasis through the rich distribution of blood and lymphatic vessels in the head and neck region.^[^ ^17^ ^]^ Recent studies have shown that even in the early stage (cT1‐2), 20–40% of OSCC patients have already developed neck lymph node metastasis.^[^ ^18^, ^19^ ^]^ More importantly, these metastatic OSCC cells can adhere to surrounding tissue structures and blood vessels in the neck, significantly increasing the difficulty and risk of subsequent therapy, such as surgery.^[^ ^20^, ^21^ ^]^ Therefore, much more effort is needed to elucidate the important role of neoadjuvant chemotherapy in regulating the lymph node metastasis of OSCC. Identifying key biomarkers and elucidating their regulatory mechanisms are beneficial for predicting and treating the neck lymph node metastasis of OSCC.

In this work, we identified gasdermin D (GSDMD) as the key factor regulating lymph node metastasis in OSCC patients who received neoadjuvant chemotherapy. A mechanistic study revealed that GSDMD was involved in regulating OSCC metastasis through a novel mechanism involving interactions with MMP14 to upregulate MMP14 expression, thereby mediating the tumor EMT process and inducing OSCC metastasis without activating the classical pyroptosis process. Considering this mechanism and the ability of small interfering RNAs (siRNAs) to silence the expression of any target gene,^[^ ^22^, ^23^, ^24^ ^]^ we further developed pH‐responsive nanoparticles (NPs) for the systemic codelivery of a cisplatin prodrug and GSDMD siRNA (siGSDMD) and showed that this codelivery system could effectively inhibit OSCC tumor growth and metastasis with reduced toxicity and side effects (scheme).

## 2 Results

### 2.1 Neck Lymph Node Metastasis Occurred as an Adverse Event in OSCC Patients Receiving Cisplatin‐Based Neoadjuvant Chemotherapy

We collected 80 OSCC patients who received neoadjuvant chemotherapy and reviewed their responses to neoadjuvant chemotherapy to validate the possible adverse events of neoadjuvant chemotherapy in OSCC patients. Although a favorable clinical outcome was observed, with a reduced primary lesion of OSCC found in more than 90% of patients, the response of lymph node lesions to neoadjuvant chemotherapy was unsatisfactory. Lymph node reductions on MRI were found in only half of the patients, with lymph node progression observed in 9 patients. Notably, the emergence of new cervical lymph node metastases was observed in 3 patients. Among them, 2 patients presented extensive new ipsilateral lymph node metastases on MRI, whereas the other patient presented new contralateral lymph node metastases (**Table** **1**, Figure S1 and Table S1, Supporting Information). These findings suggest that cervical lymph node metastasis may occur as an adverse event following neoadjuvant chemotherapy in patients with OSCC. These results raise the possibility that neoadjuvant chemotherapy could act as a factor inducing lymph node metastasis in oral squamous cell carcinoma.

**Table 1:** Analysis of the efficacy of neoadjuvant chemotherapy for OSCC.

OSCC Lesion	Cervical Lymphatic Lesion		
	Remission	Sustained	Progression
Remission	37	29	9
Sustained	2	2	
Progression		1	

**Figure 1:**
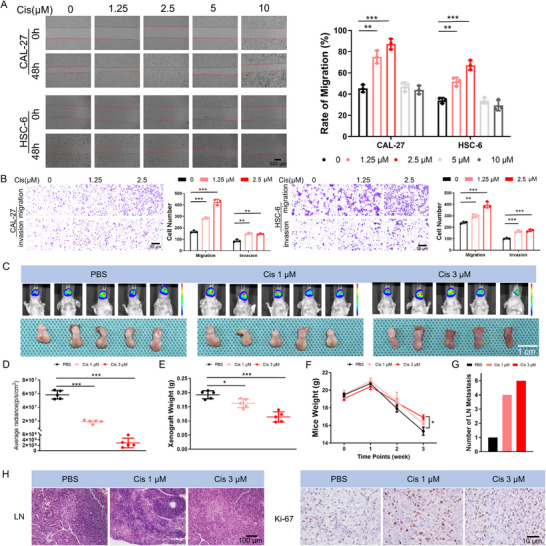
Low‐concentration cisplatin promotes the migration, invasion, and lymph node metastasis of oral squamous cell carcinoma (OSCC). A) Scratch wound healing assays revealed the significantly enhanced migration capabilities of CAL‐27 and HSC‐6 cells treated with 1.25 and 2.5 µM cisplatin, while higher concentrations (5 and 10 µM) did not significantly increase migration. B) The results of Transwell assays revealed increased the migration and invasion of CAL‐27 and HSC‐6 cells treated with 1.25 and 2.5 µM cisplatin. C) In vivo luminescence imaging and tumor imaging after cisplatin chemotherapy in a mouse tongue orthotopic xenograft model. D) The luminescence intensity of orthotopic xenografts in mice treated with cisplatin was significantly decreased compared with that in the control group. E) Tumor weights of orthotopic xenografts from mice treated with cisplatin were lower than those from control mice. F) Mice treated with 3 µM cisplatin exhibited a lower degree of weight loss than the other two groups. G) Increased cervical lymph node metastasis was observed in the mice treated with cisplatin chemotherapy compared with the control group. H) HE staining showed increased cervical lymph node metastasis in mice treated with cisplatin, and immunohistochemistry revealed increased expression levels of Ki‐67 in their xenografts.

Based on these results, we further validated the phenomenon of chemotherapy‐induced OSCC metastasis both in vitro and in vivo. We compared the metastatic abilities of OSCC cells (CAL‐27 and HSC‐6) treated with various concentrations of chemotherapeutic drugs (cisplatin, docetaxel, and 5‐fluorouracil). The appropriate drug concentrations were selected based on the sensitivity curves of OSCC cells (Figure S2A, Supporting Information). The wound healing assay results revealed that treatment with 1.25 and 2.5 µM cisplatin significantly increased the migration of OSCC cells, whereas after treatment with higher concentrations (5 and 10 µM), the migration of OSCC cells did not significantly increase compared with that of the control group (**Figure** **1**A). Transwell assay results further confirmed that treatment with low concentrations of cisplatin could enhance the migration and invasion of OSCC cells (Figure 1B). Moreover, docetaxel and 5‐fluorouracil treatment did not significantly promote the migration or invasion of OSCC cells (Figure S2B–E). These results indicate that cisplatin plays an important role in regulating OSCC metastasis. We established a nude mouse tongue orthotopic xenograft model and treated the mice with different concentrations of cisplatin to further test this hypothesis. Compared with the control group, both the 1 and 3 µM cisplatin chemotherapy groups presented significantly reduced tumor volumes (Figure 1C–E), with less weight loss in the mice (Figure 1F) but an increased incidence of neck lymph node metastasis and increased expression of Ki‐67 (Figure 1G,H). These results strongly demonstrate that cisplatin‐based chemotherapy not only exerts an anticancer effect but also enhances OSCC metastasis as an adverse event.

### 2.2 GSDMD Regulates OSCC Metastasis after Neoadjuvant Chemotherapy

**Figure 2:**
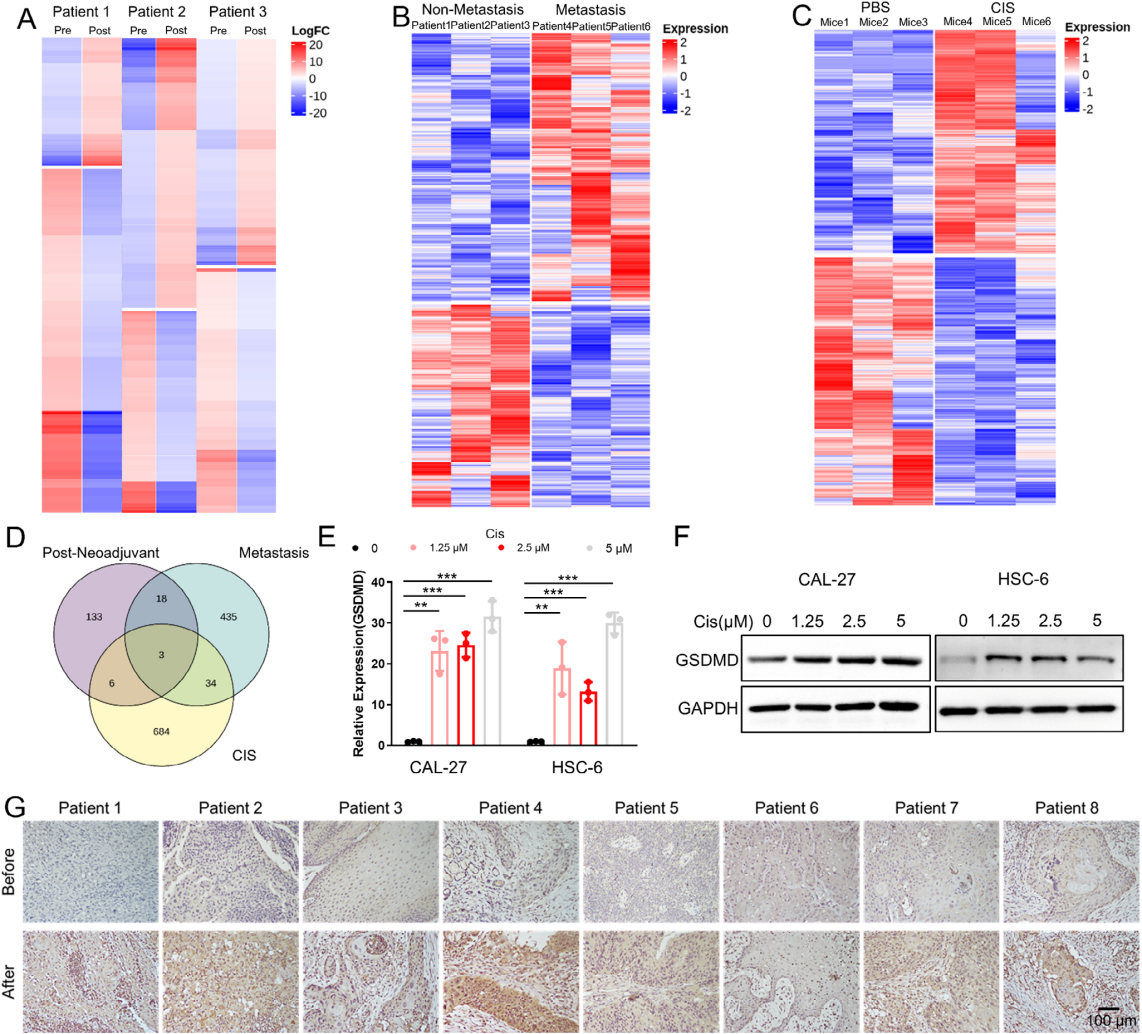
High‐throughput sequencing reveals increased expression of GSDMD in oral squamous cell carcinoma (OSCC) following neoadjuvant chemotherapy. A) High‐throughput sequencing of tumor tissues from patients with OSCC before and after neoadjuvant chemotherapy. B) High‐throughput sequencing of tumor tissues from OSCC patients with and without cervical lymph node metastasis. C) High‐throughput sequencing of tumor tissues from mice in the chemotherapy group with lymph node metastasis and the control group without metastasis. D) An intersection analysis of the three sequencing groups identified potential regulatory targets, namely, GSDMD, CCND1, and BATF2. E) Upregulation of GSDMD transcript levels in CAL‐27 and HSC‐6 cells following cisplatin treatment. F) Upregulation of GSDMD protein levels in CAL‐27 and HSC‐6 cells following cisplatin treatment. G) GSDMD expression in the tumor tissues of patients before and after neoadjuvant chemotherapy.

We explored the specific regulatory mechanism of neoadjuvant chemotherapy‐induced neck lymph node metastasis of OSCC by collecting primary tumor tissues from OSCC patients before and after neoadjuvant chemotherapy (**Figure** **2**A), primary tumor tissues from OSCC patients with and without neck lymph node metastasis (Figure 2B), and xenograft tissues from mice in the chemotherapy group with lymph node metastasis and the control group without metastasis in the abovementioned mouse tongue orthotopic xenograft model (Figure 2C) for high‐throughput sequencing. The intersection analysis revealed that three factors, GSDMD, CCND1, and BATF2 (Figure 2D), were upregulated in postchemotherapy OSCC tumor tissues and highly expressed in tumors from patients with neck lymph node metastasis. These findings indicate that these three factors are abnormally overexpressed in OSCC after neoadjuvant chemotherapy and may participate in the regulation of lymph node metastasis. Furthermore, we confirmed that GSDMD mRNA and protein expression levels were significantly increased in OSCC cells after cisplatin treatment (Figure 2E,F). We further examined and verified a significant increase in GSDMD expression after neoadjuvant chemotherapy in the tumor tissues of OSCC patients (Figure 2G, **Table** **2**) and observed a significant association between high GSDMD expression and lymph node metastasis in OSCC patients (**Table** **3**). Additionally, we verified that although CCND1 (Figure S3A,B, Supporting Information) and BATF2 (Figure S4A,B, Supporting Information) expression levels were also increased after cisplatin treatment in OSCC cell lines, knockdown of CCND1 (Figure S3C–F, Supporting Information) or BATF2 (Figure S4C–F, Supporting Information) did not result in a significant change in the migration or invasion of OSCC cells. Therefore, we conclude that GSDMD expression is upregulated in OSCC after cisplatin‐based chemotherapy and thus promotes OSCC metastasis.

**Table 2:** Alterations in GSDMD expression in the tumor tissues of patients before and after neoadjuvant chemotherapy.

	GSDMD Expression		*p*
	High Expression [%]	Low Expression [%]	
Pre‐Neoadjuvant Chemotherapy	20 (50.00)	20 (50.00)	0.001
Post‐Neoadjuvant Chemotherapy	35 (87.50)	5 (12.50)	

**Table 3:** Association of GSDMD expression with the clinical features of OSCC patients.

Clinical Features	GSDMD Expression		*p*
	High Expression	Low Expression	
Age, years	57.75±11.90	59.40±10.88	0.379
≤40	6 (75.00)	2 (25.00)	0.743
41‐50	10 (66.67)	5 (33.33)	
51–60	25 (58.14)	18 (41.86)	
>60	31 (57.41)	23 (42.59)	
Sex			
Male	52 (59.09)	36 (40.91)	0.736
Female	20 (62.50)	12 (37.50)	
Differentiation			
Well	18 (54.55)	15 (45.45)	0.187
Moderate	26 (54.17)	22 (45.83)	
Poor	28 (71.79)	11 (28.21)	
Lymph Node Metastasis			
No	29 (42.03)	40 (57.97)	<0.001
Yes	43 (84.31)	8 (15.69)	
Pathological T stage			
1	12 (54.55)	10 (45.45)	0.054
2	14 (43.75)	18 (56.25)	
3	30 (65.22)	16 (34.78)	
4	16 (80.00)	4 (20.00)	

**Figure 3:**
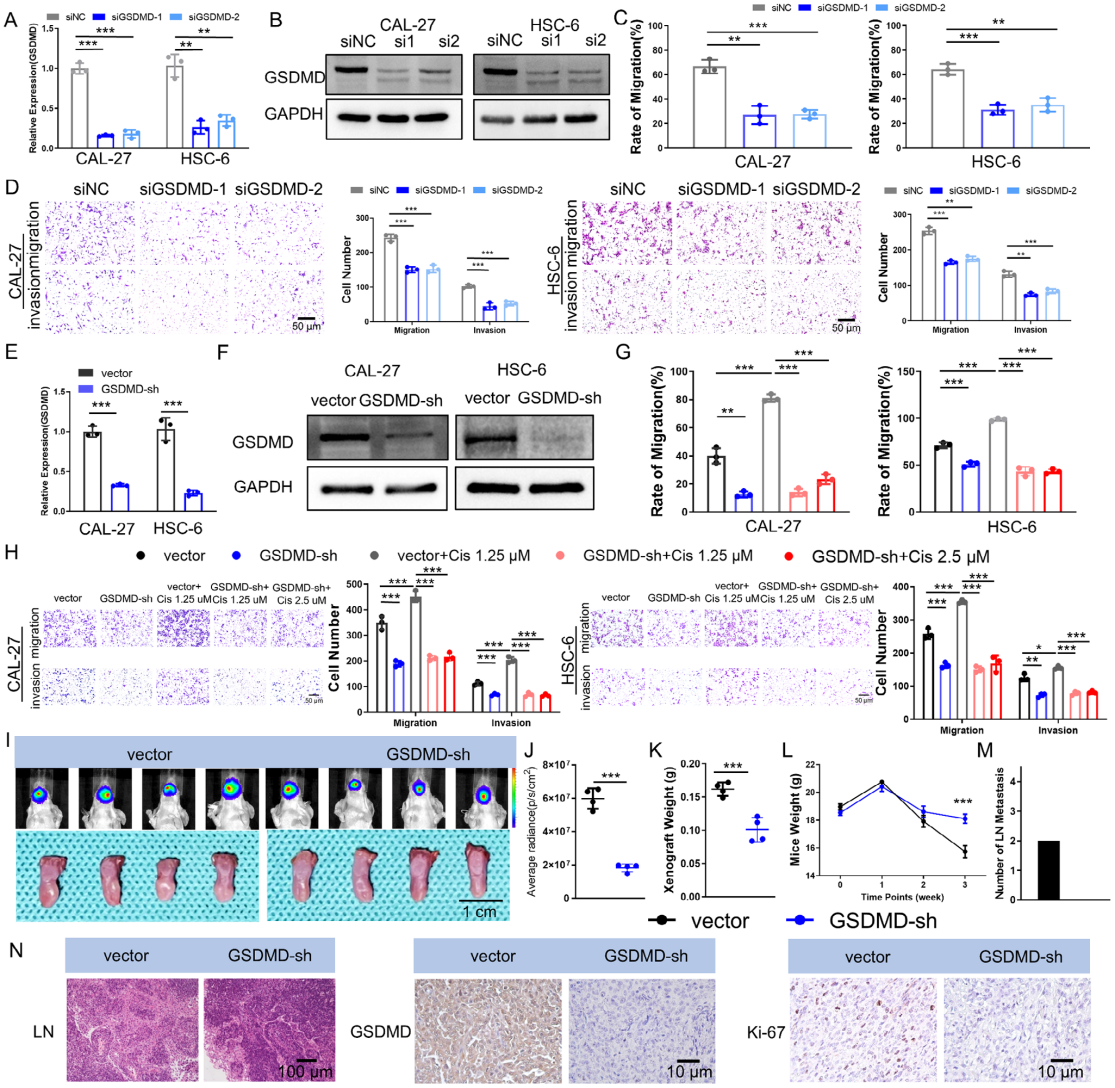
Knockdown of GSDMD reduces the migration, invasion, and lymph node metastasis of OSCC. A) Downregulation of GSDMD transcript levels in CAL‐27 and HSC‐6 cells following GSDMD knockdown. B) Downregulation of GSDMD protein levels in CAL‐27 and HSC‐6 cells following GSDMD knockdown. C) Scratch wound healing assays showed a significant decrease in the migration ability of CAL‐27 and HSC‐6 cells following GSDMD knockdown. D) Transwell assays revealed a significant decrease in the migration and invasion abilities of CAL‐27 and HSC‐6 cells following GSDMD knockdown. E) Downregulation of GSDMD transcript levels in GSDMD‐sh OSCC cells. F) Downregulation of GSDMD protein levels in GSDMD‐sh OSCC cells. G) Scratch wound healing assays revealed that the stable expression of shGSDMD significantly reduced the migration of CAL‐27 and HSC‐6 cells and inhibited the ability of cisplatin to promote the migration of oral squamous cell carcinoma cells. H) Transwell assays showed that stable expression of shGSDMD significantly reduced the migration and invasion of CAL‐27 and HSC‐6 cells and inhibited the ability of cisplatin to promote the migration and invasion of oral squamous cell carcinoma cells. I) In vivo luminescence imaging and tumor image of GSDMD‐sh tongue orthotopic xenograft models. J) A low luminescence intensity was observed in tongue orthotopic xenografts from the GSDMD‐sh group. K) Tumor weights of tongue orthotopic xenografts from the GSDMD‐sh group were lower than those from the control group. L) Mice in the GSDMD‐sh group exhibited a lower degree of weight loss than did those in the control group. M) No lymph node metastasis was observed in mice in the GSDMD‐sh group, whereas lymph node metastasis was observed in two mice in the control group. N) HE staining showed no lymph node metastasis in the mice in the GSDMD‐sh group, and immunohistochemistry revealed lower expression levels of GSDMD and Ki‐67 in the tumors of these mice.

We first knocked down GSDMD in OSCC cell lines using RNAi technology (**Figure** **3**A,B) and validated its regulatory effect on the biological behavior of OSCC cells to further clarify the regulatory role of GSDMD in OSCC metastasis. After GSDMD was knocked down in CAL‐27 and HSC‐6 cells, both wound healing (Figure 3C; Figure S5A, Supporting Information) and Transwell (Figure 3D) assays revealed significant decreases in the migration and invasion abilities of OSCC cells. Additionally, GSDMD knockdown resulted in simultaneous decreases in the colony formation and proliferation rates of OSCC cells (Figure S6A,B, Supporting Information), while the sensitivity of these cells to cisplatin did not change significantly (Figure S7, Supporting Information). We subsequently constructed cell lines with stable knockdown or overexpression of GSDMD: GSDMD‐sh (Figure 3E,F), or GSDMD‐ov (Figure S8A,B, Supporting Information). In the GSDMD‐sh cell line, we confirmed that it also inhibited the migration and invasion of OSCC cells (Figure 3G,H; Figure S5B, Supporting Information) and their proliferation (Figure S6C,D, Supporting Information). Moreover, when GSDMD‐sh cells were stimulated with low concentrations of cisplatin, the migration and invasion abilities of OSCC cells did not increase (Figure 3G,H; Figure S5B, Supporting Information), indicating that inhibiting GSDMD expression in OSCC cells effectively blocked the increases in cisplatin‐induced migration and invasion. Conversely, in the GSDMD‐ov cell line, the migration and invasion abilities of OSCC cells were significantly enhanced (Figure S8C,D, Supporting Information), as was their proliferation (Figure S8E,F, Supporting Information).

We established a tongue orthotopic xenograft model in nude mice using the CAL‐27‐GSDMD‐sh and GSDMD‐ov cell lines. The results showed that the tumors in the GSDMD‐sh group were smaller in size (Figure 3I,J) and weight (Figure 3K) than those in the control group, with less weight loss in the GSDMD‐sh group than in the control group (Figure 3L), and no lymph node metastasis occurred (2/4 of the mice in the control group had tumor metastasis) (Figure 3M,N). The immunohistochemical results showed that GSDMD expression was effectively inhibited in the GSDMD‐sh group, with a decreased Ki‐67 positivity rate (Figure 3N). In contrast, all the mice in the GSDMD‐ov group developed tumor metastasis in the lymph nodes (2/4 of the mice in the control group had tumor metastasis), with larger tumors, greater impacts on mouse weight, and higher GSDMD and Ki‐67 expression (Figure S8G–L, Supporting Information).

### 2.3 GSDMD Regulates OSCC Metastasis through the MMP14–EMT Pathway

**Figure 4:**
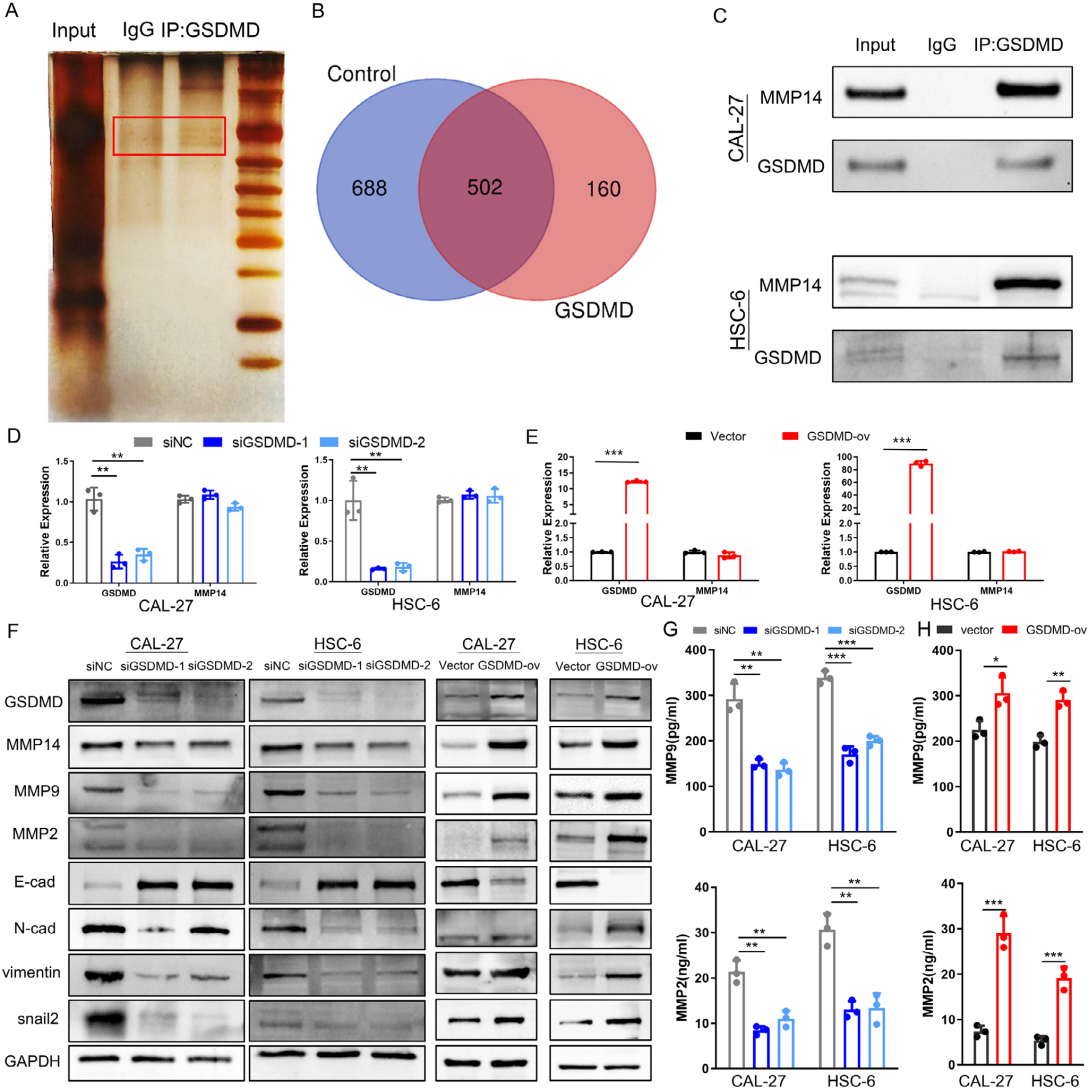
Mass spectrometry analysis and validation of GSDMD‐interacting proteins. A) Coimmunoprecipitation (Co‐IP) identified proteins that bind to GSDMD. B) An intersection analysis of protein mass spectrometry data was performed to detect differentially expressed proteins that interact with GSDMD. C) Co‐IP confirmed the interaction between GSDMD and MMP14. D) Knockdown of GSDMD did not significantly alter the mRNA level of MMP14. E) Overexpression of GSDMD did not significantly alter the mRNA level of MMP14. F) Knockdown of GSDMD resulted in the synchronized downregulation of MMP14 protein expression in OSCC cells, along with the downstream downregulation of MMP9, MMP2, N‐cadherin, vimentin, and snail2 and upregulation of E‐cadherin, suggesting the inhibition of the EMT process. Overexpression of GSDMD resulted in the upregulation of MMP14 protein expression in OSCC cells, along with the downstream upregulation of MMP9, MMP2, N‐cadherin, vimentin, and snail2 and downregulation of E‐cadherin, suggesting the activation of the EMT process. G) Knockdown of GSDMD led to reduced secretion of MMP9 and MMP2 from OSCC cells. H) Overexpression of GSDMD led to increased secretion of MMP9 and MMP2 from OSCC cells.

We explored the molecular mechanism by which GSDMD regulates OSCC metastasis using coimmunoprecipitation and mass spectrometry to identify the key proteins that interact with GSDMD (**Figure** **4**A,B; Figure S9, Supporting Information). Among them, we focused on matrix metalloproteinase 14 (MMP14), an important protein involved in the EMT process in tumor cells. We subsequently verified the interaction between GSDMD and MMP14 through coimmunoprecipitation and western blotting (Figure 4C). Real‐time quantitative polymerase chain reaction (RT‒qPCR) revealed that the level of the MMP14 transcript did not change noticeably after downregulating or upregulating GSDMD expression in OSCC cells (Figure 4D,E). However, at the protein level, MMP14 expression decreased significantly after GSDMD silencing but increased with GSDMD overexpression (Figure 4F). This change in MMP14 expression simultaneously caused corresponding changes in the levels of proteins related to the epithelial‒mesenchymal transition (EMT) (Vimentin, E‐cadherin, N‐cadherin, matrix metalloproteinase 2 (MMP2), matrix metalloproteinase 9 (MMP9), and Snail2 (Figure 4F), indicating that the interaction between GSDMD and MMP14 regulates the EMT process in OSCC. Additionally, altered MMP14 expression synchronously led to changes in the expression and secretion of MMP2 and MMP9 (Figure 4G,H). We also detected the expression of GSDMD, MMP14, and EMT‐related proteins in OSCC cells after cisplatin treatment, and the results were similar to those after GSDMD overexpression (Figure S10A, Supporting Information). Moreover, after MMP14 was silenced in OSCC cells (Figure S11A, Supporting Information), the expression and secreted levels of EMT‐related proteins changed, which was consistent with the results of GSDMD silencing (Figure S10B–D, Supporting Information), accompanied by decreases in the migration and invasion of OSCC cells (Figure S10E,F, Supporting Information).

**Figure 5:**
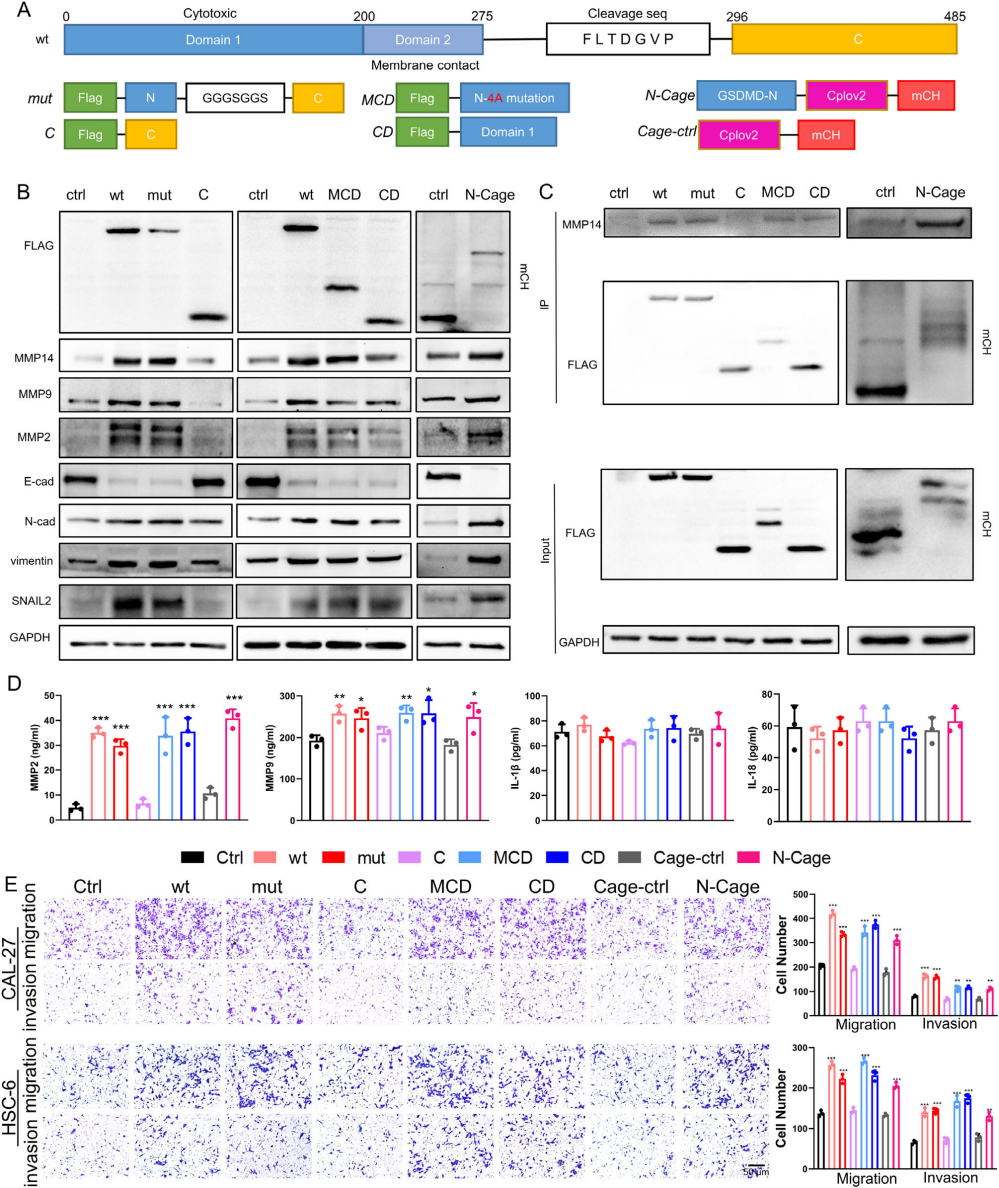
GSDMD regulates MMP14 and the EMT process through its N‐terminal domain to promote the migration and invasion of oral squamous cell carcinoma. A) Structure of GSDMD and design of plasmids to overexpress different mutants and domains of GSDMD. B) Overexpression of different structural domains of GSDMD, including GSDMD‐wt, mut, MCD, CD, and N‐Cage, upregulated MMP14 expression and regulated the expression of EMT‐related proteins, whereas the C‐terminal domain failed to upregulate MMP14 expression. C) Coimmunoprecipitation (co‐IP) revealed that GSDMD‐wt, mut, MCD, CD, and N‐Cage interacted with MMP14, whereas the C‐terminal domain did not bind to MMP14. D) GSDMD‐wt, GSDMD‐mut, MCD, CD, and N‐Cage upregulated the secretion of MMP2 and MMP9 but did not significantly alter IL‐1β or IL‐18 secretion. E) Transwell assay results showing that overexpression of GSDMD‐wt, mut, MCD, CD, or N‐Cage enhanced the migration and invasion abilities.

We explored the specific structural domains involved in the interaction of GSDMD with MMP14 to further investigate why the classical pyroptotic protein GSDMD can exert a prometastatic effect on OSCC. We designed wild‐type GSDMD (wt), GSDMD cleavage site mutant (mut), GSDMD‐C‐terminal (C), N‐terminal with membrane‐binding domain mutant (MCD), and N‐terminal cytotoxic domain (CD) plasmids (**Figure** **5**A) to determine which domain of GSDMD regulates MMP14 expression and tumor metastasis in OSCC. We found that both GSDMD‐wt and GSDMD‐mut overexpression promoted MMP14 expression and the expression and secretion of EMT‐related proteins but did not increase IL‐1β or IL‐18 secretion (Figure 5B,D), indicating that the regulatory effect of GSDMD on MMP14 does not depend on the activation of pyroptosis. Moreover, the overexpression of GSDMD‐C did not promote MMP14 expression, whereas the overexpression of GSDMD‐MCD or CD led to changes in the expression and secretion of EMT‐related proteins, similar to the effects of GSDMD‐wt overexpression (Figure 5B,D). We subsequently used the light‐controlled protein cpLOV2^[^ ^25^ ^]^ to block the membrane‐binding function of the N‐terminal domain of GSDMD and verify whether the regulation of MMP14 expression by the N‐terminal domain of GSDMD is associated with the pyroptosis pathway (Figure 5A), and found that the overexpression of N‐Cage still regulated MMP14 and EMT‐related protein expression and secretion (Figure 5B,D). Through coimmunoprecipitation experiments, we further verified that GSDMD‐wt, mut, MCD, CD, and N‐Cage could interact with MMP14, while the C‐terminal domain failed to bind to MMP14 (Figure 5C). Additionally, we further examined the expression of the GSDMD‐N‐terminal cleavage fragment, Caspase‐1, and cleaved Caspase‐1. Although GSDMD expression levels were significantly increased following low‐dose cisplatin stimulation, no detectable GSDMD‐N‐terminal cleavage fragment was observed, and neither the levels of Caspase‐1 nor its cleaved fragment changed significantly (Figure S10A, Supporting Information). IL‐1β and IL‐18 secretion profiles also remained stable following low‐dose cisplatin stimulation, with no statistically significant variations observed(Figure S10B, Supporting Information). These results collectively indicate that no prominent pyroptosis occurred in OSCC cells following low‐dose cisplatin treatment. Furthermore, we verified the impact of overexpressing these plasmids on the migration and invasion abilities of OSCC cells and found that the overexpression of wt, mut, MCD, CD, or N‐Cage increased the migration and invasion abilities of OSCC cells, whereas the overexpression of the C‐terminal domain did not significantly enhance these abilities (Figure 5E; Figure S12, Supporting Information). In summary, GSDMD mainly interacts with MMP14 through its N‐terminal cytotoxic domain to increase MMP14 expression levels, thereby activating the EMT process in cells and promoting OSCC metastasis.

We subsequently investigated whether GSDMD cleavage and pyroptosis in OSCC cells influence its interaction with MMP14. The results indicated that while Caspase‐1 and GSDMD exhibited marked functional cleavage with IL‐1β and IL‐18 secretion significantly raised following nigericin‐induced pyroptosis in OSCC cells, the MMP14 expression levels remained unaltered (Figure S10C,D, Supporting Information). These findings reveal that the interaction between GSDMD and MMP14 operates through a novel regulatory mechanism independent of the canonical pyroptosis pathway.

### 2.4 Systemic Delivery of siGSDMD by pH‐Responsive NPs Inhibits OSCC Tumor Growth and Metastasis

**Figure 6:**
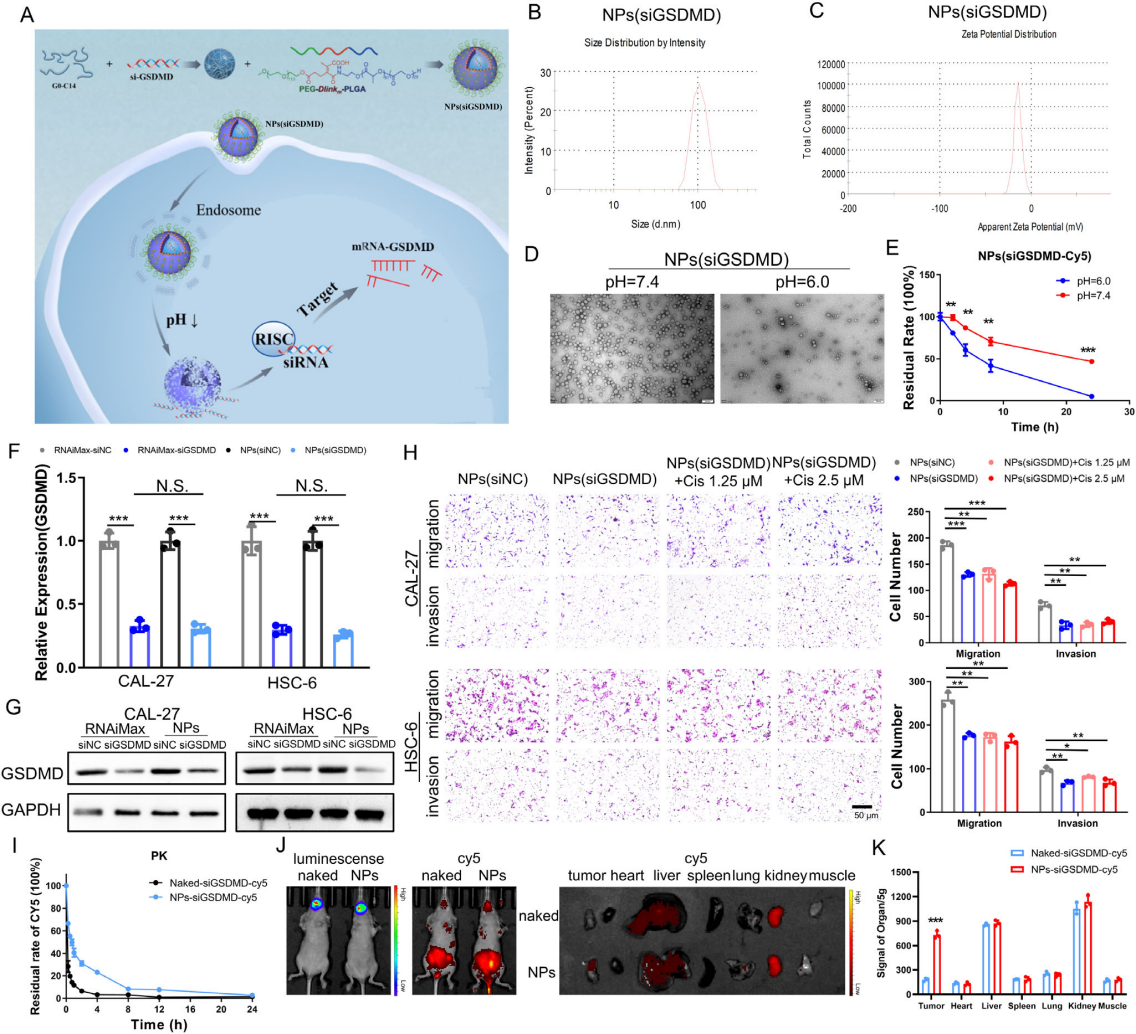
NPs(siGSDMD)‐mediated silencing inhibits the migration and invasion of OSCC cells and its targeted delivery in vivo. A) Schematic diagram of the targeted delivery of siGSDMD by NPs. B) Particle size of NPs(siGSDMD). C) Zeta potential of NPs(siGSDMD). D) Electron microscopic morphology of the NPs(siGSDMD) after treatment at different pH values. E) Release curve of NPs(siGSDMD‐Cy5) after treatment at different pH values. F) In vitro knockdown of GSDMD mRNA levels in CAL‐27 and HSC‐6 cells by NPs(siGSDMD), which effectively downregulated GSDMD. G) In vitro knockdown of GSDMD protein levels in CAL‐27 and HSC‐6 cells by NPs(siGSDMD), which effectively downregulated GSDMD. H) Inhibition of the migration and invasion of CAL‐27 and HSC‐6 cells after GSDMD knockdown by NPs(siGSDMD) and blockade of the effect of cisplatin on promoting the migration and invasion of oral squamous cell carcinoma cells. I) Significant reduction in the blood siGSDMD clearance rate by NP‐mediated delivery of siGSDMD in vivo. J) Enrichment of siGSDMD at tumor sites by NP‐mediated delivery of siGSDMD (luminescence indicates the tumor sites, Cy5 fluorescence indicates the distribution of siGSDMD in vivo). K) Fluorescence intensity detected after organ grinding, showing significantly higher fluorescence intensity per unit mass at tumor sites in the NPs group than in the naked group.

As the abnormal upregulation of GSDMD in OSCC after cisplatin chemotherapy induces tumor metastasis, targeted regulation of GSDMD expression may be an effective strategy for inhibiting postchemotherapy OSCC metastasis. We previously developed a pH‐responsive nanoplatform made with PEG‐Dlinkm‐PLGA and G0‐C14 for siRNA delivery and documented its high efficacy in silencing target gene expression in tumor tissues.^[^ ^26^ ^]^ Therefore, we further used this nanoplatform for the systemic delivery of siGSDMD (**Figure** **6**A). After siGSDMD was loaded into the NPs (denoted NPs(siGSDMD)), the average particle size was ≈100 nm (Figure 6B), with a zeta potential of approximately −15 mV (Figure 6C). In vitro release experiments revealed rapid release of siGSDMD‐cy5 from the NPs under acidic conditions (pH = 6.5), with nearly complete release occurring after 24 h, whereas the release rate decreased under neutral conditions (pH = 7.4), with ≈50% remaining after 24 h (Figure 6D,E). We then silenced GSDMD in OSCC cells using NPs(siGSDMD). The effect of NPs(siGSDMD) on the knockdown of GSDMD in OSCC cells was comparable to that of Lipofectamine RNAiMax (Figure 6F,G), and the migration and invasion of OSCC cells were significantly inhibited after GSDMD was knocked down using NPs, with no recovery after cisplatin treatment (Figure 6H). We further evaluated the in vivo pharmacokinetics and biodistribution of the NPs in vivo. The results showed that the NPs increased the blood drug concentration of the siRNAs and prolonged the in vivo blood circulation time of the siRNAs (Figure 6I). Live imaging and organ grinding analyses revealed that the siRNAs accumulated at the tumor site more effectively in the NPs(siGSDMD) group than in the naked siGSDMD group, indicating the targeted delivery of siGSDMD to the OSCC site (Figure 6J,K).

**Figure 7:**
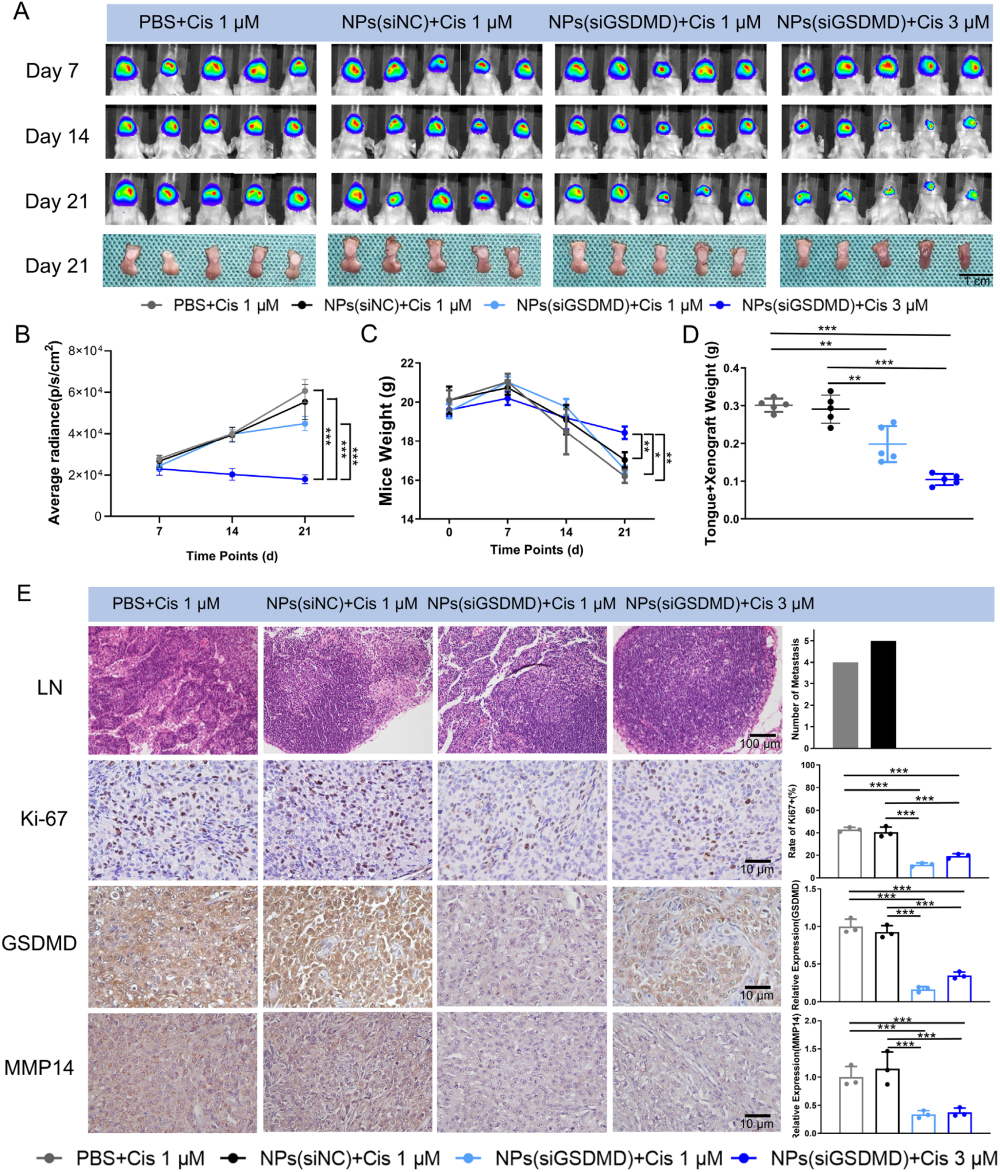
NP‐mediated targeted delivery of siGSDMD inhibited cisplatin‐induced OSCC metastasis in vivo. A) In vivo luminescence imaging and tumor image after combination therapy with NPs and cisplatin in a mouse tongue orthotopic xenograft model. B) Combination therapy with NPs(siGSDMD) and cisplatin effectively reduced the luminescence intensity of xenografts in mice. C) Mice treated with the combination therapy consisting of NPs(siGSDMD) and cisplatin exhibited less weight loss. D) Tumor weights of orthotopic xenografts from mice treated with the combination therapy consisting of NPs(siGSDMD) and cisplatin were lower than those in control mice. E) HE staining showed that combination therapy with NPs(siGSDMD) and cisplatin inhibited cervical lymph node metastasis in mice, while IHC revealed decreased expression levels of Ki‐67, GSDMD, and MMP14 in the tumors.

Furthermore, we assessed the in vivo antitumor metastatic effect of this nanoparticle delivery system, NPs(siGSDMD). We established an orthotopic mouse tongue xenograft model of OSCC and administered concurrent cisplatin chemotherapy and nanoparticle therapy. Compared with those in the control group, mice treated with combination therapy consisting of NPs(siGSDMD) and cisplatin did not exhibit tumor metastasis to the cervical lymph nodes without affecting the efficacy of cisplatin chemotherapy. These findings indicated that the NPs (siGSDMD) inhibited OSCC growth and postchemotherapy tumor metastasis (**Figure** **7**A–E). The immunohistochemistry results showed that the nanoparticles effectively reduced the aberrant expression of GSDMD after cisplatin chemotherapy, simultaneously downregulating MMP14 expression and decreasing the percentage of Ki‐67‐positive tumors (Figure 7E). Additionally, no significant abnormalities were observed in the major organs (i.e., heart, liver, spleen, lung, and kidney) of the mice treated with nanoparticles (Figure S13A, Supporting Information), and liver and kidney indicators, including aspartate aminotransferase (AST), alanine aminotransferase (ALT), albumin (ALB), alkaline phosphatase (ALP), urine acid (UA), and creatinine (CREA), did not change significantly compared with those of the control group (Figure S13B, Supporting Information), demonstrating the good biocompatibility of NPs(siGSDMD). These results indicate that NPs(siGSDMD), as a biological therapy for inhibiting cisplatin chemotherapy‐induced OSCC metastasis, have promising potential for clinical translation.

## 3 Discussion

Neoadjuvant chemotherapy is a crucial component of neoadjuvant therapy; however, currently, a consensus on the optimal dosage for neoadjuvant chemotherapy is lacking. Some scholars advocate for a reduced dose of neoadjuvant chemotherapy to avoid systemic toxicity, thereby preventing adverse effects on patients' overall condition and subsequent surgical treatment.^[^ ^27^, ^28^ ^]^ Conversely, others argue that conventional doses of chemotherapy drugs for neoadjuvant chemotherapy do not lead to greater toxicity and are more conducive to achieving the most ideal cytotoxic effects on tumors, thereby creating better conditions for surgery.^[^ ^29^ ^]^ In our study, we found that low concentrations of cisplatin, a representative platinum‐based drug commonly used in neoadjuvant chemotherapy for oral squamous cell carcinoma (OSCC), increased the migration, invasion, and lymph node metastasis of OSCC. We hypothesized that upon low‐concentration cisplatin stimulation, the cytotoxic effects of chemotherapy drugs on OSCC cells are limited and instead trigger more malignant biological behaviors by activating stress responses in tumor cells, resulting in adverse effects such as tumor lymph node metastasis. This finding suggests that using conventional rather than reduced doses of chemotherapy drugs may be an important measure to prevent such adverse effects during neoadjuvant chemotherapy for OSCC patients. However, further clinical research is needed to validate this hypothesis.

Conversely, several studies have shown that the response of OSCC patients to neoadjuvant chemotherapy is uneven, often manifesting as multifocal and heterogeneous tumor regression.^[^ ^30^ ^]^ This differential response may be due to tumor heterogeneity, leading to scattered internal tissue structures,^[^ ^31^ ^]^ especially in large tumors in advanced stages, where an irregular vascular distribution and lack of homogeneity in the tumor tissue structure may result in a heterogeneous distribution of chemotherapy drugs within the tumor.^[^ ^32^ ^]^ Different tumor sites and different degrees of differentiation of tumor cells may subsequently exhibit varying degrees of stress response after stimulation with chemotherapy drugs at different concentrations, leading to the enhanced migration and invasion capabilities of some tumor cells and ultimately the lymph node metastasis of OSCC, despite an overall reduction in the tumor burden.^[^ ^33^ ^]^ A partial EMT in OSCC not only participates in the process of cervical lymph node metastasis but also contributes to the occurrence of cisplatin resistance, further corroborating the clinical phenomenon of a poor lymph node response during neoadjuvant therapy for OSCC.^[^ ^34^ ^]^ In light of this speculation, simply using conventional doses of chemotherapy drugs may not be sufficient to prevent lymph node metastasis and the progression of OSCC after neoadjuvant chemotherapy. Therefore, further exploration of the regulatory mechanism of cisplatin in the process of OSCC metastasis, identification of key regulatory targets of tumor metastasis, and an exploration of their regulatory mechanisms may provide a theoretical basis for more effective interventions in the neoadjuvant chemotherapy of oral cancer to avoid the occurrence of side effects related to lymph node metastasis in the future.

In our study, we identified the upregulation of GSDMD after neoadjuvant chemotherapy as an important molecular target regulating the metastasis of OSCC. GSDMD is a classic effector protein in the pyroptosis pathway^[^ ^35^ ^]^ that can be recognized and cleaved specifically by Caspase‐1/4/11 to form N‐ and C‐terminal domains. The cleaved N‐terminal domain of GSDMD forms an oligomer that binds to the cell membrane, causing membrane perforation and inducing programmed cell death.^[^ ^36^, ^37^ ^]^ GSDMD is widely considered related to the regulation of chemotherapy sensitivity in tumors. In recent years, several studies have reported that GSDMD activates various nonclassical pathways, such as activation of the cGAS‐STING pathway^[^ ^38^ ^]^ and the regulation of neutrophil extracellular traps (NETs).^[^ ^39^, ^40^, ^41^ ^]^ In our study, we found that GSDMD upregulated matrix metalloproteinase 14 (MMP14) expression through an interaction with MMP14. MMP14 can further activate tumor epithelial–mesenchymal transition (EMT) by promoting the expression, maturation, and release of MMP2 and MMP9, thereby promoting tumor metastasis.^[^ ^42^, ^43^, ^44^, ^45^ ^]^ By overexpressing different functional domains of GSDMD, we found that GSDMD mainly regulates MMP14 through its N‐terminal cytotoxic domain (residues 1–200). Furthermore, using the light‐controlled protein cpLOV2,^[^ ^25^ ^]^ we blocked the membrane contact function of the N‐terminal domain of GSDMD and thereby inhibited its ability to activate pyroptosis. We found that this domain still plays a role in activating the EMT process in OSCC cells. Therefore, we demonstrated that the regulation of OSCC metastasis by GSDMD does not depend on the traditional pyroptosis pathway but rather on its N‐terminal cytotoxic domain, which interacts with MMP14, revealing a novel regulatory mechanism for activating the EMT process in tumors.

Currently, small‐molecule inhibitors targeting GSDMD act mainly by inhibiting the Caspase 1‐mediated cleavage of GSDMD to suppress pyroptosis but cannot effectively reduce GSDMD expression in OSCC.^[^ ^46^, ^47^ ^]^ Therefore, the use of siRNAs to inhibit GSDMD expression in OSCC is a promising approach. Due to their enhanced permeability and retention characteristics, nanoparticle‐based tumor‐targeted delivery systems can achieve the targeted delivery and release of siRNAs in tumors, making them excellent carriers for precise in vivo siRNA delivery.^[^ ^48^ ^]^ In our study, we further utilized pH‐responsive nanoparticles loaded with a GSDMD siRNA in the tumor microenvironment to successfully knock down GSDMD expression and inhibit tumor metastasis in in vitro and in vivo models.

In summary, in this study, we validated the phenomenon of the metastasis of OSCC induced by low concentrations of cisplatin and proposed that using conventional doses rather than low doses of chemotherapy drugs in neoadjuvant therapy for OSCC may help improve treatment outcomes and reduce neoadjuvant chemotherapy‐induced tumor metastasis. Through high‐throughput sequencing, we identified the key regulatory target of cisplatin‐induced OSCC metastasis, GSDMD, and elucidated that the regulation of OSCC metastasis by GSDMD does not depend on its classical pyroptosis pathway but rather on a new regulatory mechanism mediated by its interaction with MMP14, thereby inducing the tumor EMT and promoting metastasis. Moreover, using NPs(siGSDMD), we effectively suppressed OSCC metastasis without affecting the cytotoxic effects of cisplatin. This study provides new treatment options and a theoretical basis for improving the efficacy of neoadjuvant chemotherapy for OSCC and suppressing its side effects.

## 4 Experimental Section

### Antibodies and Reagents

Antibodies specific for DYKDDDDK (FLAG tag, #14 793) and GAPDH (#5174), anti‐rabbit IgG (#7074), and anti‐mouse IgG (#7076) were purchased from Cell Signaling Technology. Antibodies specific for GSDMD (20770‐1‐AP), MMP14 (14552‐1‐AP), vimentin (60330‐1‐Ig), E‐cadherin (20874‐1‐AP), N‐cadherin (22018‐1‐AP), MMP2 (10373‐1‐AP), MMP‐9 (10375‐2‐AP), SNAIL2 (13099‐1‐AP), ubiquitin (10201‐2‐AP), Ki67 (27309‐1‐AP), and mCherry (26765‐1‐AP) were purchased from Proteintech.

Cisplatin, docetaxel and 5‐fluorouracil were formulated as 100 µM stock solutions and stored at −30 °C for later use. At the time of use, the preparation ratio was calculated, and the stock solution was diluted with culture medium to the final concentration for cell pretreatment.

### Patients and Tissue Sections

The patients and tissue specimens used in this study were obtained from Sun Yat‐sen Memorial Hospital of Sun Yat‐sen University between 2020 and 2023. All human subjects involved in this study signed informed consent forms according to the international ethical guidelines for biomedical research involving human subjects, and the study received approval from the Ethics Committee of Sun Yat‐sen Memorial Hospital. The inclusion criteria included a pathological diagnosis of OSCC and receiving neoadjuvant chemotherapy (TPF chemotherapy: 75 mg/m^2^ docetaxel (T), 75 mg/m^2^ cisplatin (P) and 750 mg/m^2^ 5‐fluorouracil (F) intravenously on Days 1 and 21) and surgery 14 days after the last dose of neoadjuvant chemotherapy. Patients were excluded from the study if they had been diagnosed with multiple cancers or other severe diseases. The characteristics of OSCC patients, including age, sex, tumor differentiation, lymphatic metastasis status and clinical stage, were recorded. In addition, tumor samples were also collected for high‐throughput sequencing, and tissue sections were collected for HE staining and immunohistochemistry.

### Cell Culture

Two oral squamous cell carcinoma cell lines (CAL‐27 and HSC‐6) and one instrumental cell line (HEK‐293T) were used in this study. All cell lines were purchased from the ATCC cell repository. CAL‐27, HSC‐6, and HEK‐293T cells were cultured in DMEM supplemented with 10% FBS and 1% penicillin‒streptomycin. All the cells were cultured at 37 °C in a 5% CO_2_ environment.

### Chemotherapy Drug Treatment of OSCC Cells

The cells were treated with different concentrations of chemotherapeutic drugs: cisplatin (Cis) (0, 1.25, 2.5, 5, or 10 µM); docetaxel (Doce) (0, 1.25, or 2.5 µM); and 5‐fluorouracil (5‐FU) (0, 100, or 200 µM). After 48 h of chemotherapy, the cells were used for subsequent experiments.

### Wound Healing Assay

The cells were seeded in 6‐well plates at a density of 8 × 10^5^ cells well^−1^ and cultured overnight. Using a 200 µl pipette tip, scratches were made uniformly in the center of each well after the cells formed a monolayer. After washes with PBS, serum‐free DMEM was added, and the degree of cell migration at the scratch site was photographed under a microscope every 12 h.

### Transwell Assay

Cell migration and invasion assays were performed using 24‐well plates with 8 µm Transwell chambers. The cells were seeded in the upper chamber (preseeded with a layer of Matrigel matrix in the invasion assay) with serum‐free medium (30 000 cells per chamber), and medium containing 10% FBS DMEM was added to the lower chamber. After 24–48 h of incubation, the cells were fixed with 4% paraformaldehyde, stained with 0.1% crystal violet, and observed and photographed under a microscope.

### Colony Formation Assay

The cells were cultured in 6‐well plates at a density of 1000 cells per well. After 14 days, cell colonies had formed. Colonies were fixed with 4% paraformaldehyde for 15 min, stained with 0.1% crystal violet, air‐dried, and photographed.

### Cell Proliferation Assay

The cells were cultured in 96‐well plates at a density of 1000 cells per well. After 24 h, CCK‐8 reagent was added to each well, and the plates were incubated at 37 °C for 2 h. The absorbance at 450 nm was measured using a microplate reader.

### Cellular Chemotherapy Sensitivity Assay

The cells were cultured in 96‐well plates at a density of 10 000 cells per well. After 24 h, the cells were treated with different concentrations of chemotherapy drugs for 48 h. CCK‐8 reagent was added to each well, and the plates were incubated at 37 °C for 2 h. The absorbance at 450 nm was measured using a microplate reader.

### High‐Throughput RNA Sequencing

After total RNA was extracted, RNA integrity was assessed using an RNA Nano 6000 Assay Kit and a Bioanalyzer 2100 system. According to the manufacturer's instructions, ribosomal RNA (rRNA) was removed from the total RNA using a ribosomal RNA removal kit. The RNA was subsequently fragmented into 250–300 bp fragments, followed by reverse transcription into first‐strand cDNA using fragmented RNA and dNTPs. The RNA was then degraded using RNase H, and second‐strand cDNA was synthesized using DNA polymerase I and dNTPs. Sequencing adapters were ligated to the cDNA, followed by PCR amplification using Phusion high‐fidelity DNA polymerase, universal PCR primers, and index (X) primers.

The libraries were sequenced using the Illumina NovaSeq platform, and a bioinformatics analysis was conducted by IGE Biotechnology. HISAT was chosen as the mapping tool because it can generate a splice junction database based on a gene model annotation file, providing better mapping results than other nonsplice mapping tools. FeatureCounts v1.6.0 was used to calculate the read counts mapped to each gene. The fragments per kilobase of transcript per million mapped reads (FPKM) for each gene were subsequently calculated based on the gene's length and the read counts mapped to the gene. The read counts for each sequencing library were adjusted by a scaling normalization factor using the edgeR package. A differential expression analysis between the two conditions was then performed using the edgeR package (version 3.18.1), with a significance threshold set at a P value less than 0.05 and an absolute fold change of 2. A Gene Ontology analysis was subsequently conducted for the differentially expressed genes.

### Transient Transfection of siRNAs

The siRNAs used in this study were designed and obtained from IGE Biotechnology. The transfection of siRNAs and plasmids was performed via Lipofectamine RNAiMax reagent. Subsequent experiments were conducted 48 h after transfection.

**Table:** 

Gene		Forward	Reverse
GSDMD	si1	CCCGUUAUAAGUGUGUCAATT	UUGACACACUUAUAACGGGTT
	si2	GCACCUCAAUGAAUGUGUATT	UACACAUUCAUUGAGGUGCTT
CCND1	si1	AGACCAUCCGCAAGCAUGCTT	GCAUGCUUGCGGAUGGUCUTT
	si2	AACGCACUUUCUUUCCAGATT	UCUGGAAAGAAAGUGCGUUTT
BATF2	si1	GCCUCUAAAGGGCUUUAACCUGC	GCAGGUUAAAGCCCUUUAGAGGC
	si2	AGGGCUUUAACCUGCAAUUAAUG	CAUUAAUUGCAGGUUAAAGCCCU
MMP14	si1	GAUGUGGUGUUCCAGACAATT	UUGUCUGGAACACCACAUCTT
	si2	GCAAAUUCGUCUUCUUCAATT	UUGAAGAAGACGAAUUUGCTT

### Stable Cell Line Construction

The target plasmid, packaging plasmid, pMD2G, and psPAX2 were transfected into HEK‐293T cells at a ratio of 1.7:0.5:1 using the Lipofectamine 3000 reagent (Invitrogen). After 48 h, the supernatant containing the lentivirus was collected, filtered through a 0.22 µm filter, and used to infect the target cells. Stable cell lines were selected, and validation was performed via RT‒qPCR. This process led to the construction of stable cell lines expressing GSDMD‐sh, GSDMD‐ov, GSDMD‐wt, mut, C, MCD, CD, or N‐Cage.

### RNA Extraction and qRT‒PCR

Total RNA was extracted using TRIzol, followed by reverse transcription into cDNA using a reverse transcription kit. Real‐time quantitative PCR (qRT‒PCR) experiments were performed using a qRT‒PCR kit and a Roche LightCycler 480 II instrument.

The sequences of the primers used were as follows:

**Table:** 

Gene	Forward	Reverse
GAPDH	GAGTCAACGGATTTGGTCGT	GACAAGCTTCCCGTTCTCAG
GSDMD	TCTGCCCTCAACACTTCTGG	TGCAGCCACAAATAACTCAGC
CCND1	GCTGCGAAGTGGAAACCATC	CCTCCTTCTGCACACATTTGAA
BATF2	GCAGGGGTCTTCCTCTAAGC	GCTGCTGAGAGAGCAGGTTT
MMP14	CTCCGAGTTGGACGATGAGG	TCATGCCTGCACTGTTCATTC

### Western Blot

The cells were lysed with RIPA lysis buffer containing proteinase and phosphatase inhibitors, and the supernatant was collected after centrifugation. The protein concentration was measured using a BCA protein assay kit. Then, SDS‒PAGE was performed on a 10% gel, and the proteins were transferred to a PVDF membrane. The membrane was blocked with 5% BSA and incubated with primary antibody overnight at 4 °C, followed by an incubation with the secondary antibody at room temperature for 1 h. Chemiluminescent signals were detected using an enhanced chemiluminescence (ECL) detection reagent.

### Coimmunoprecipitation (Co‐IP)

The cell lysates were prepared using IP buffer, and the Protein A/G magnetic beads were washed and incubated with primary antibodies at room temperature for 15 min, followed by an overnight incubation with the cell lysates at 4 °C. After the beads were washed, the proteins were eluted with 1× SDS loading buffer for subsequent mass spectrometry analysis and western blotting.

### Influence of Cisplatin Chemotherapy on Tumor Metastasis in a Nude Mouse Tongue Orthotopic Xenograft Model

CAL‐27‐luciferase cells were injected submucosally into the left tongue margin of nude mice at a concentration of 1 × 10^6^ cells to establish a nude mouse tongue xenograft model. Tumor and lymph node metastases were detected weekly using an in vivo imaging system (IVIS). After successful tumor formation in the mouse tongue, different concentrations of cisplatin (0, 1, or 3 µM) were administered via intraperitoneal injection for chemotherapy. The treatment was administered every 2 days for a total of 3 treatments. Two weeks after the completion of treatment, the mice were euthanized, and the tongues and neck lymph nodes were collected. Tissues were fixed with 4% paraformaldehyde for preservation, and some of the xenografts were stored in TRIzol for subsequent high‐throughput sequencing. Sections were prepared via routine paraffin embedding and stained with hematoxylin and eosin (HE) to observe lymph node metastasis. Immunohistochemistry was performed to detect Ki‐67 expression in the tumors.

### Immunohistochemistry

After the paraffin sections were dewaxed and hydrated, antigen retrieval was performed, endogenous peroxidase activity was blocked, and nonspecific binding was blocked. The sections were then incubated with primary antibodies overnight at 4 °C, followed by an incubation with a biotinylated goat anti‐mouse/rabbit IgG polymer at room temperature for 15 min and then with horseradish peroxidase‐labeled streptavidin working solution at room temperature for 10 min. DAB chromogen solution was used for visualization, followed by counterstaining with hematoxylin, dehydration, clearing, and sealing with neutral resin. The sections were observed and photographed under a microscope.

### Effect of GSDMD on Tumor Metastasis in a Nude Mouse Tongue Orthotopic Xenograft Model

Stable GSDMD‐sh and GSDMD‐ov cell lines were used to construct a nude mouse orthotopic xenograft model. Tumor and lymph node metastases were monitored weekly using an in vivo imaging system (IVIS). Three weeks after tumor formation, the mice were euthanized, and the tongues and neck lymph nodes were collected. Tissues were fixed with 4% paraformaldehyde for preservation. Tissue sections were prepared via routine paraffin embedding and stained with HE to observe lymph node metastasis. Immunohistochemistry was performed to detect GSDMD and Ki67 expression in the tumors.

### Preparation of pH‐Responsive Nanoparticles (NPs)

PEG‐Dlinkm‐PLGA and G0‐C14 were dissolved in DMF. G0‐C14 and siRNA were mixed with PEG‐Dlinkm‐PLGA. The mixture was slowly added to deionized water to form nanoparticles through self‐assembly. The particle size and zeta potential were measured via dynamic light scattering, and the morphology of the nanoparticles was observed using a transmission electron microscope.

### NP Pharmacokinetics

Healthy BALB/c nude mice were randomly divided into two groups (n = 3) and intravenously injected with 1 nmol of naked siGSDMD‐Cy5 or NPs(siGSDMD)‐Cy5. At predetermined time intervals, blood (20 µL) was collected from the orbital venous plexus, and the fluorescence intensity of Cy5 was detected with a microplate reader.

### NP Biodistribution

After the nude mouse tongue orthotopic xenograft model was established using CAL‐27‐luciferase cells, the mice were randomly divided into two groups (n = 3) and intravenously injected with 1 nmol of naked siGSDMD‐Cy5 or NPs(siGSDMD)‐Cy5. Twenty‐four hours after the injection, the mice were imaged using an IVIS. The fluorescence intensity of the organs and tumors was measured, and the fluorescence intensity of Cy5 per unit mass was detected with a microplate reader after the organs and tumors were ground.

### Combination Therapy with NPs and Cisplatin Chemotherapy in a Nude Mouse Tongue Orthotopic Xenograft Model

An orthotopic nude mouse tongue xenograft model was established using CAL‐27‐luciferase cells, and after successful tumor formation in the mouse tongue, antitumor nanoparticle treatment combined with cisplatin chemotherapy was administered. The mice were divided into four treatment groups: PBS + 1 µM Cis, 1 µM NPs(siNC) + 1 µM Cis, 1 µM NPs(siGSDMD) + 1 µM Cis, and 3 µM NPs(siGSDMD) + 3 µM Cis. The mice were intravenously injected with NPs loaded with 1 nmol of siRNA along with cisplatin chemotherapy through intraperitoneal injection every two days for a total of three treatments. Mouse body weight, tumor size, and lymph node metastasis status were monitored. Two weeks after the completion of treatment, the mice were euthanized, and the tongues and neck lymph nodes were collected. Tissues were fixed with 4% paraformaldehyde for preservation. Sections were prepared via routine paraffin embedding, and HE staining was performed to observe lymph node metastasis. Immunohistochemistry was performed to detect GSDMD, Ki67, and MMP14 expression in the tumors.

### Statistical Analysis

Student's t test was used to compare differences in quantitative data between groups. A correlation analysis was performed to examine the relationships between GSDMD expression levels and the clinical data of patients (McNemar's test for paired samples and Pearson's chi‐square test for unpaired samples). GraphPad Prism was used for graphing, plotting, and statistical tests. Unless stated otherwise, quantitative data are presented as the means ± standard errors of the means (S.E.M.s). A threshold of α = 0.05 was set for the statistical analysis (*P < 0.05; **P < 0.01; and ***P < 0.001).

### Ethical Approval

This research was conducted in accordance with international guidelines and the ethical standards outlined in the Declaration of Helsinki. This study was approved by the Sun Yat‐sen Memorial Hospital Institutional Review Board (Approval No. SYSKY‐2023‐493‐01). All animal experiments were conducted according to the Ministry of Health national guidelines for the housing and care of laboratory animals and were performed in accordance with institutional regulations after review and approval by the Institutional Animal Care and Use Committee at Sun Yat‐sen University (Approval No. SYSU‐IACUC‐2020‐B0855).

## Conflict of Interest

The authors declare no conflict of interest.

## Author Contributions

Z.H. and Q.J. contributed to this work equally. Z.Q.H. and X.D.X. performed conceptualization; Z.X.H., Q.M.J. and N.L. performed methodology; Z.X.H., Q.M.J., Q.Y.Z., Y.W. and Y.P.W. performed investigation; Z.X.H., Q.M.J., R.C. and X.D.X. wrote the original draft; Z.Q.H. and Z.X.H. wrote reviewed and edited; Z.X.H., X.D.X. and Z.Q.H. performed funding acquisition; Q.Y.Z. and X.R. provided resources; X.D.X. and Z.H.H performed supervision.

## Supporting information



Supporting Information

Supporting Information

Supporting Information

## Data Availability

The data that support the findings of this study are available from the corresponding author upon reasonable request.
