# Supplementary material for: Neoadjuvant Chemotherapy With Cisplatin Up‐Regulates GSDMD to Enhance Oral Squamous Cell Carcinoma Metastasis Through MMP14‐Mediated EMT Activation
Source: Adv Sci (Weinh). 2025 Apr 3;12(25):2501149. doi: 10.1002/advs.202501149 (PMC12224931; doi:10.1002/advs.202501149)
Supplement: Supplementary file 1 — Supporting Information [file ADVS-12-2501149-s001.pdf]

ADVANCED SCIENCE
Open Access

Supporting Information

for *Adv. Sci.*, DOI 10.1002/advs.202501149

Neoadjuvant Chemotherapy With Cisplatin Up-Regulates GSDMD to Enhance Oral Squamous Cell Carcinoma Metastasis Through MMP14-Mediated EMT Activation

Zixian Huang, Qiming Jiang, Qianyu Zhang, Nan Lu, Xi Rui, Rui Chen, Yan Wang, Yuepeng Wang, Xiaoding Xu and Zhiquan Huang**

Figure 2

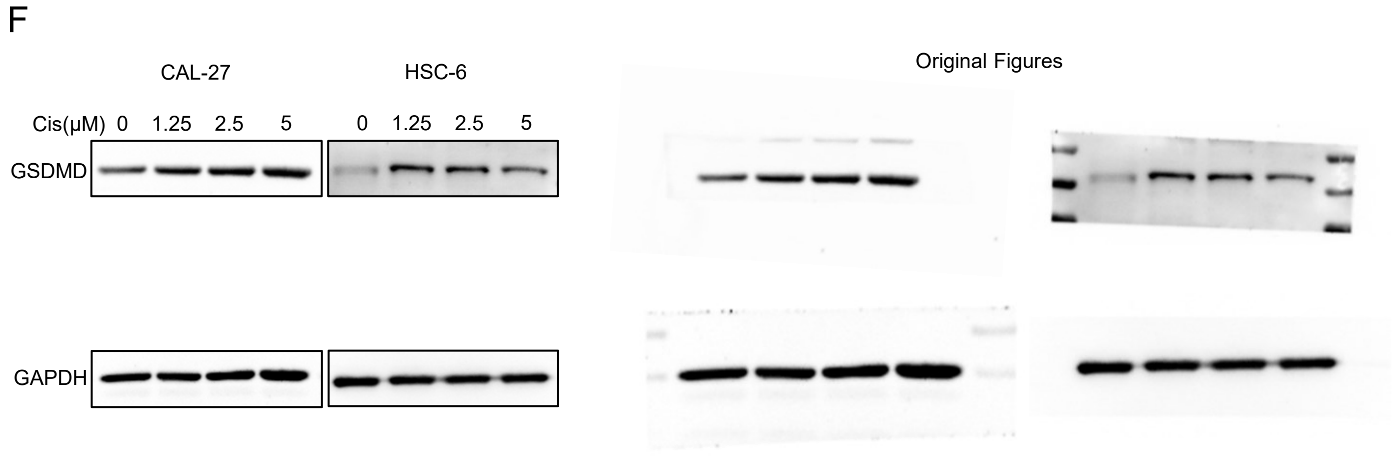
F
CAL-27
HSC-6
Cis(μM) 0 1.25 2.5 5
0 1.25 2.5 5
GSDMD
GAPDH
Original Figures

Fig 3

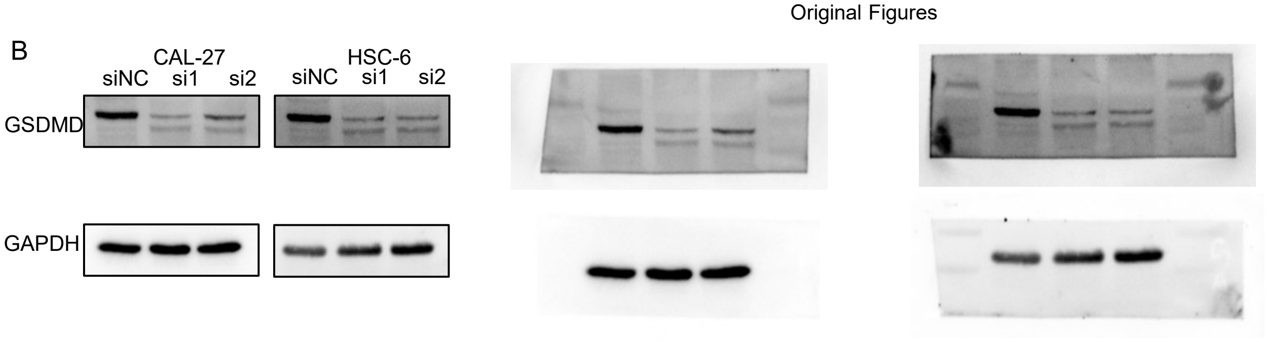
Original Figures
B
CAL-27
HSC-6
siNC
si1
si2
siNC
si1
si2
GSDMD
GAPDH

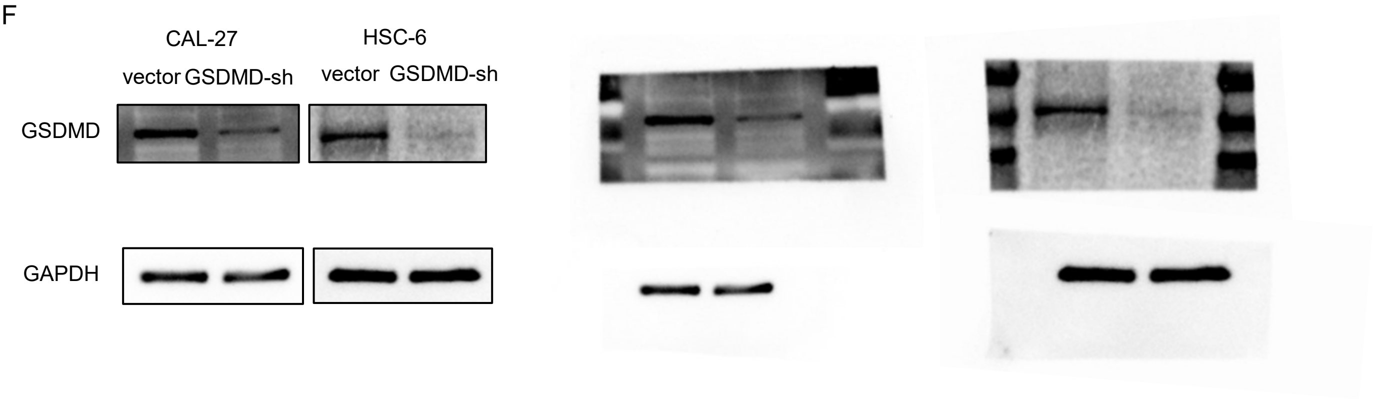
F
CAL-27
HSC-6
vector
GSDMD-sh
vector
GSDMD-sh
GSDMD
GAPDH

Fig 4.

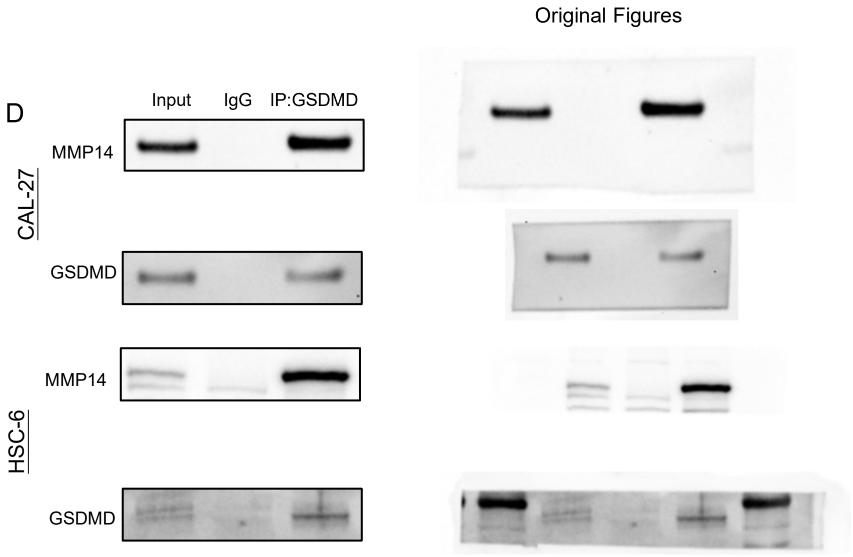

Original Figures
D
Input
IgG
IP:GSDMD
MMP14
CAL-27
GSDMD
MMP14
HSC-6
GSDMD

Fig 4.

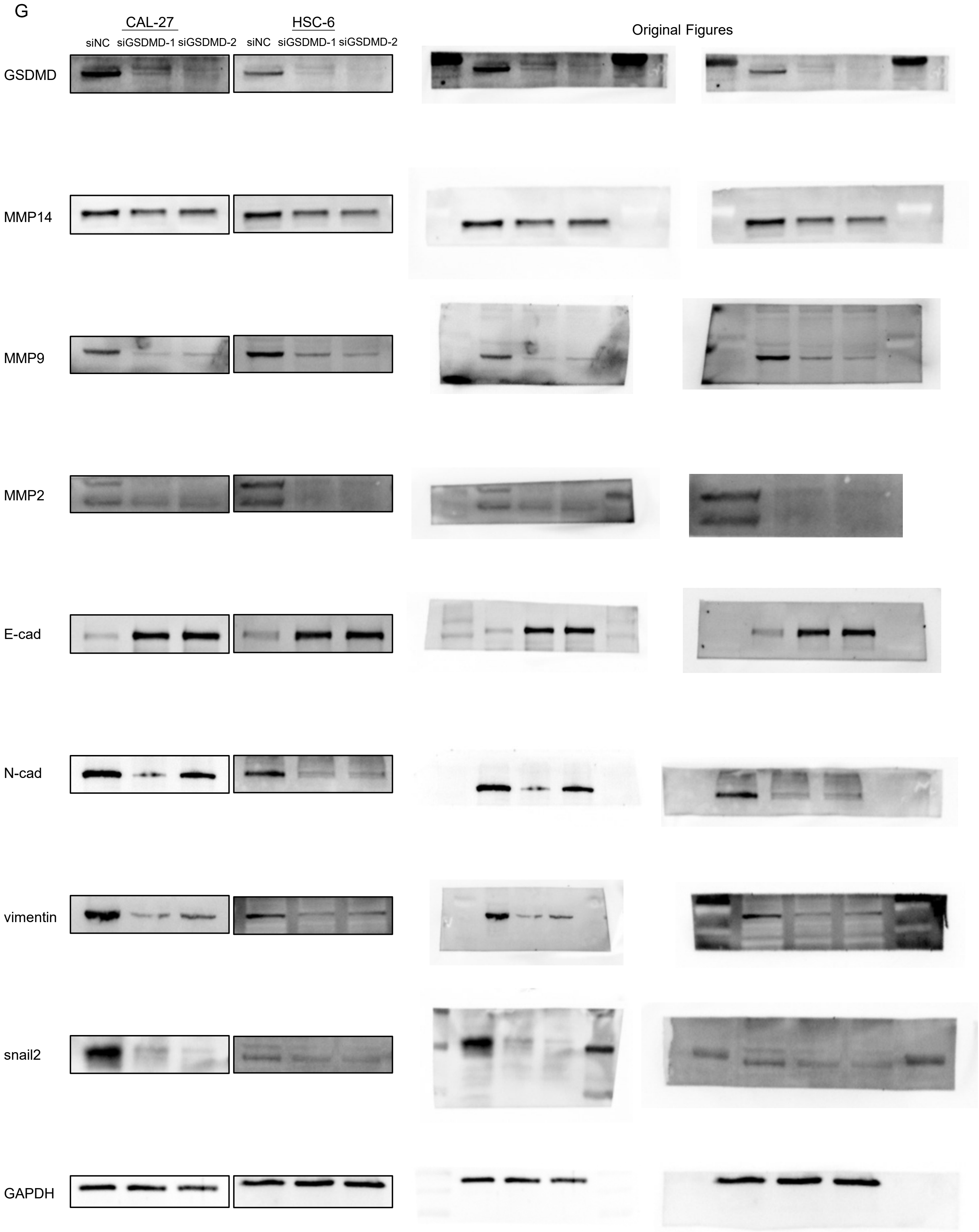
G
CAL-27
HSC-6
siNC
siGSDMD-1
siGSDMD-2
siNC
siGSDMD-1
siGSDMD-2
Original Figures
GSDMD
MMP14
MMP9
MMP2
E-cad
N-cad
vimentin
snail2
GAPDH

Fig 4.

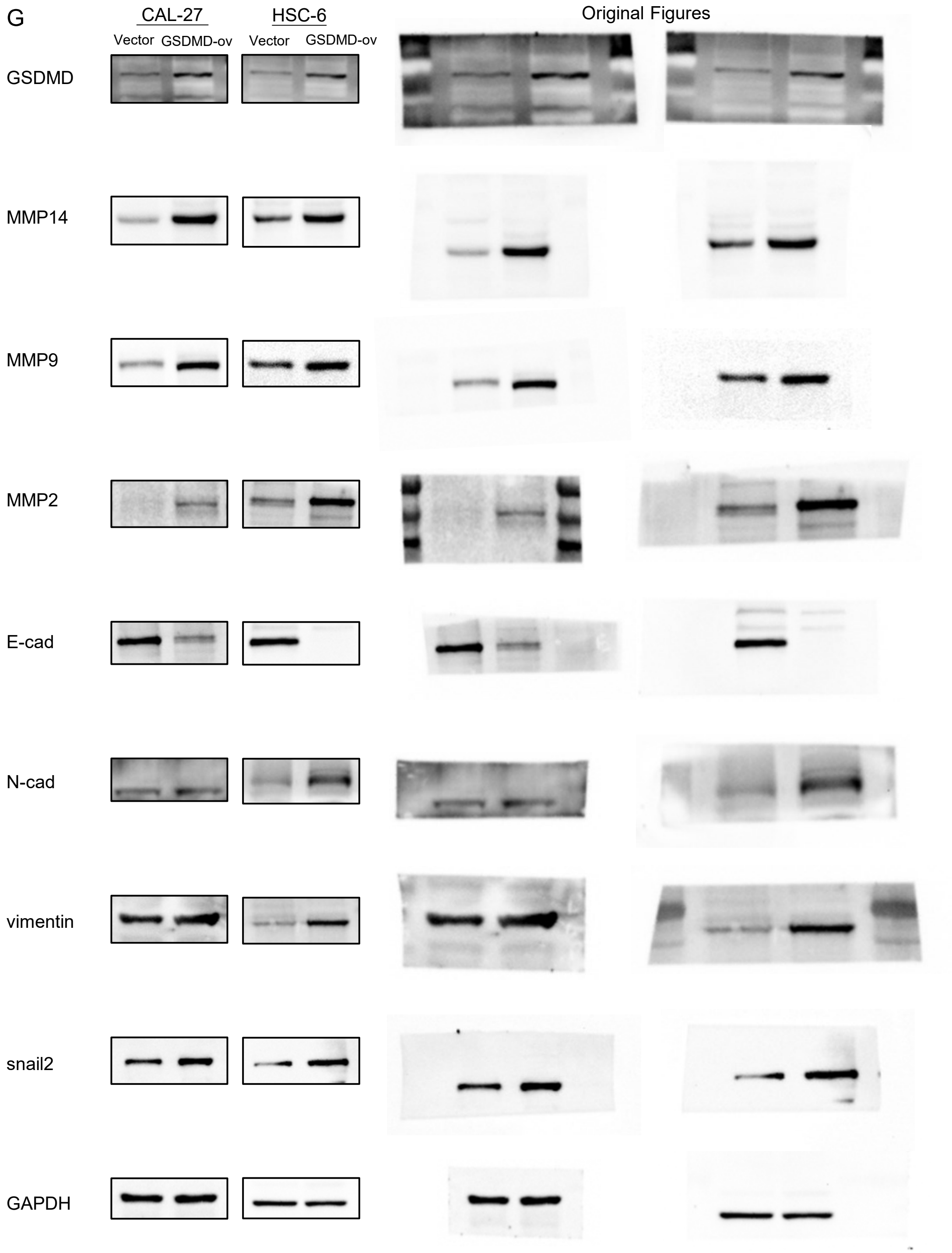
G
CAL-27
HSC-6
Vector
GSDMD-ov
Vector
GSDMD-ov
Original Figures
GSDMD
MMP14
MMP9
MMP2
E-cad
N-cad
vimentin
snail2
GAPDH

Fig 5.

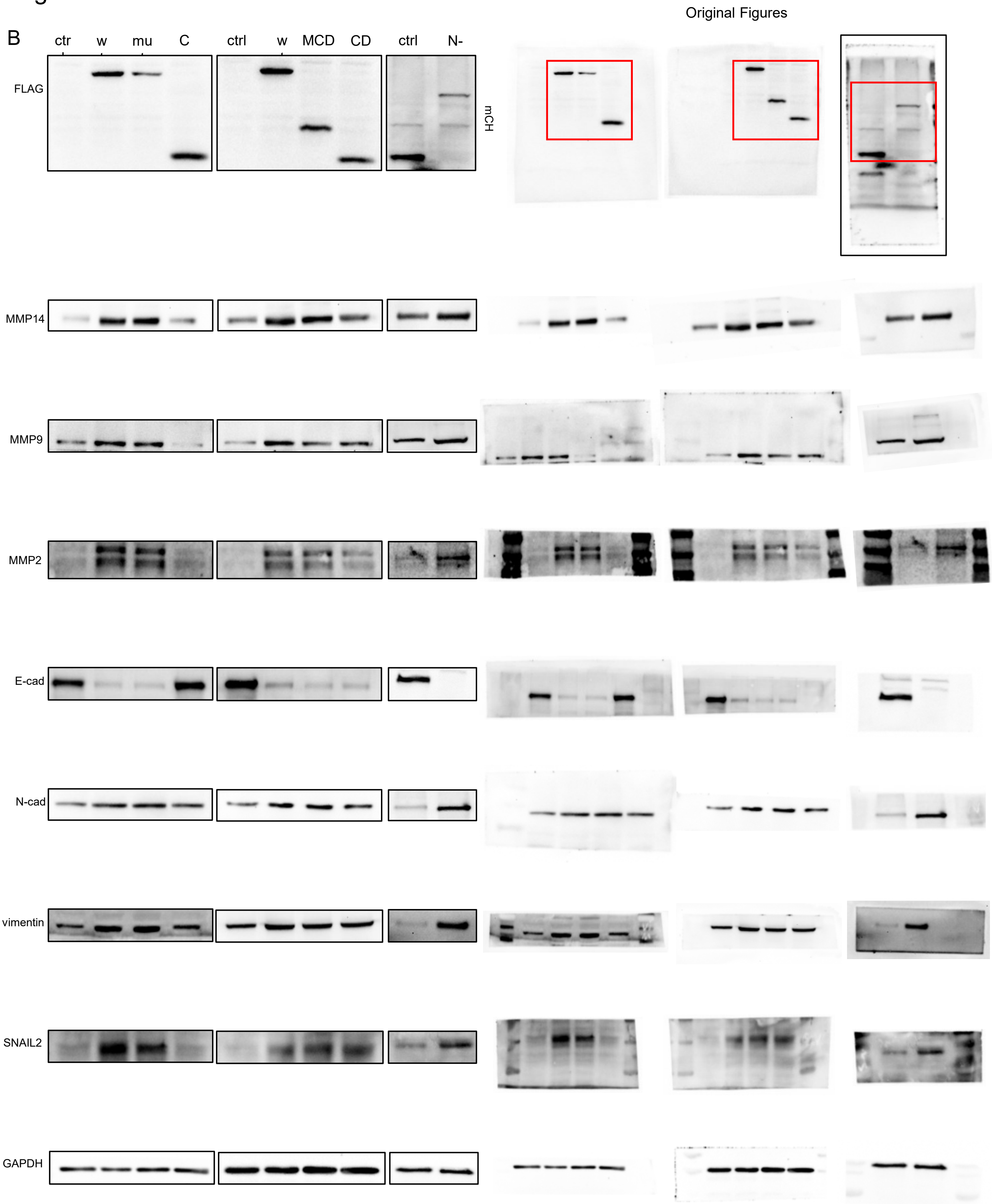
Original Figures
B
ctr
w
mu
C
ctrl
w
MCD
CD
ctrl
N-
FLAG
mCH
MMP14
MMP9
MMP2
E-cad
N-cad
vimentin
SNAIL2
GAPDH

Fig 5.

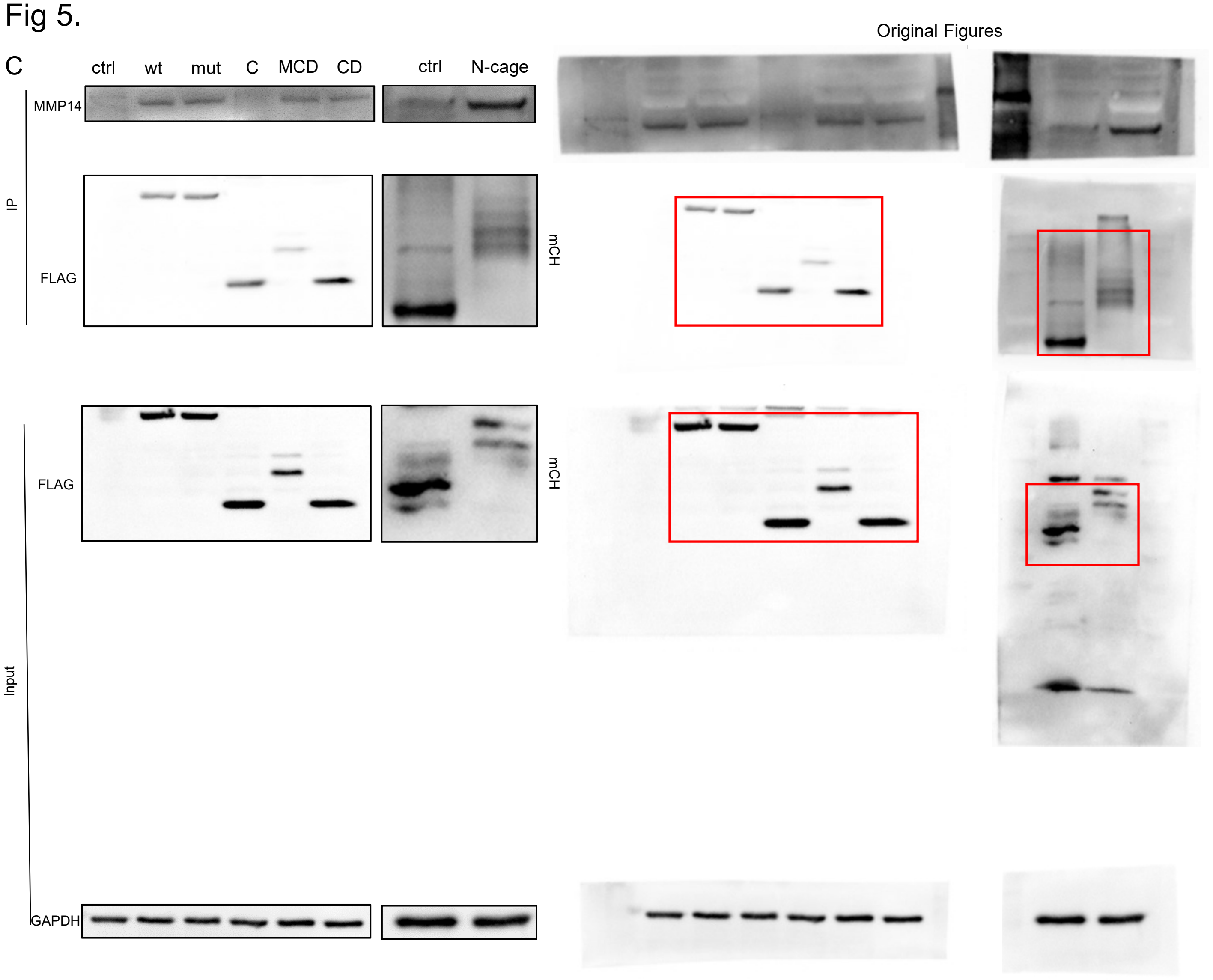
C
ctrl
wt
mut
C
MCD
CD
ctrl
N-cage
Original Figures
MMP14
IP
FLAG
mCH
Input
FLAG
mCH
GAPDH

Fig 6.

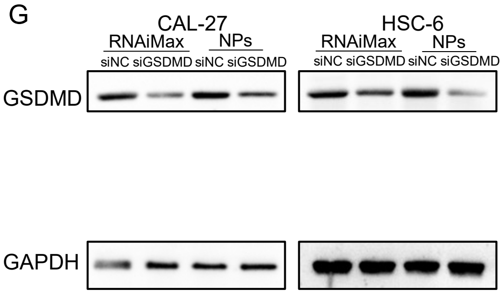

Original Figures

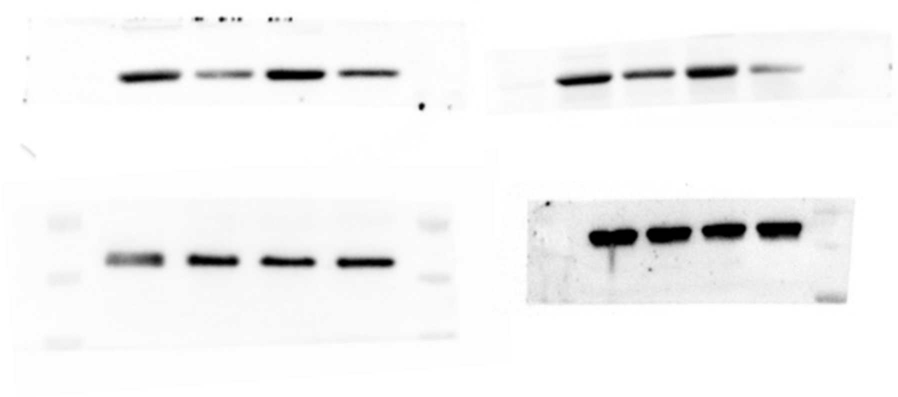

Figure s3.

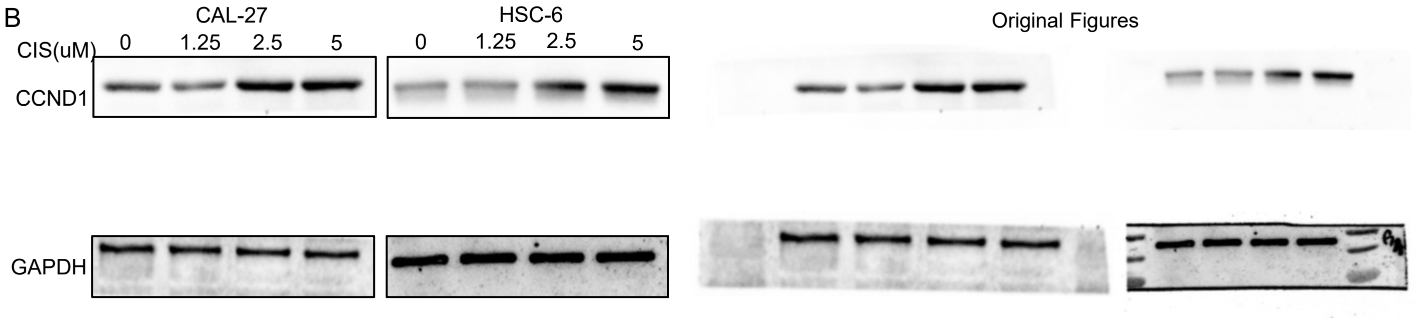
B
CAL-27
HSC-6
CIS(uM)
0
1.25
2.5
5
0
1.25
2.5
5
CCND1
GAPDH
Original Figures

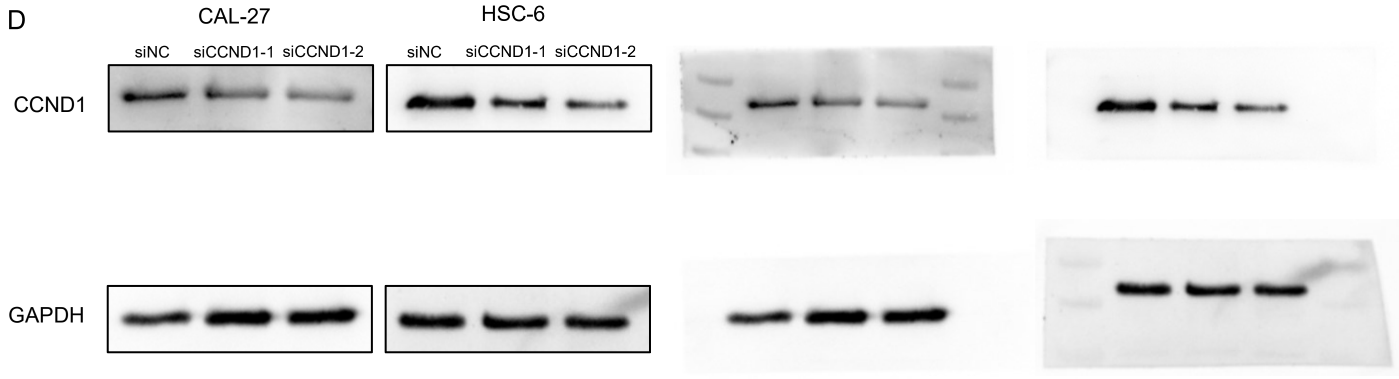
D
CAL-27
HSC-6
siNC
siCCND1-1
siCCND1-2
siNC
siCCND1-1
siCCND1-2
CCND1
GAPDH

Figure s4.

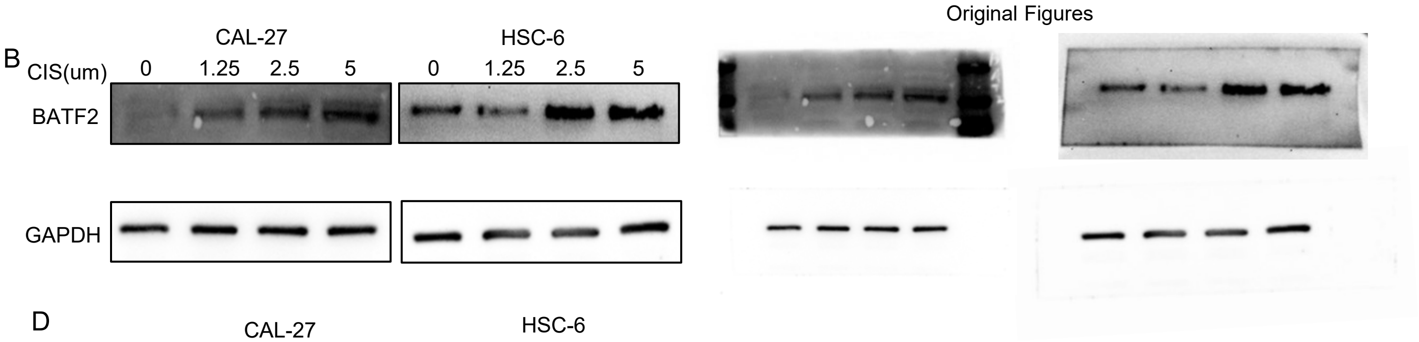

B
CAL-27
HSC-6
CIS(um)
0
1.25
2.5
5
0
1.25
2.5
5
BATF2
GAPDH
Original Figures

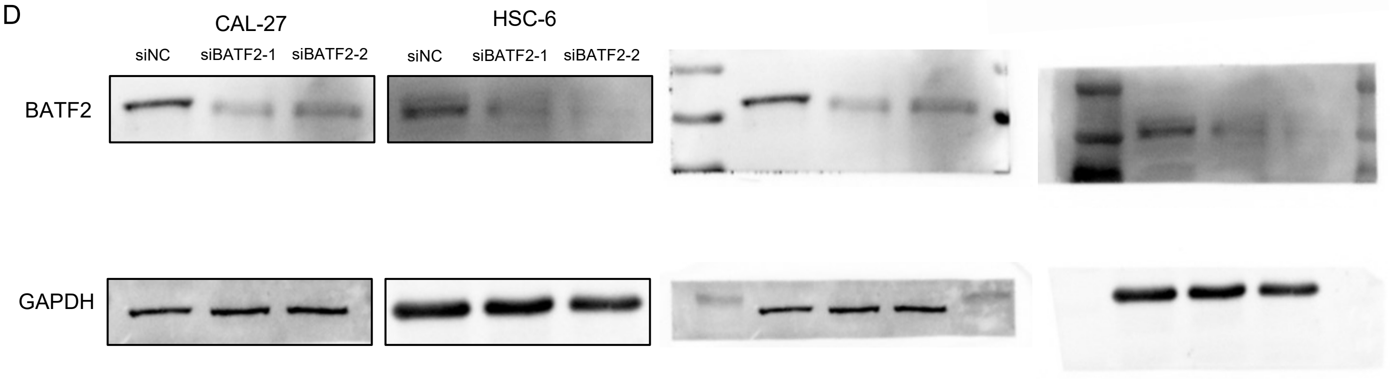

D
CAL-27
HSC-6
siNC
siBATF2-1
siBATF2-2
siNC
siBATF2-1
siBATF2-2
BATF2
GAPDH

Fig s8.

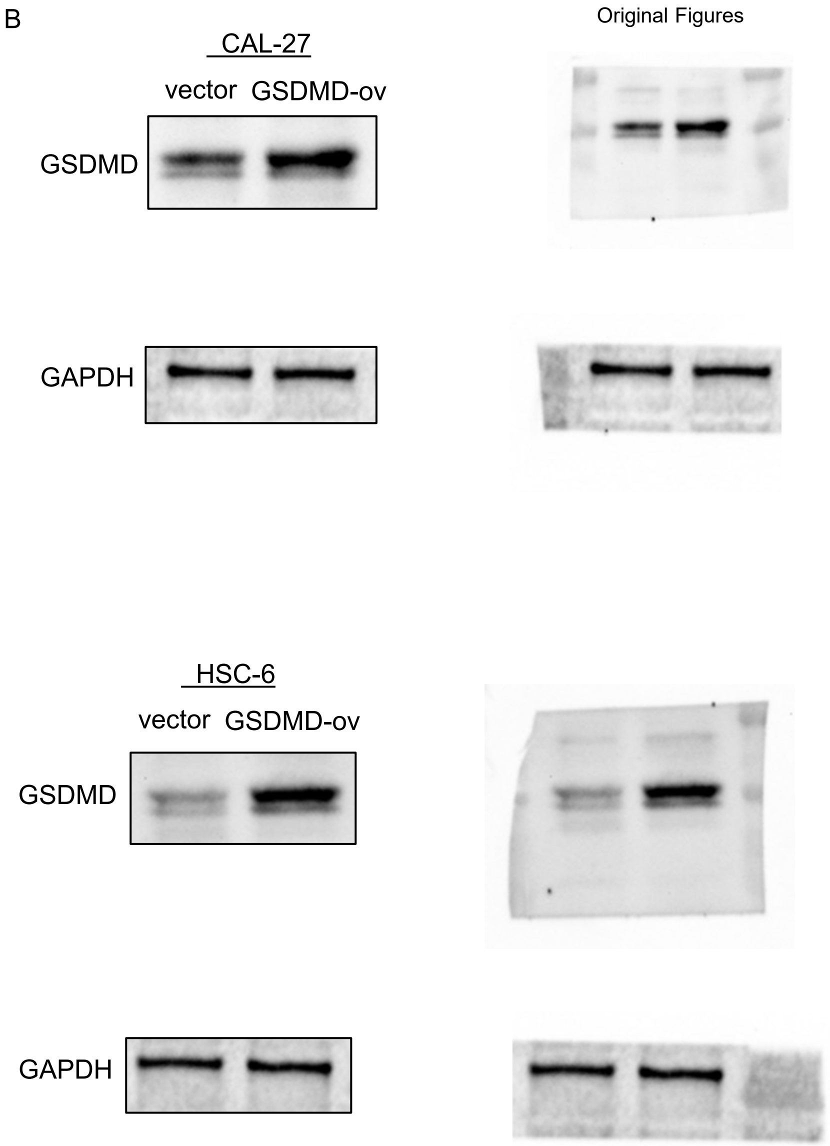
B
Original Figures
CAL-27
vector
GSDMD-ov
GSDMD
GAPDH
HSC-6
vector
GSDMD-ov
GSDMD
GAPDH

Fig s9.

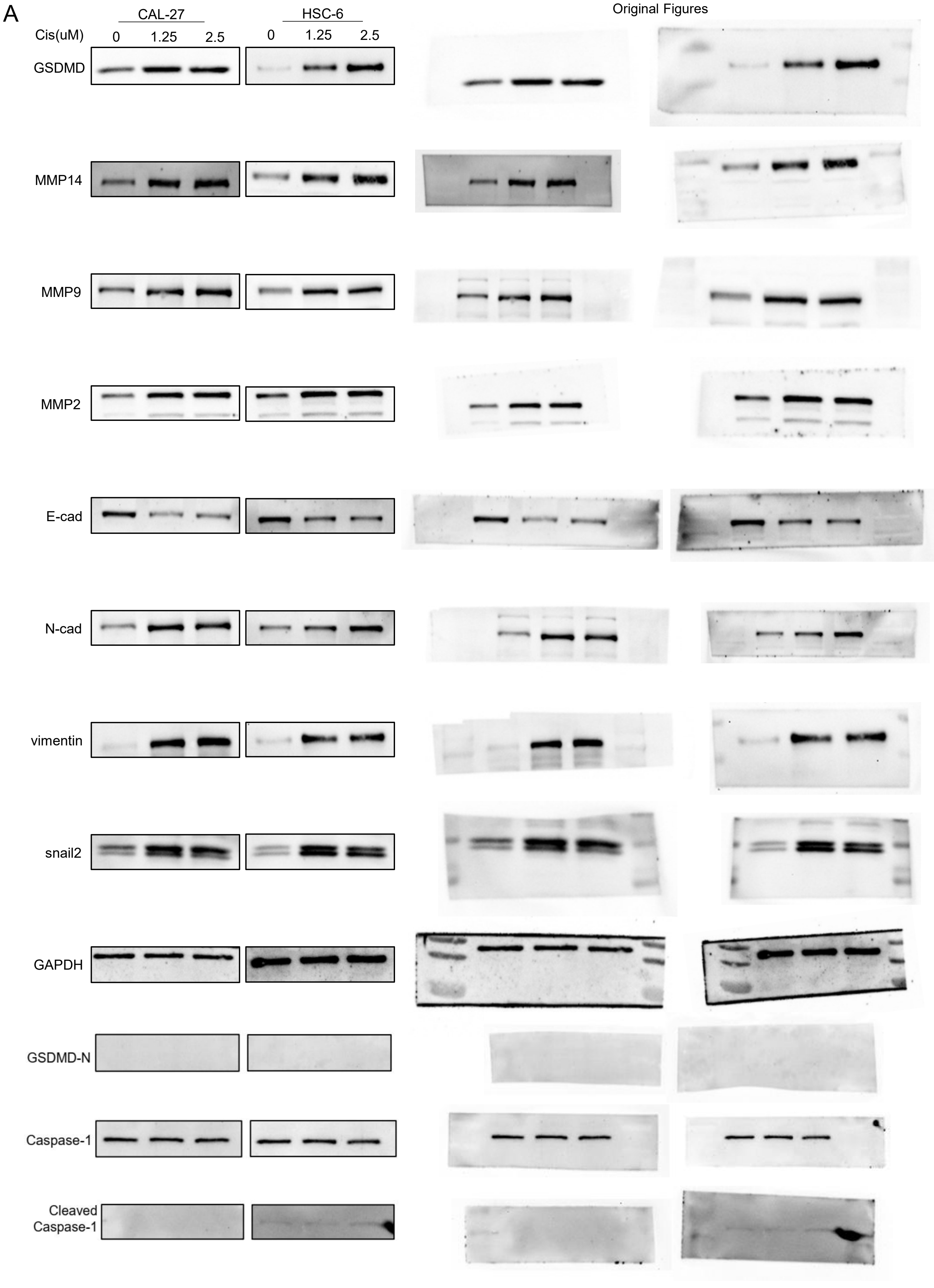
A
CAL-27
HSC-6
Cis(uM)
0
1.25
2.5
0
1.25
2.5
Original Figures
GSDMD
MMP14
MMP9
MMP2
E-cad
N-cad
vimentin
snail2
GAPDH
GSDMD-N
Caspase-1
Cleaved
Caspase-1

Figure s9

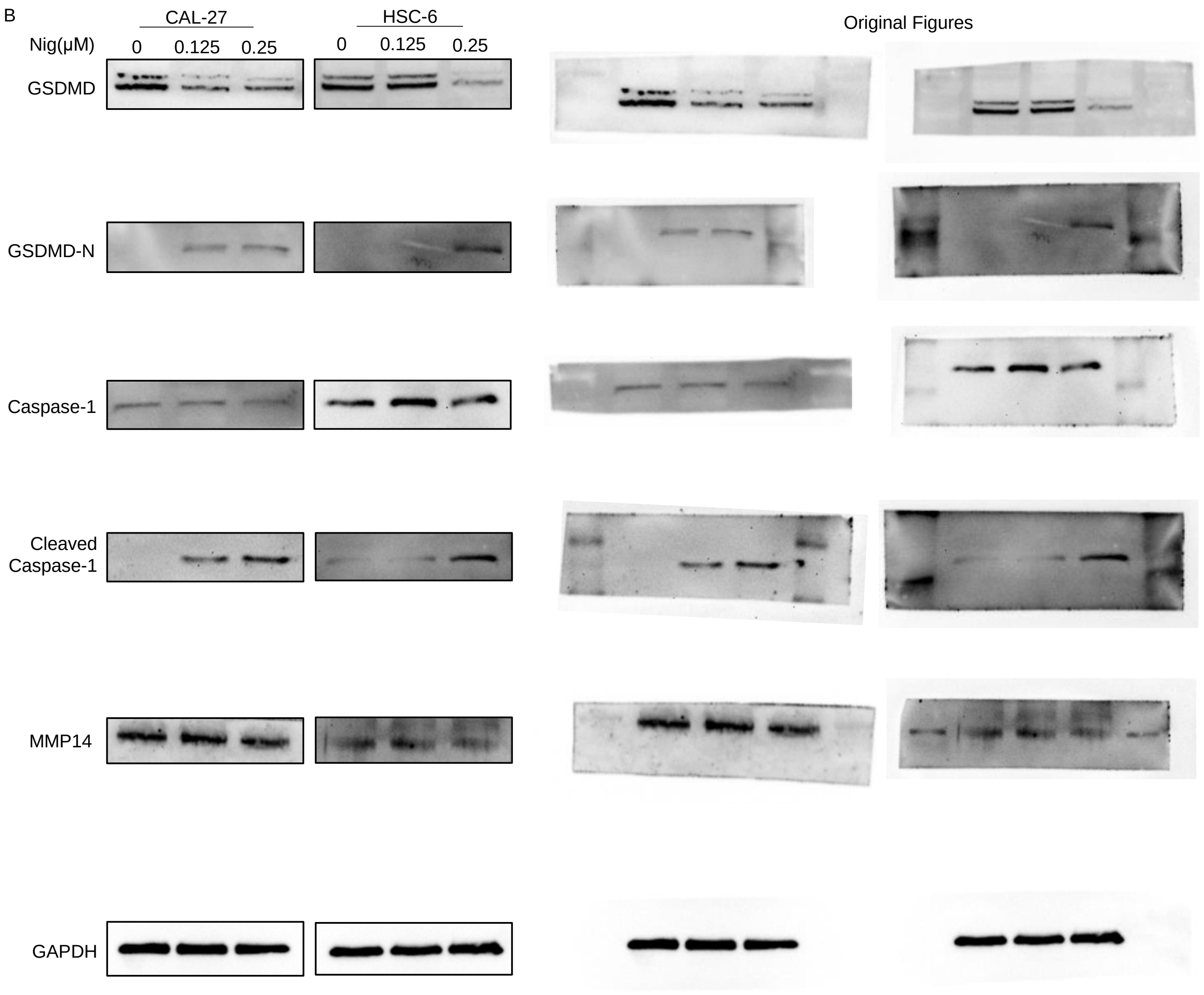
B
CAL-27
HSC-6
Nig(μM)
0
0.125
0.25
0
0.125
0.25
GSDMD
GSDMD-N
Caspase-1
Cleaved
Caspase-1
MMP14
GAPDH
Original Figures

Fig s10.

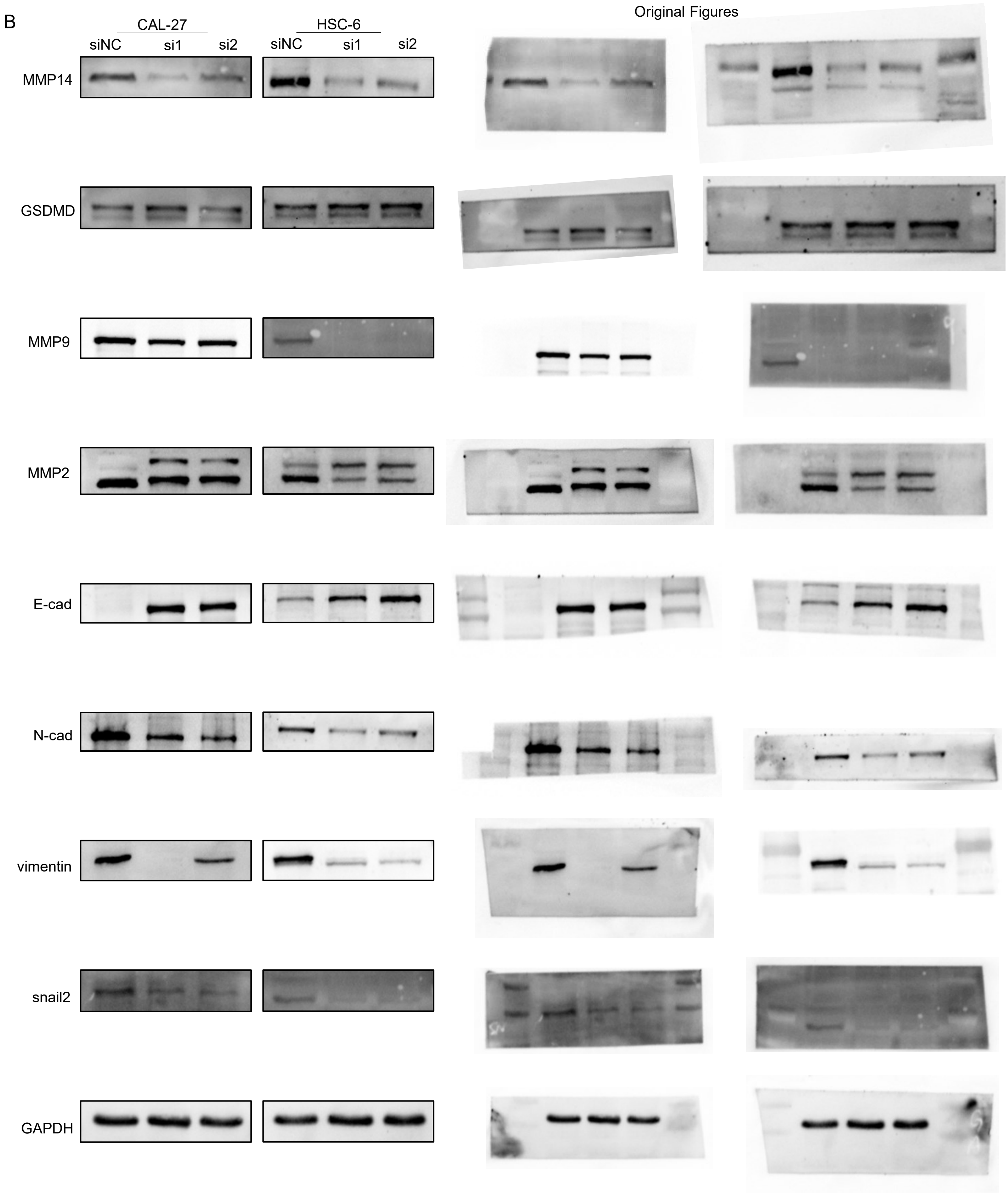
B
CAL-27
HSC-6
siNC
si1
si2
siNC
si1
si2
Original Figures
MMP14
GSDMD
MMP9
MMP2
E-cad
N-cad
vimentin
snail2
GAPDH
