# Supplementary material for: Neoadjuvant Chemotherapy With Cisplatin Up‐Regulates GSDMD to Enhance Oral Squamous Cell Carcinoma Metastasis Through MMP14‐Mediated EMT Activation
Source: Adv Sci (Weinh). 2025 Apr 3;12(25):2501149. doi: 10.1002/advs.202501149 (PMC12224931; doi:10.1002/advs.202501149)

ADVANCED SCIENCE
Open Access

Supporting Information

for *Adv. Sci.*, DOI 10.1002/advs.202501149

Neoadjuvant Chemotherapy With Cisplatin Up-Regulates GSDMD to Enhance Oral Squamous Cell Carcinoma Metastasis Through MMP14-Mediated EMT Activation

Zixian Huang, Qiming Jiang, Qianyu Zhang, Nan Lu, Xi Rui, Rui Chen, Yan Wang, Yuepeng Wang, Xiaoding Xu and Zhiquan Huang**

Figure 1A

Figure 1A CAL-27 Cis 0 μM

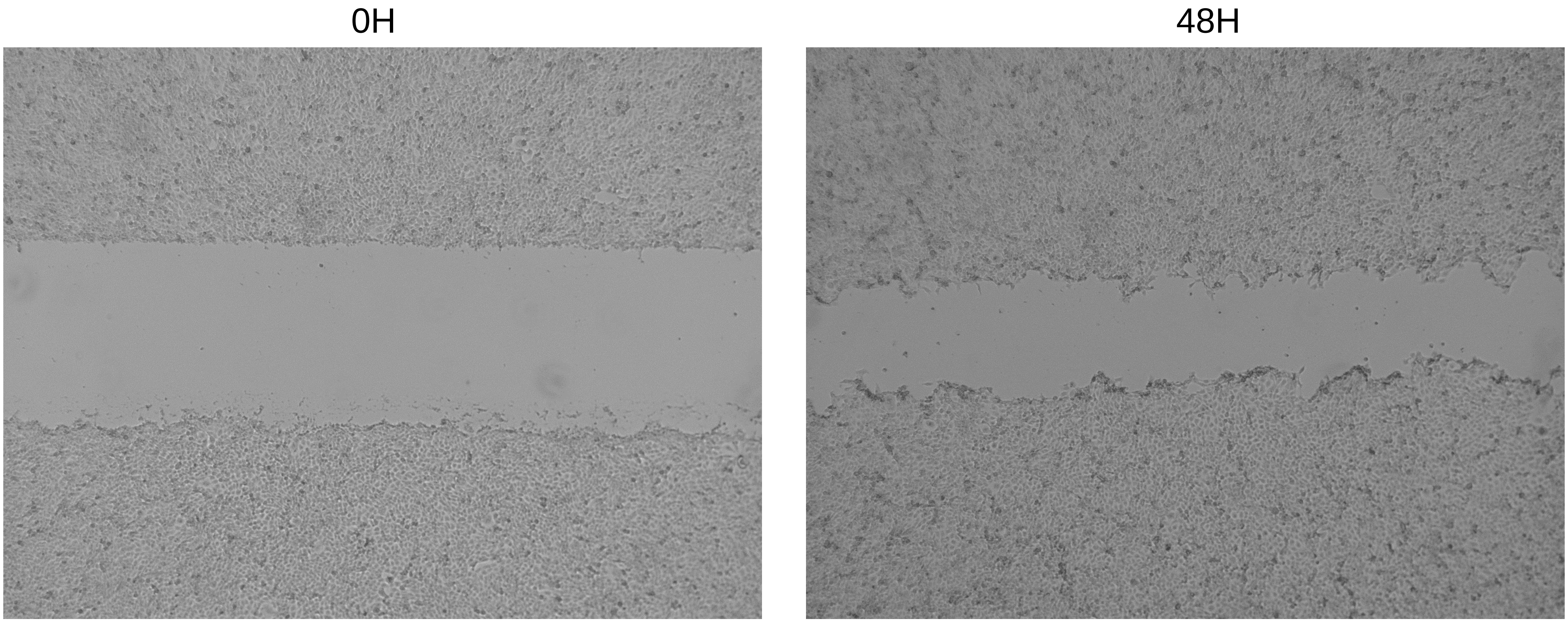

Figure 1A CAL-27 Cis 1.25 μM

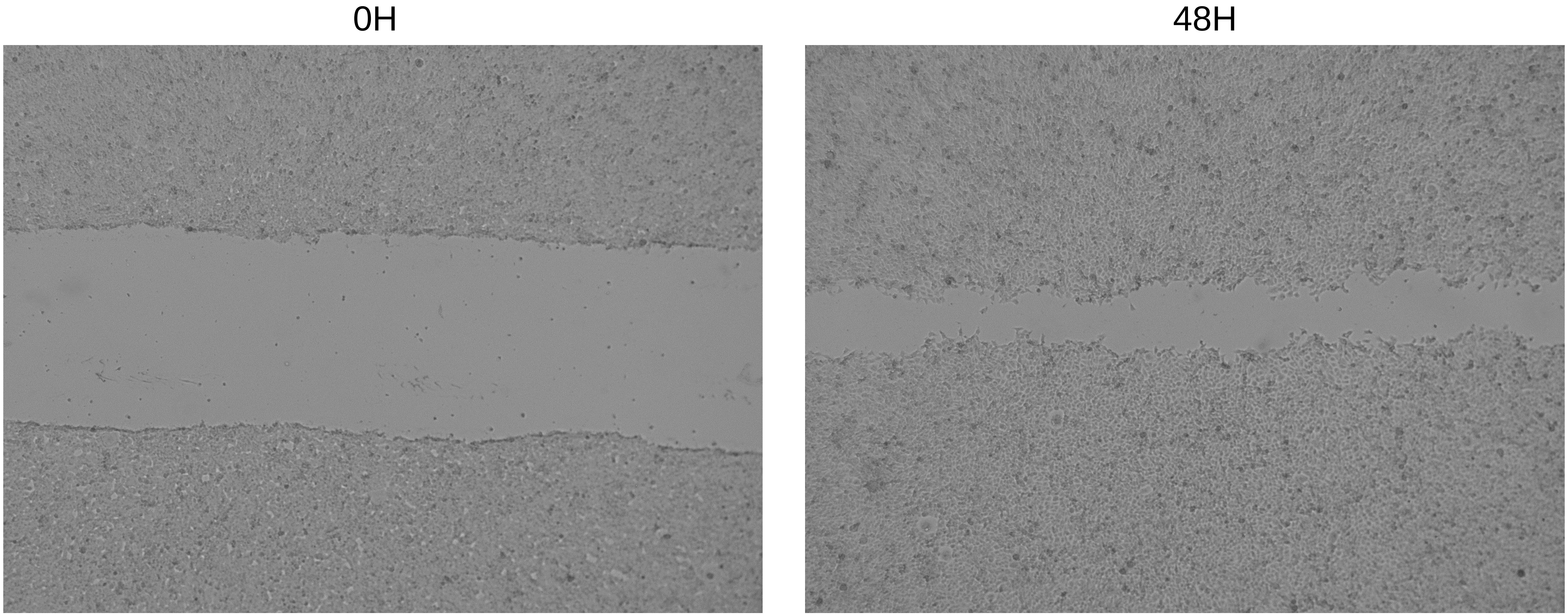

Figure 1A CAL-27 Cis 2.5 μM

0H 48H

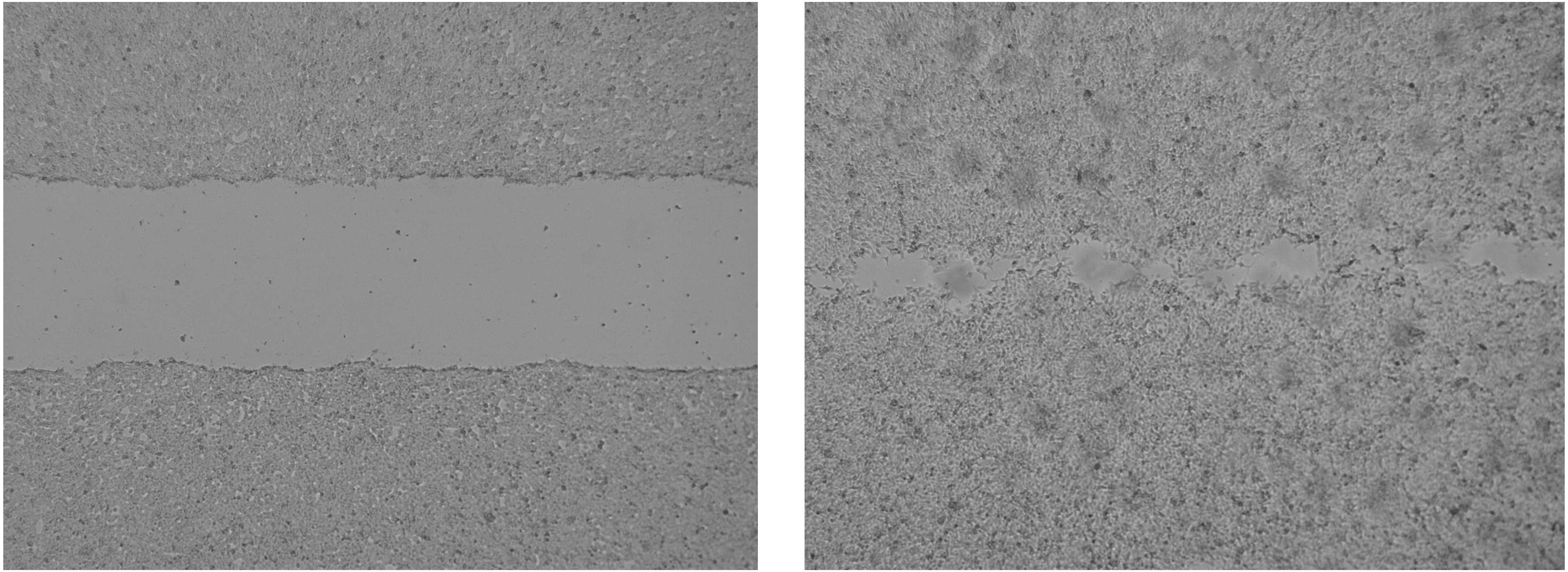

Figure 1A CAL-27 Cis 5 µM

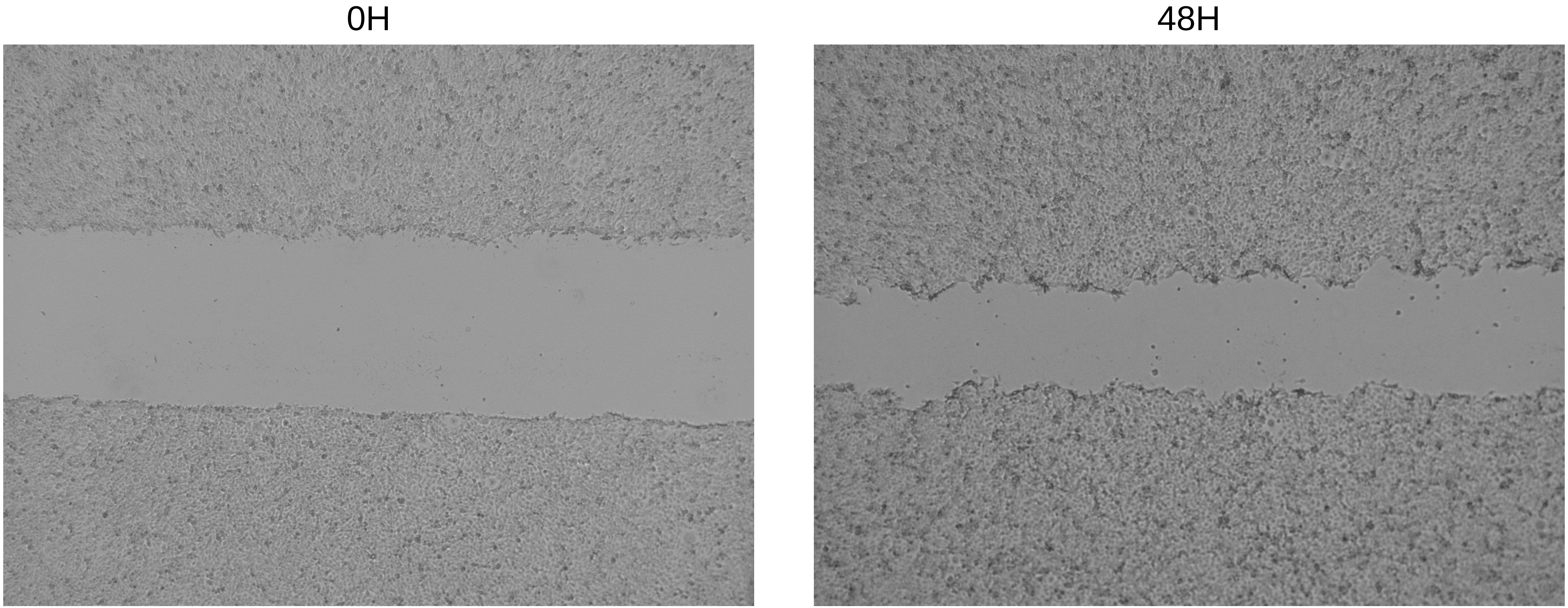

Figure 1A CAL-27 Cis 10 μM

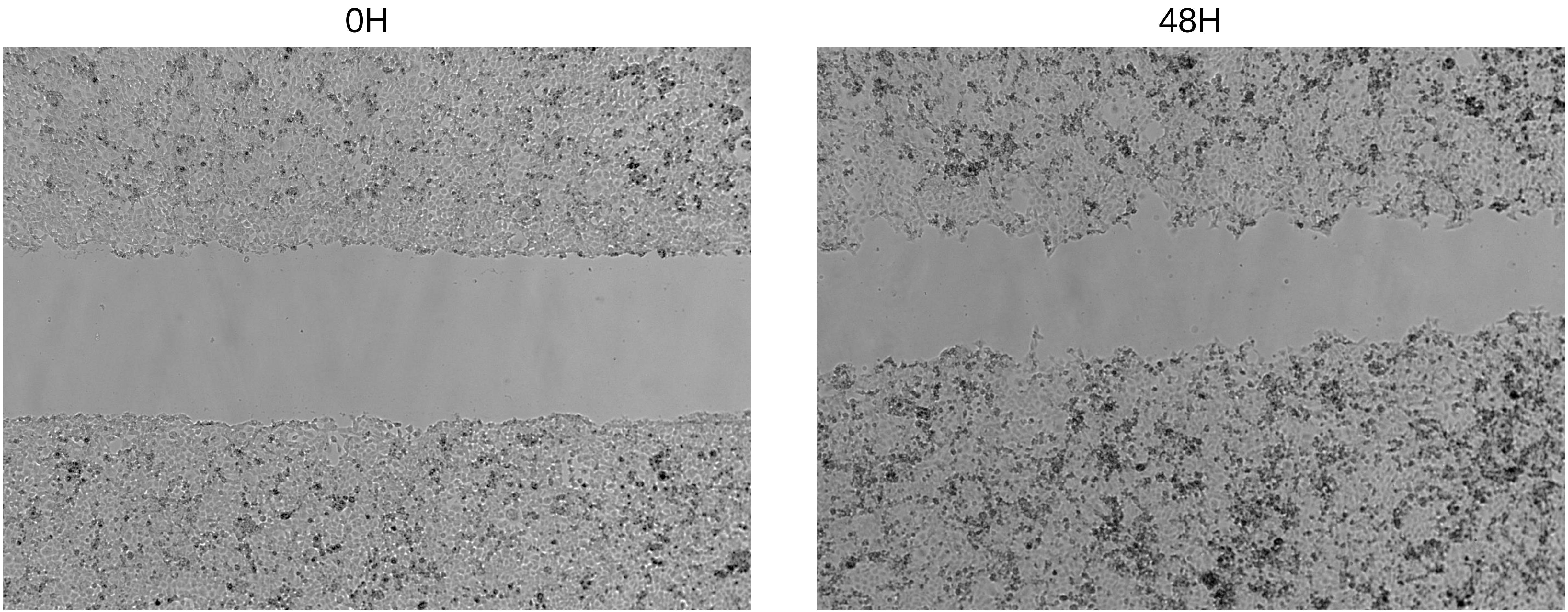

Figure 1A HSC-6 Cis 0 μM

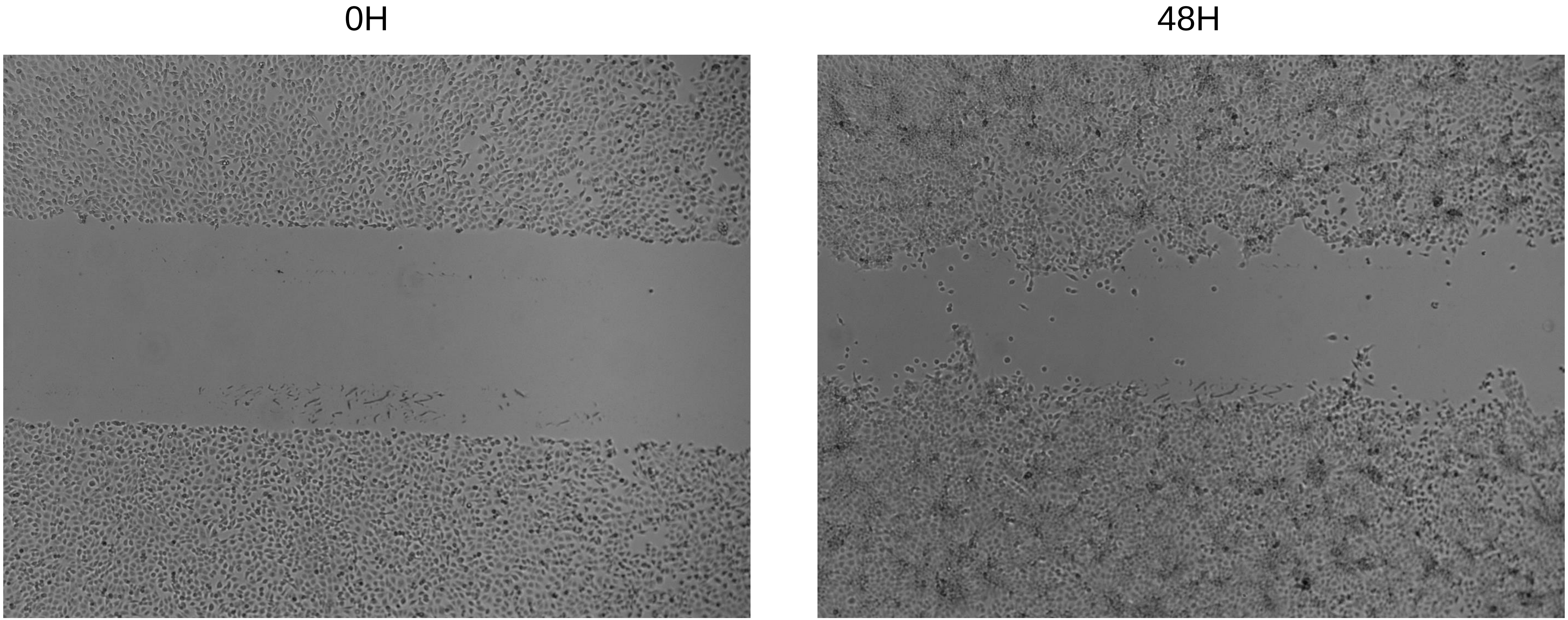

Figure 1A HSC-6 Cis 1.25 μM

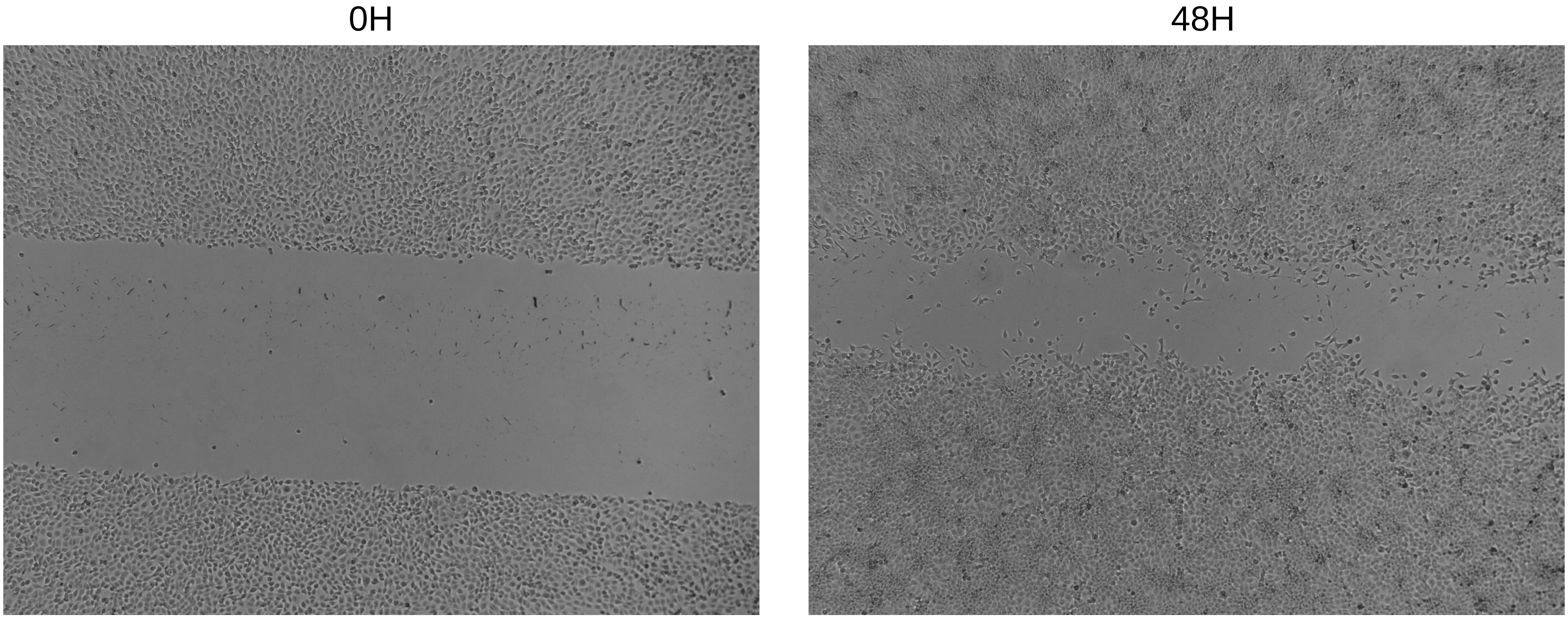

Figure 1A HSC-6 Cis 2.5 μM

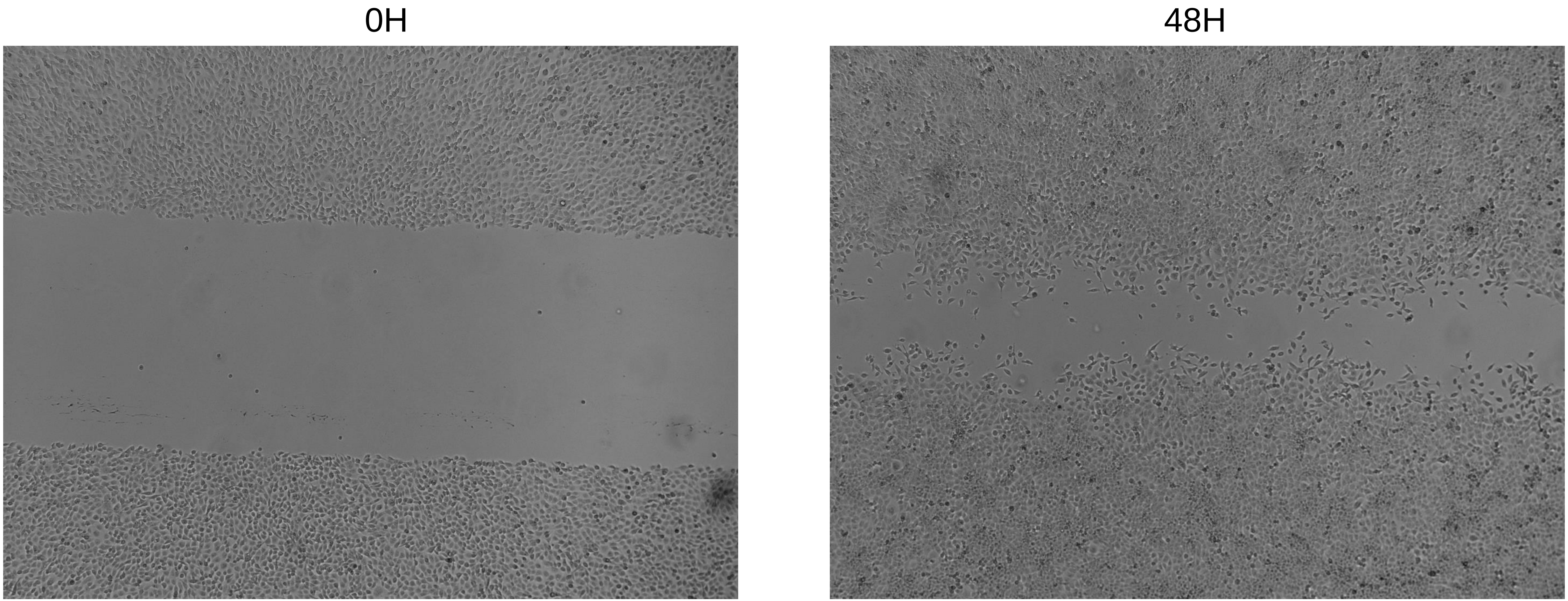

Figure 1A CAL-27 Cis 5 μM

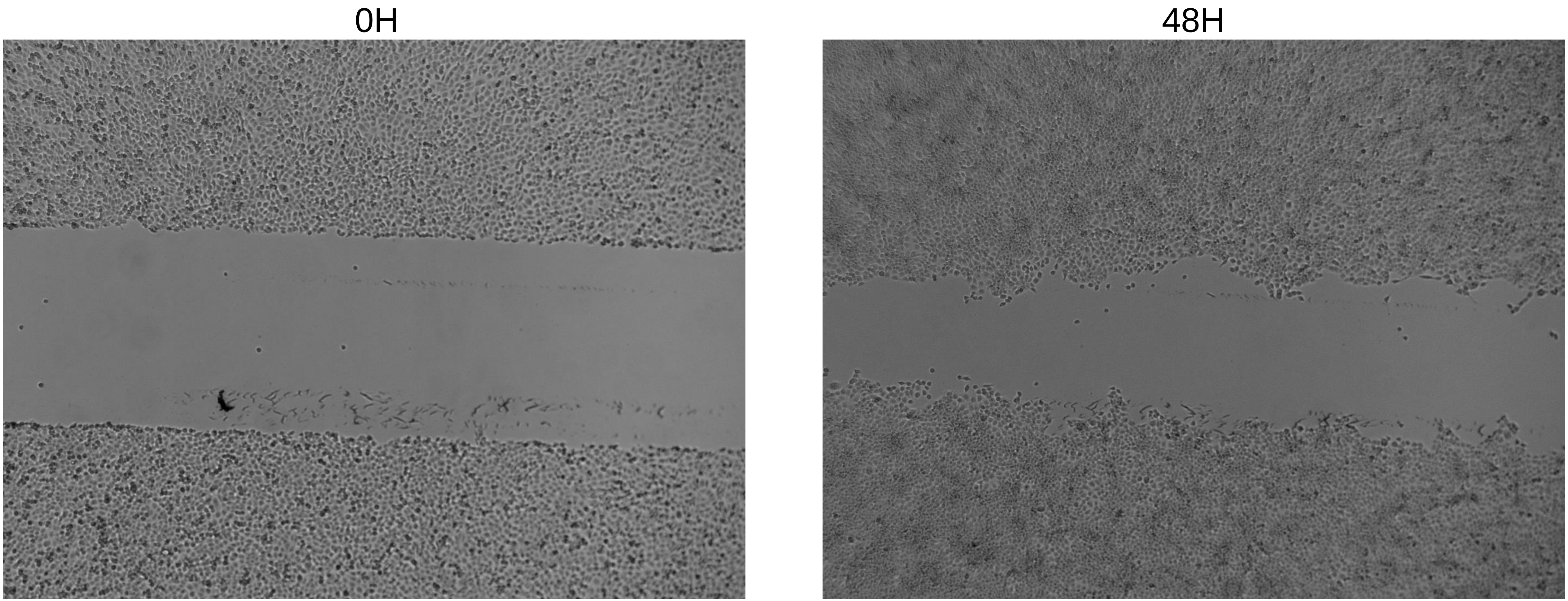

Figure 1A HSC-6 Cis 10 μM

0H 48H

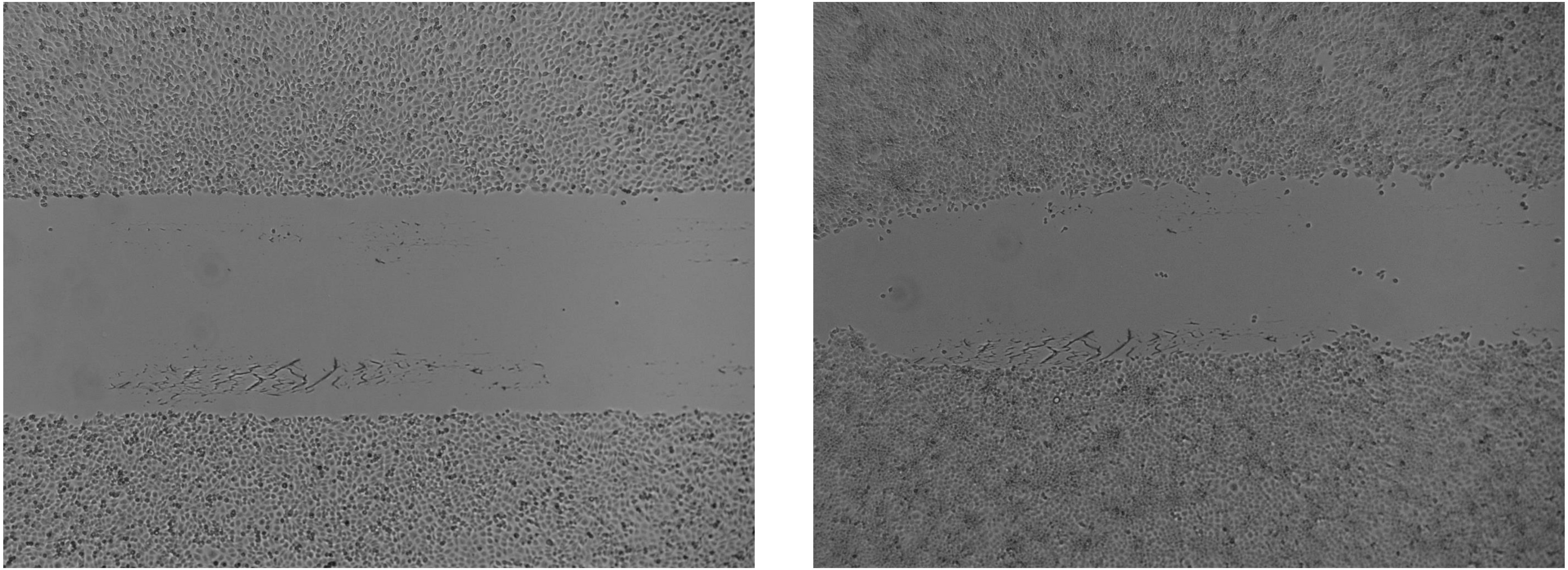

Figure s2B

Figure s2B CAL-27 Doce 0 μM

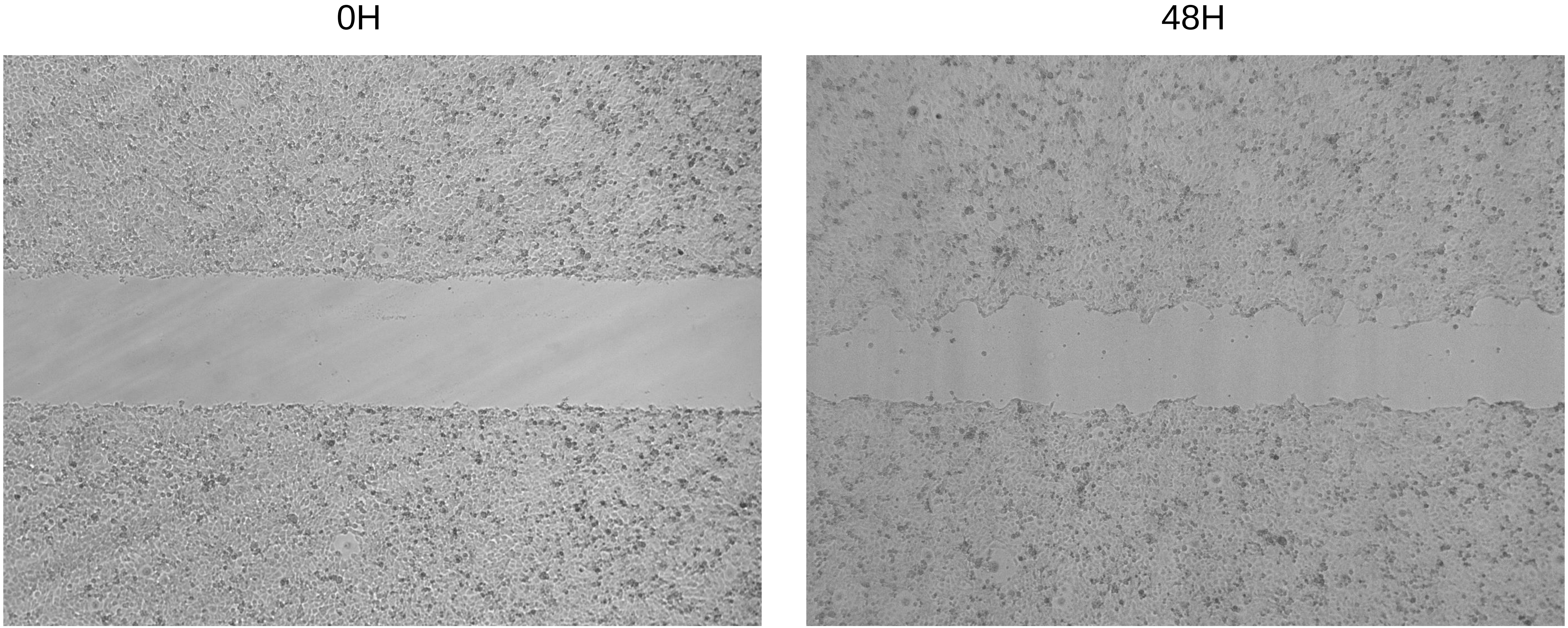

Figure s2B CAL-27 Doce 1.25 μM

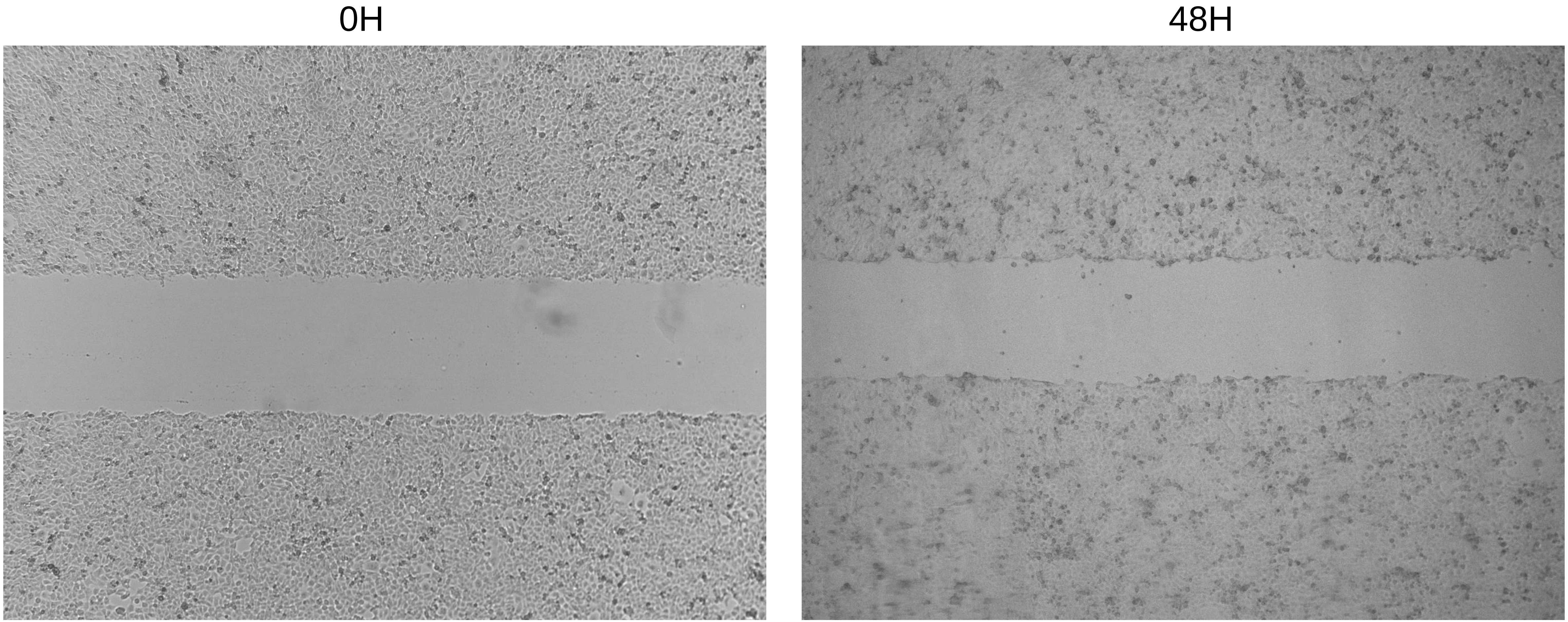

Figure s2B CAL-27 Doce 2.5 μM

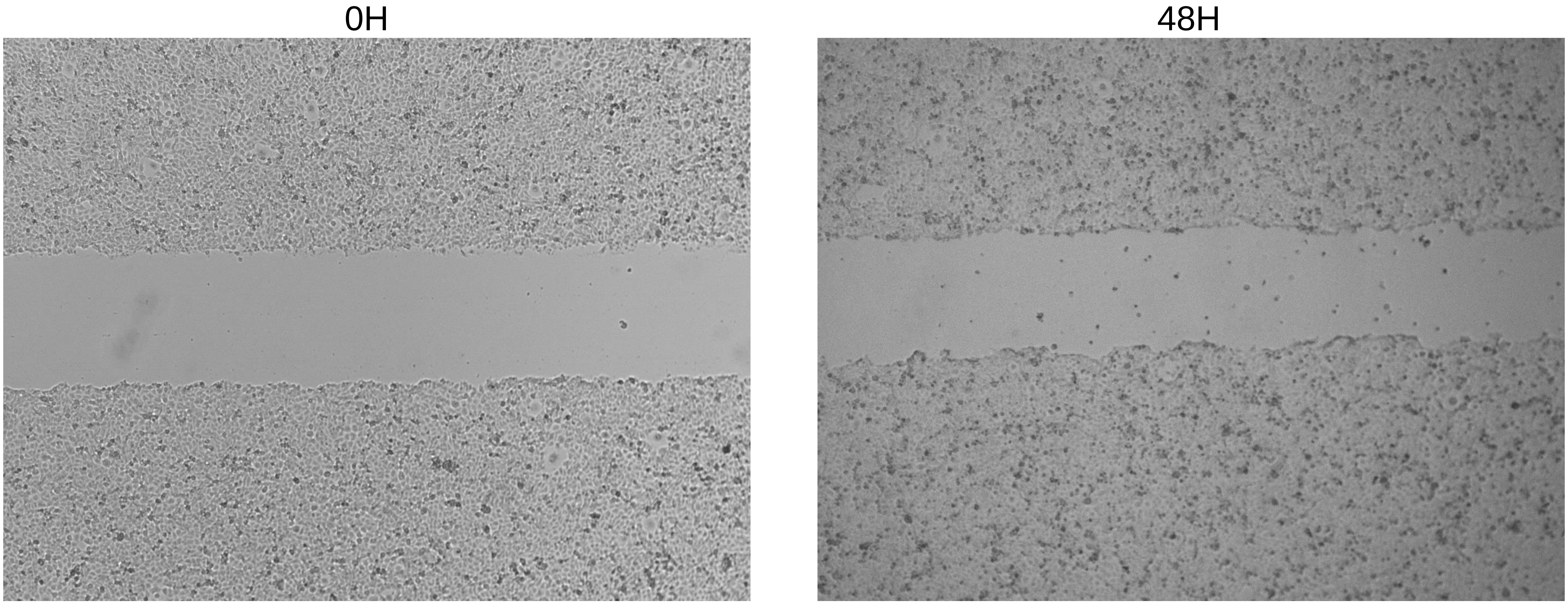

Figure s2B HSC-6 Doce 0μM

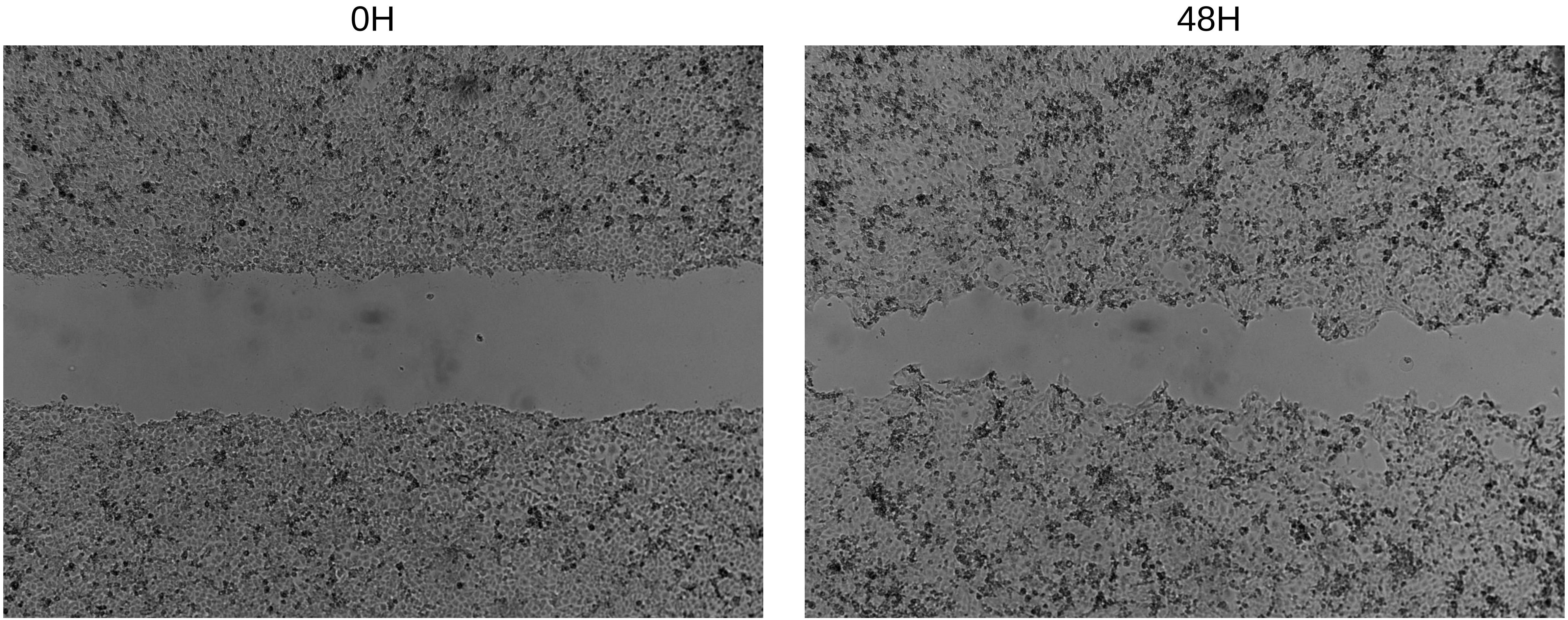

Figure s2B HSC-6 Doce 1.25μM

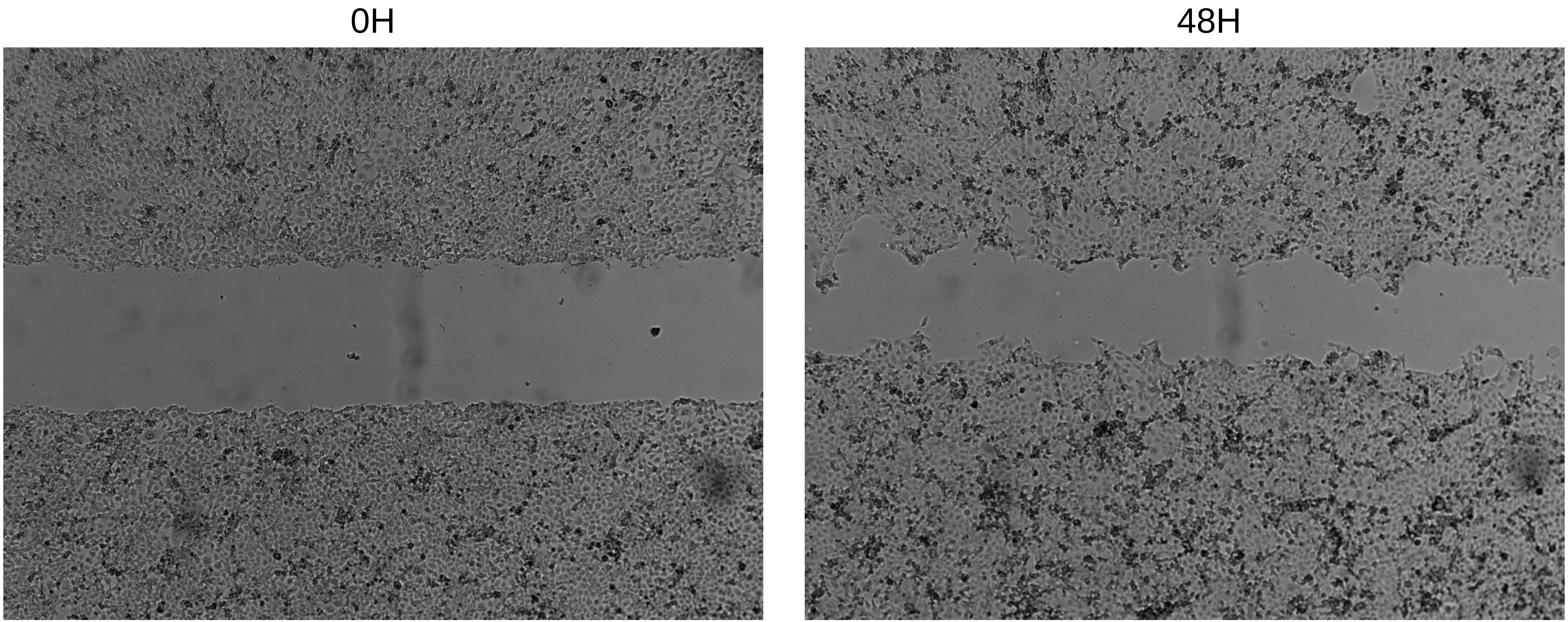

Figure s2B HSC-6 Doce 2.5μM

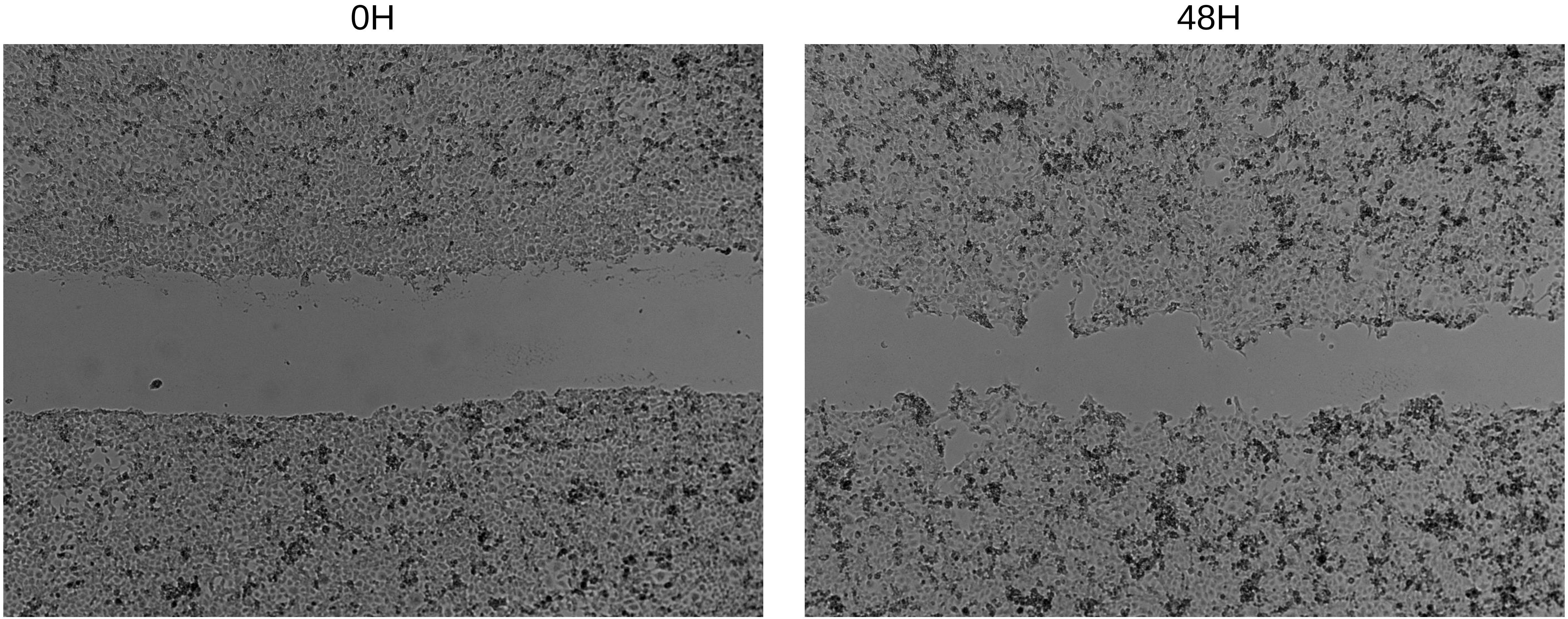

Figure s2D

Figure s2D CAL-27 5-FU 0 μM

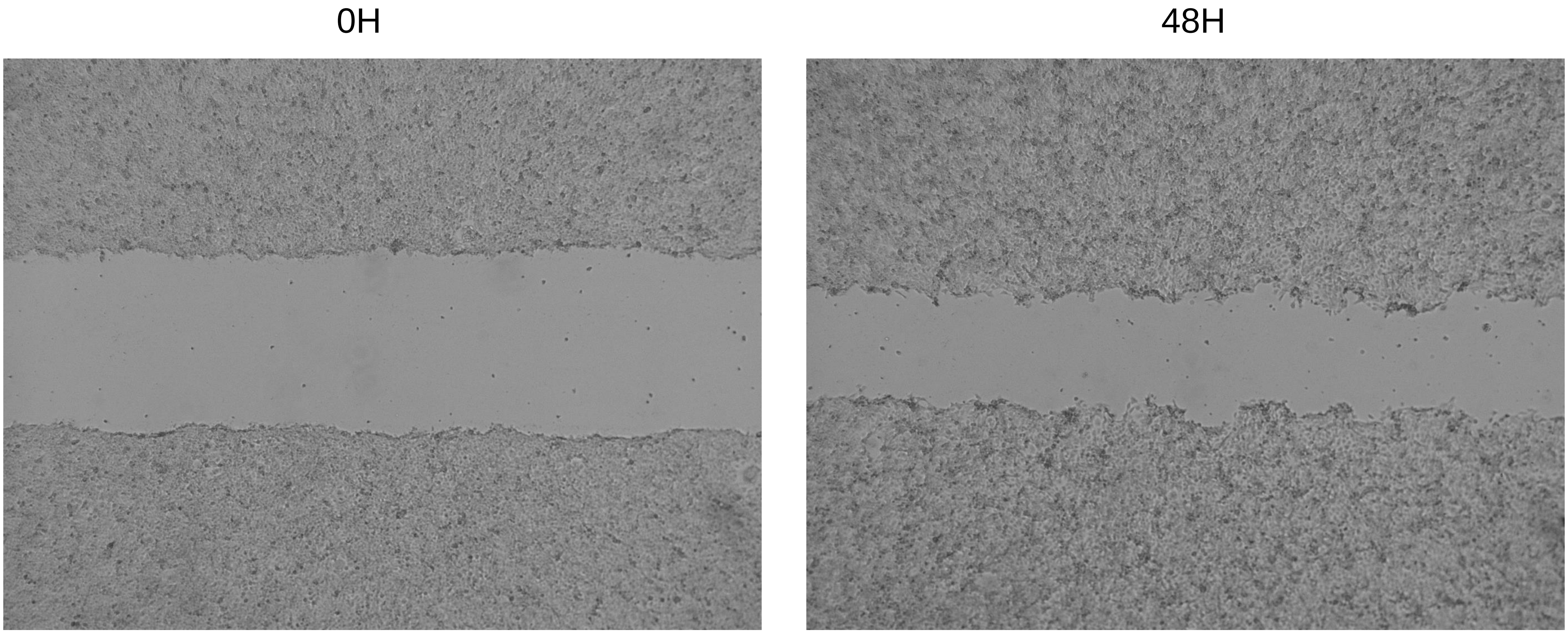

Figure s2D CAL-27 5-FU 100 μM

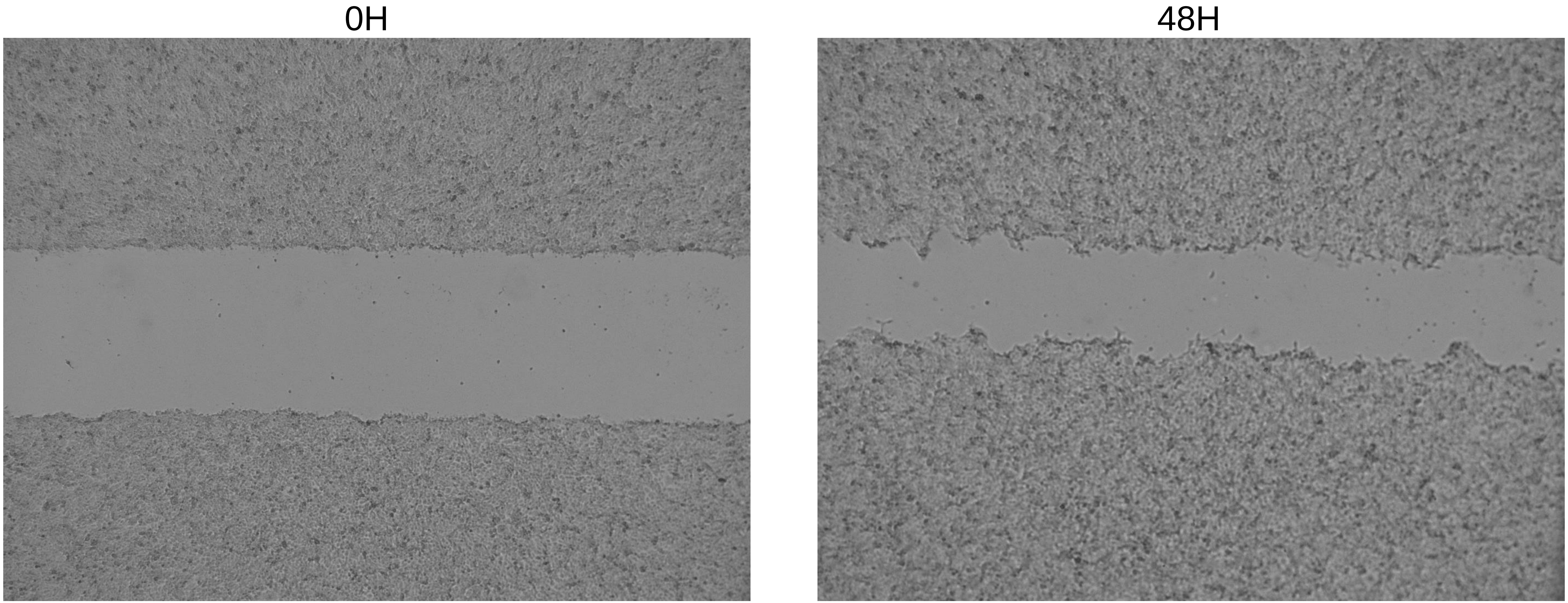

Figure s2D CAL-27 5-FU 200 μM

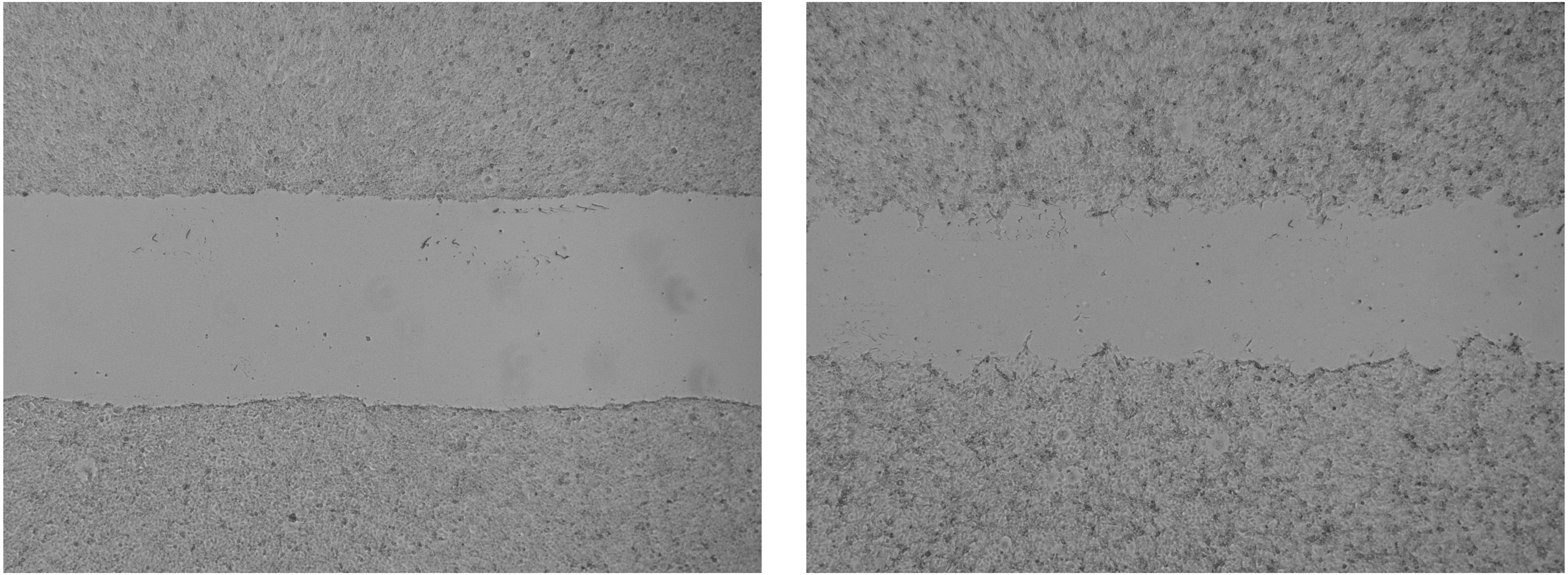

0H

48H

Figure s2D HSC-6 5-FU 0 μM

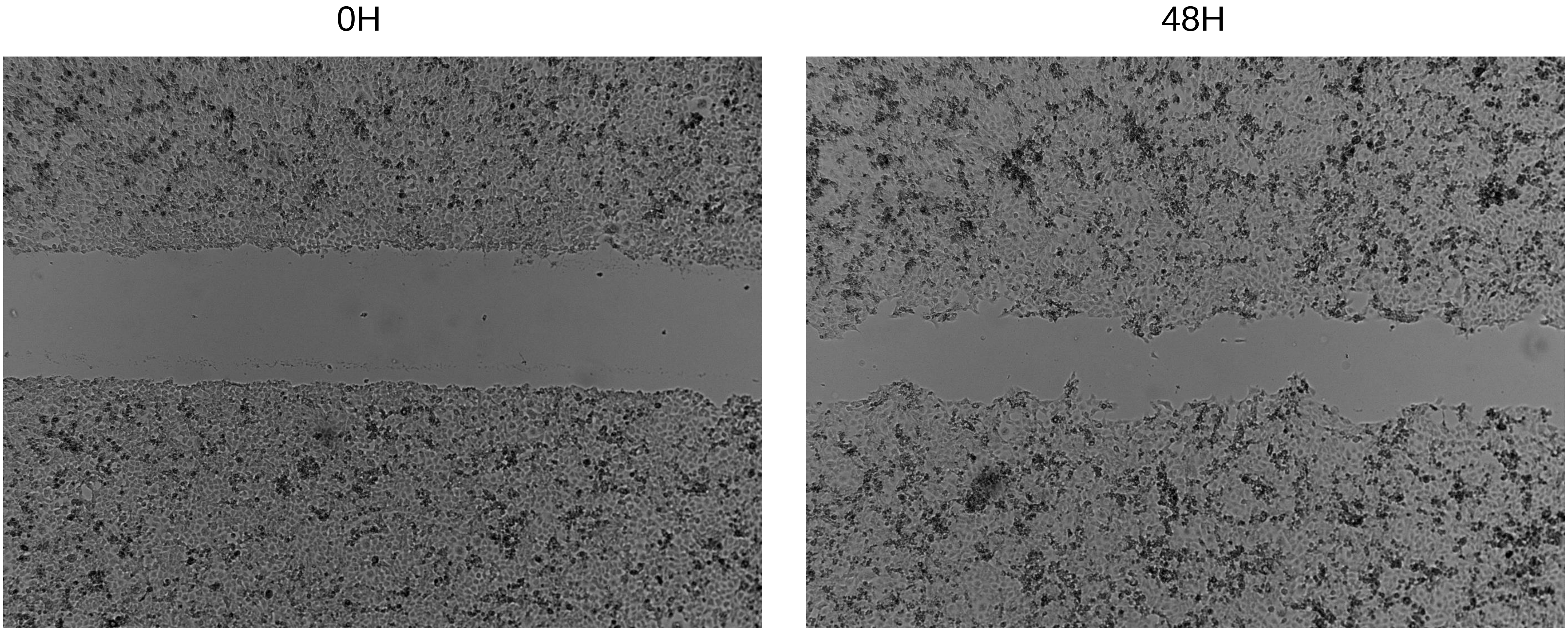

Figure s2D HSC-6 5-FU 100 μM

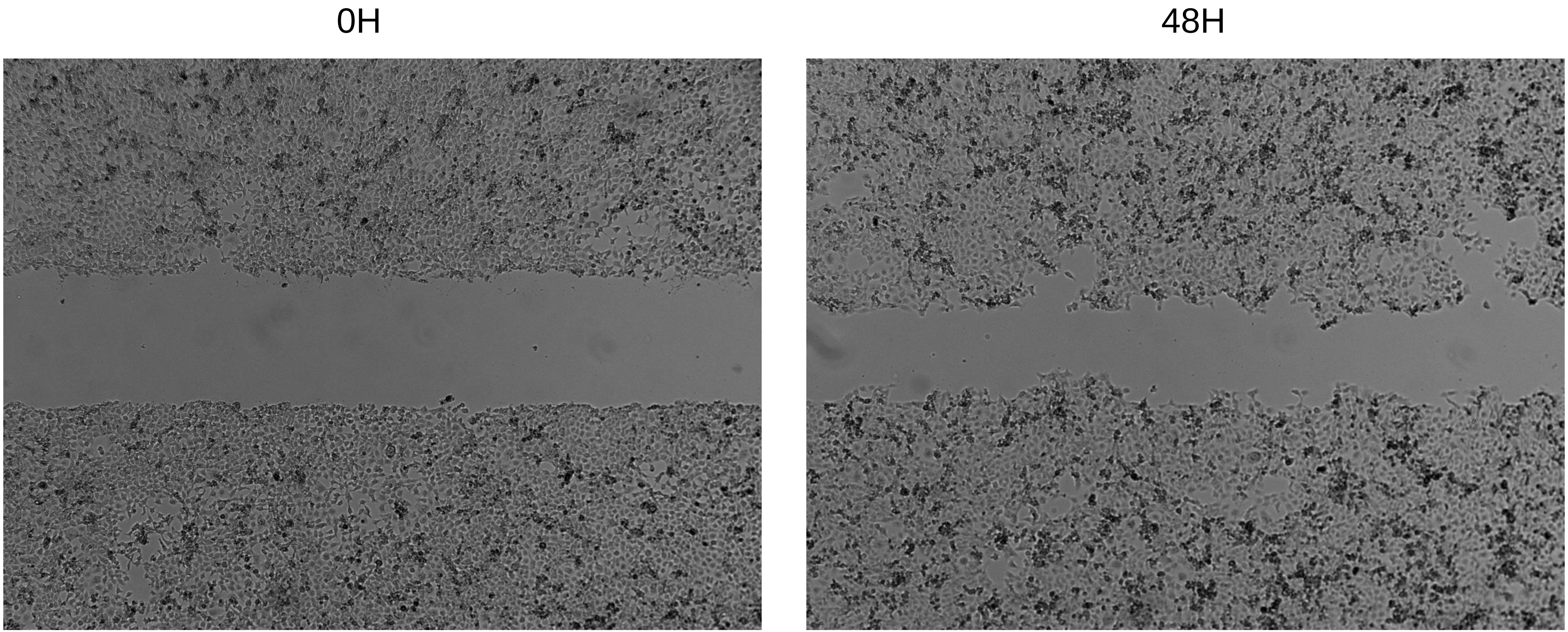

Figure s2D HSC-6 5-FU 200 μM

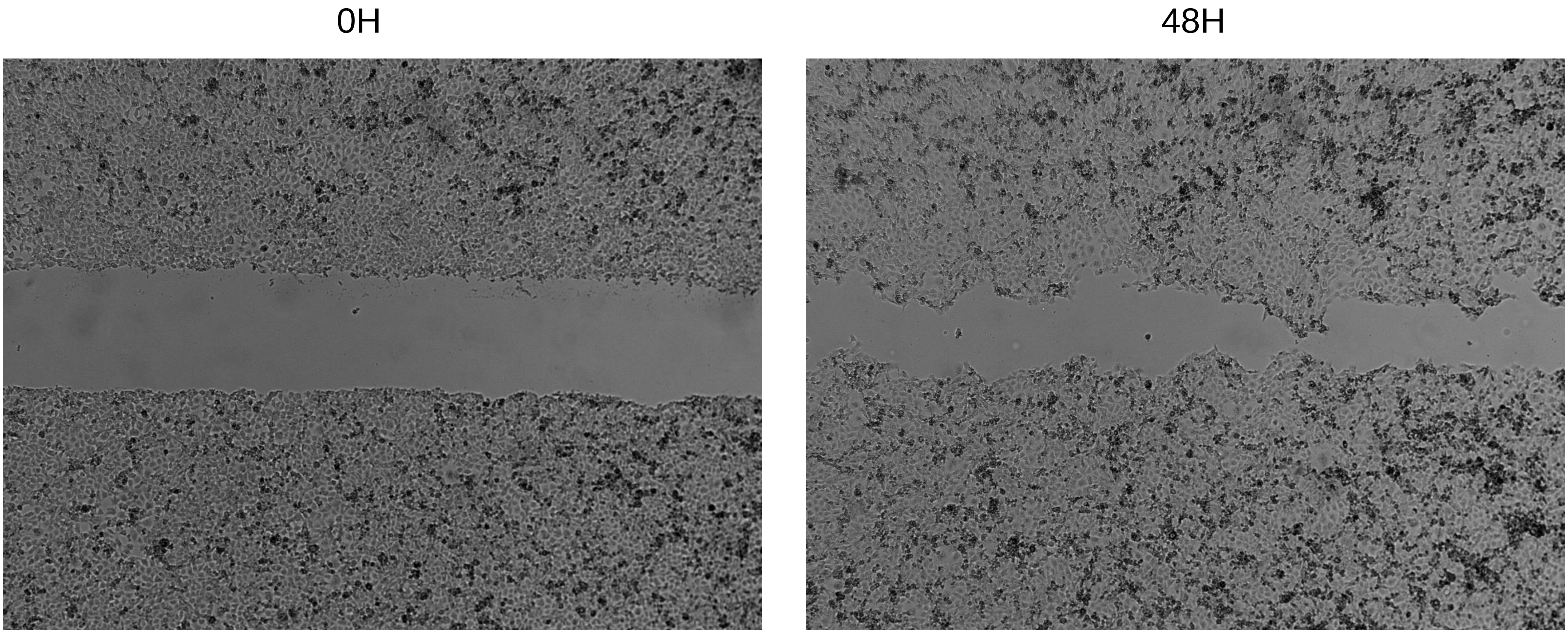

Figure s3E

Figure s3E CAL-27 siNC

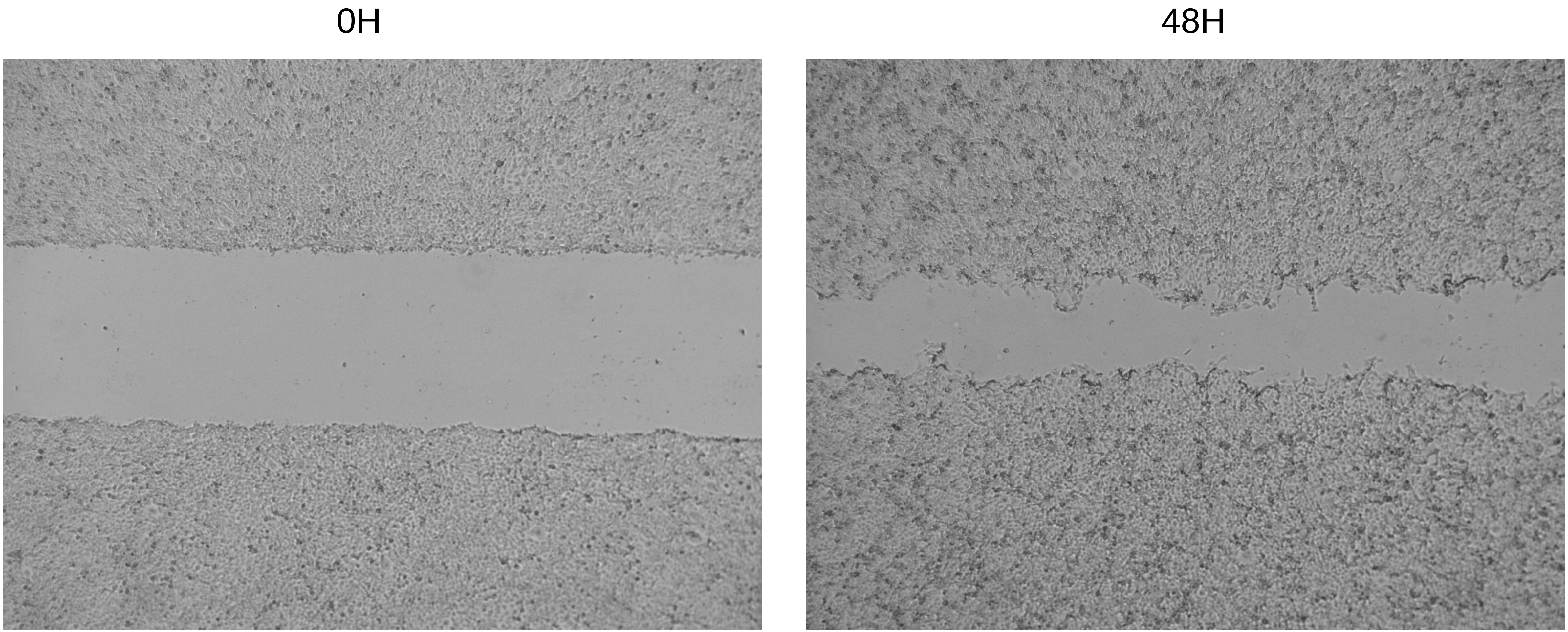

Figure s3E CAL-27 siCCND1-1

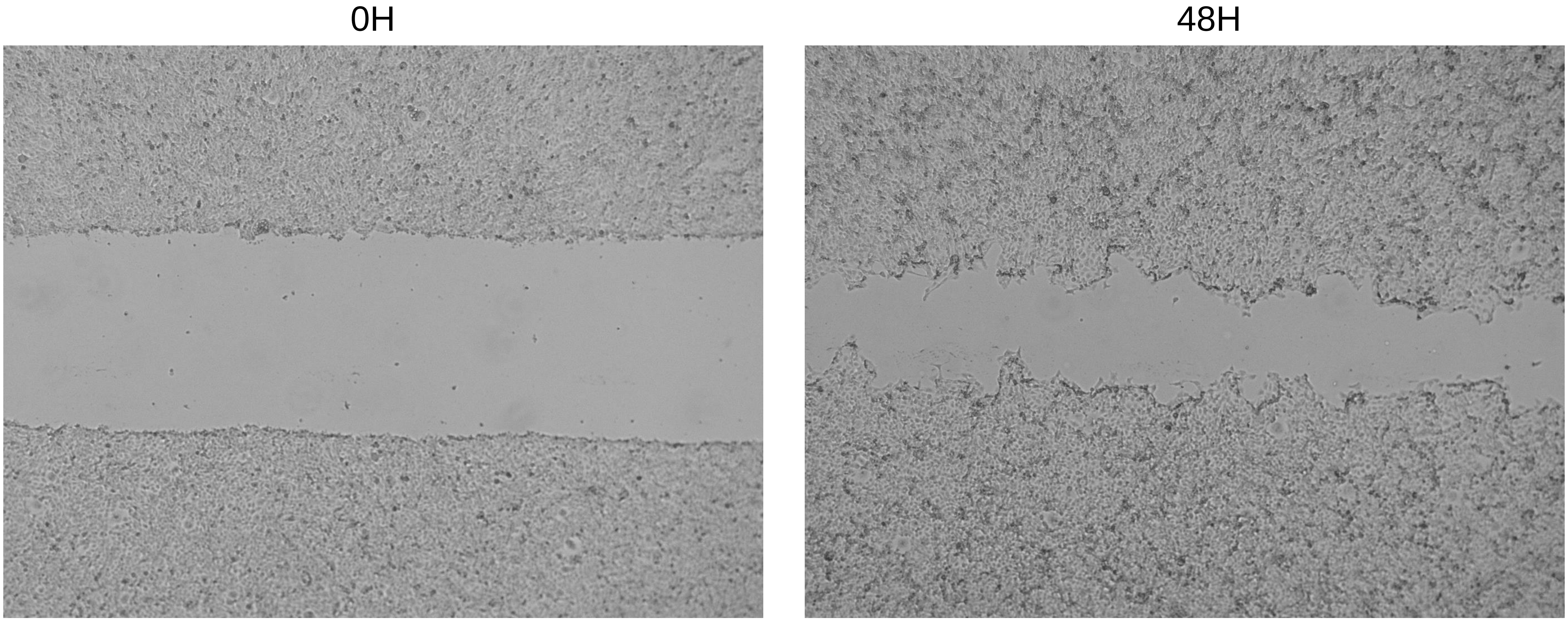

Figure s3E CAL-27 siCCND1-2

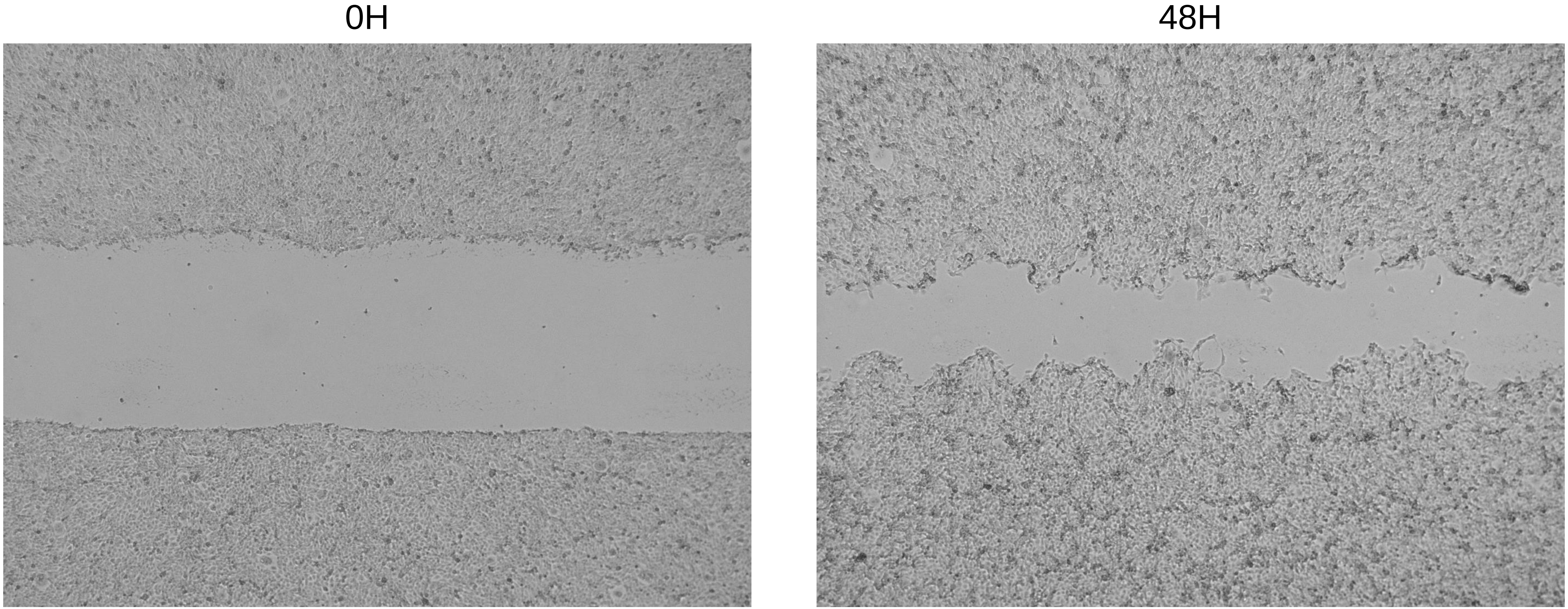

Figure s3E HSC-6 siNC

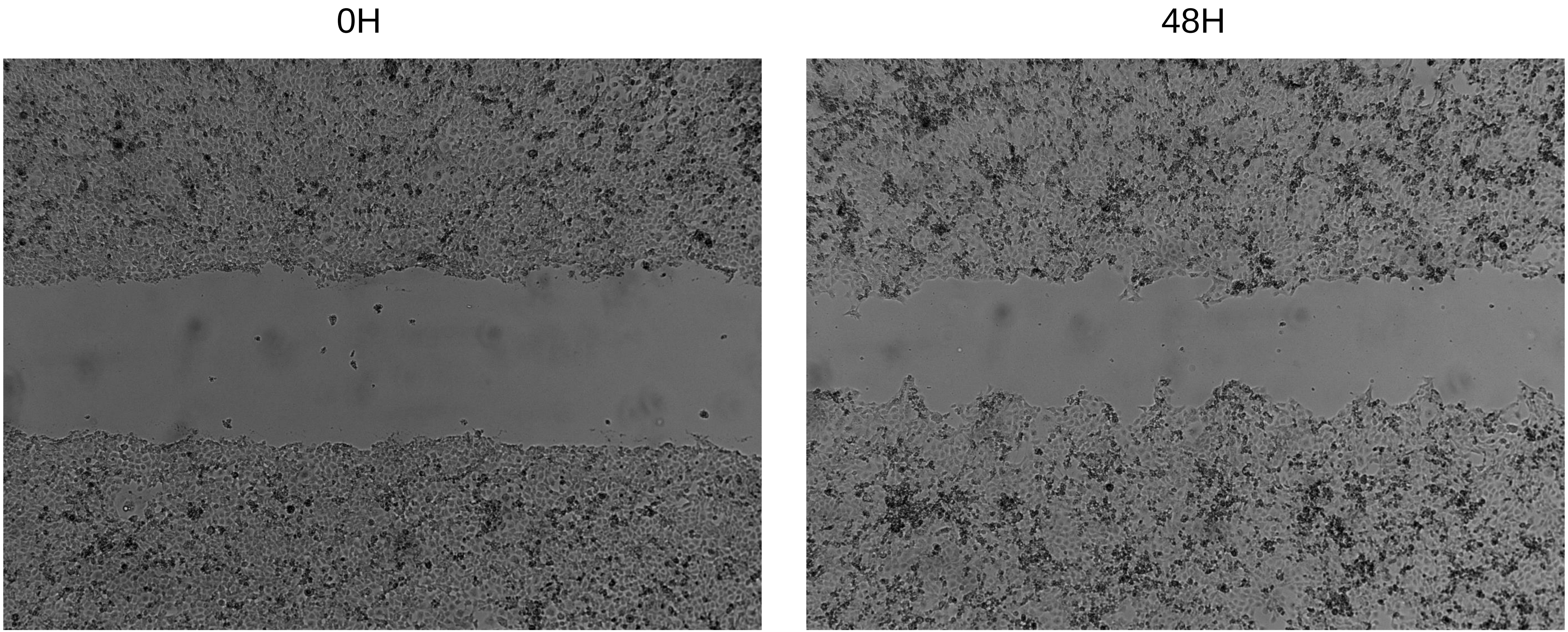

Figure s3E HSC-6 siCCND1-1

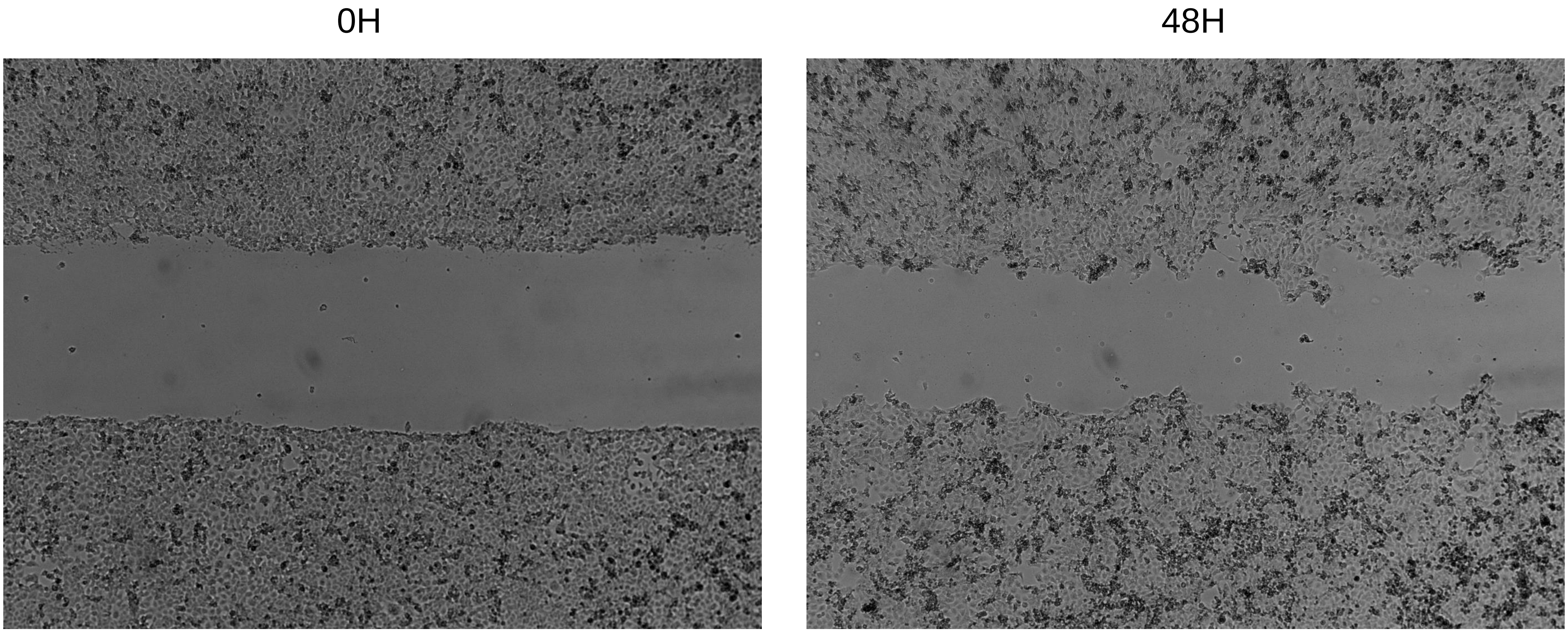

Figure s3E HSC-6 siCCND1-2

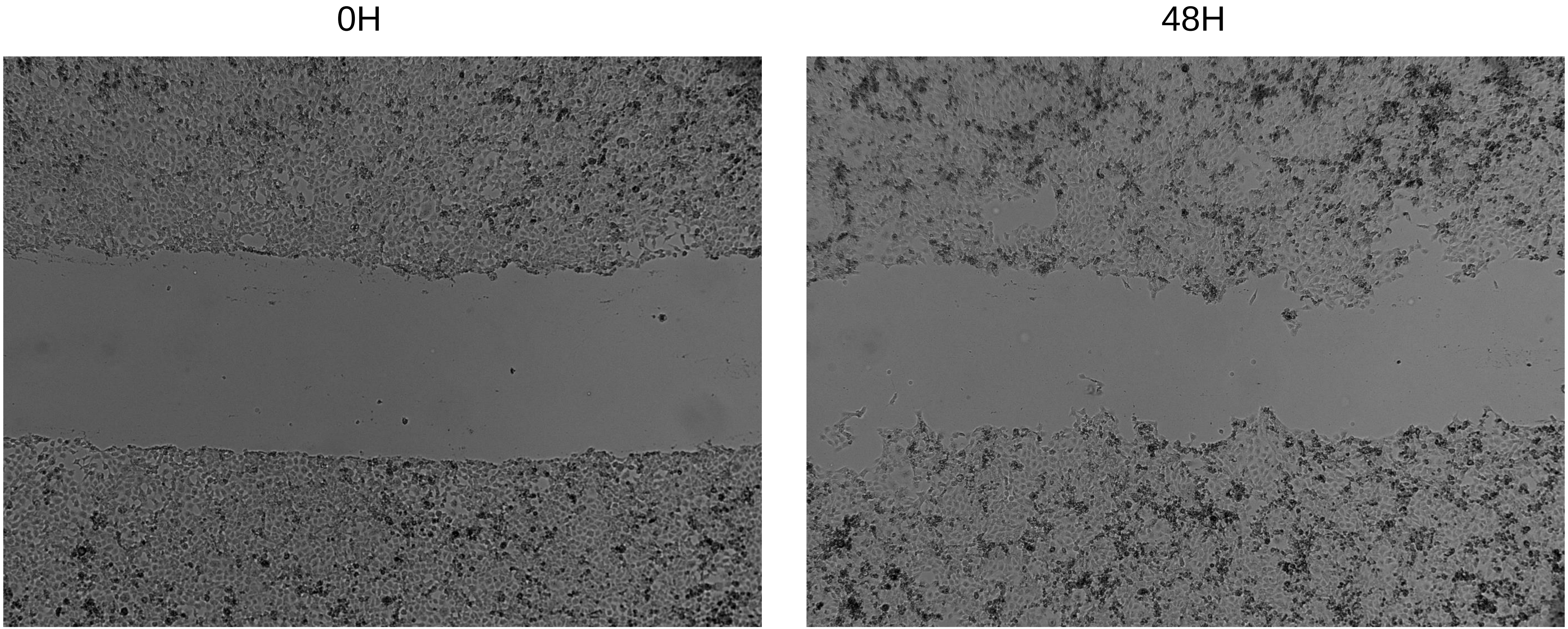

Figure s4E

Figure s4E CAL-27 siNC

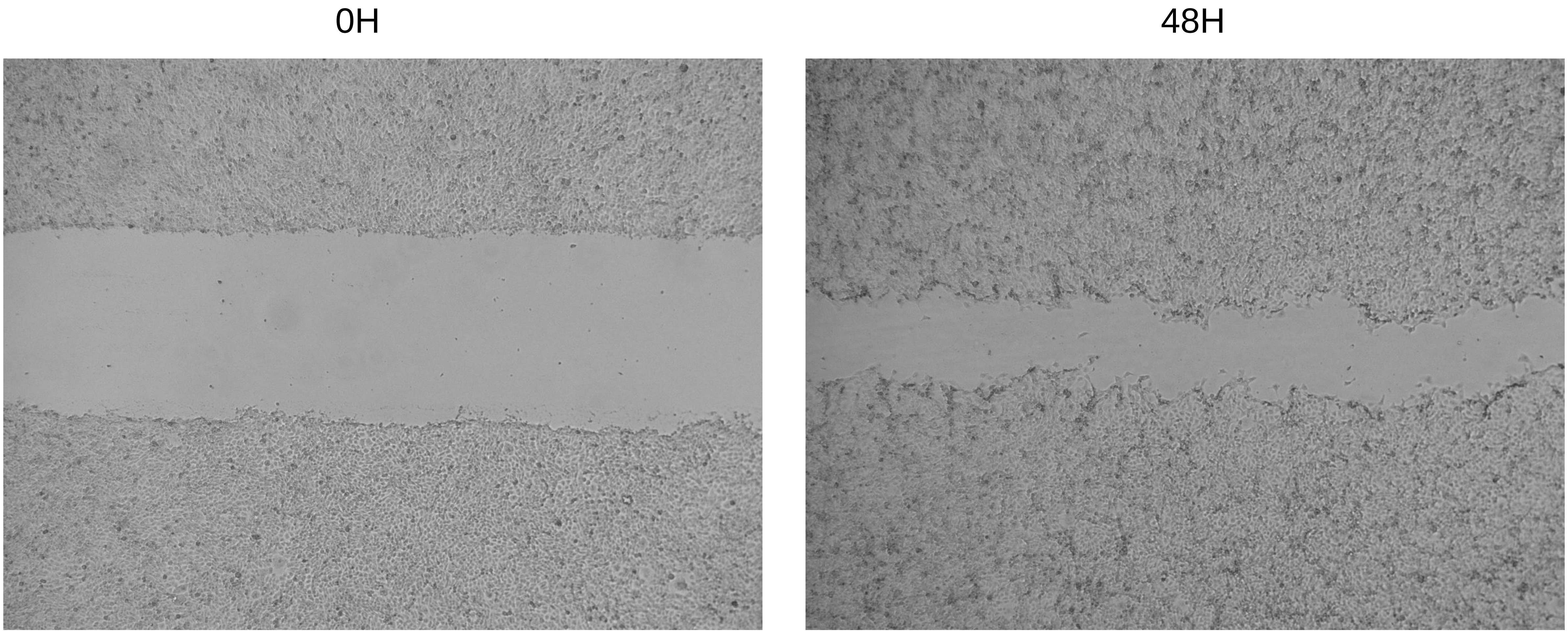

Figure s4E CAL-27 siBATF2-1

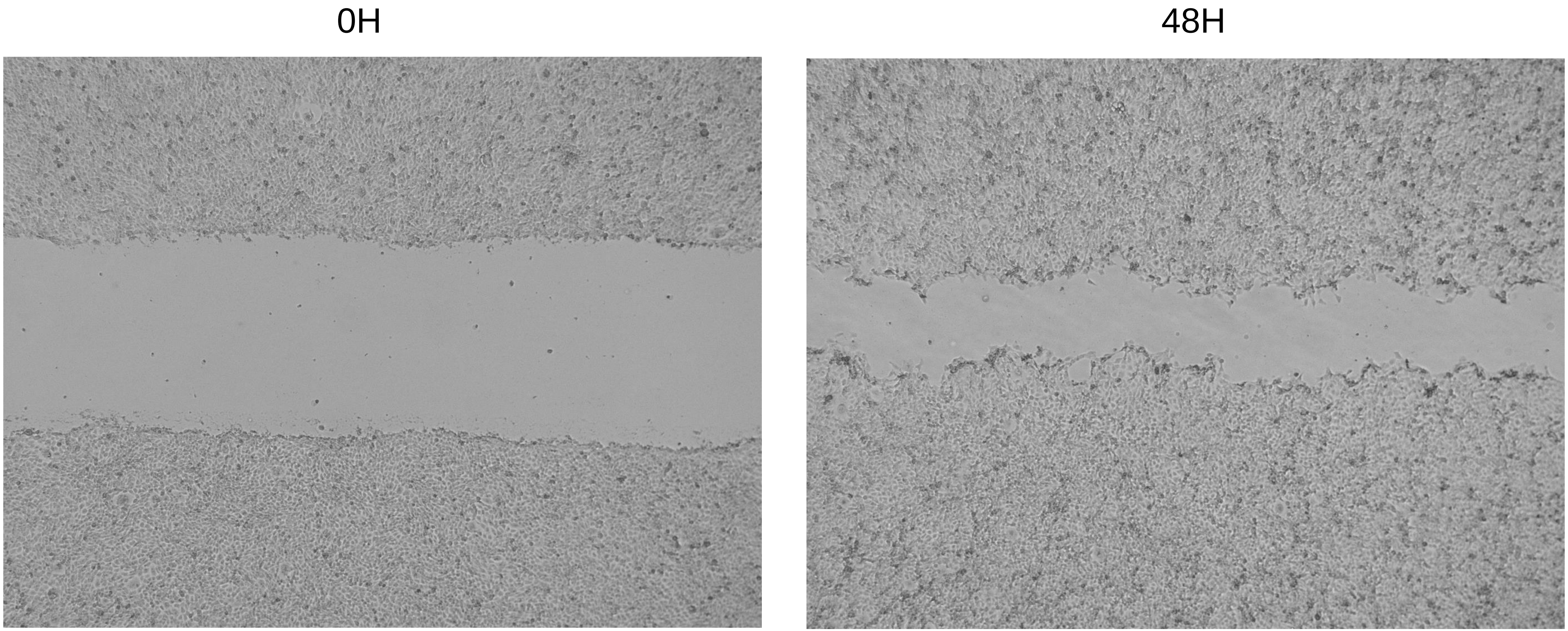

Figure s4E CAL-27 siBATF2-2

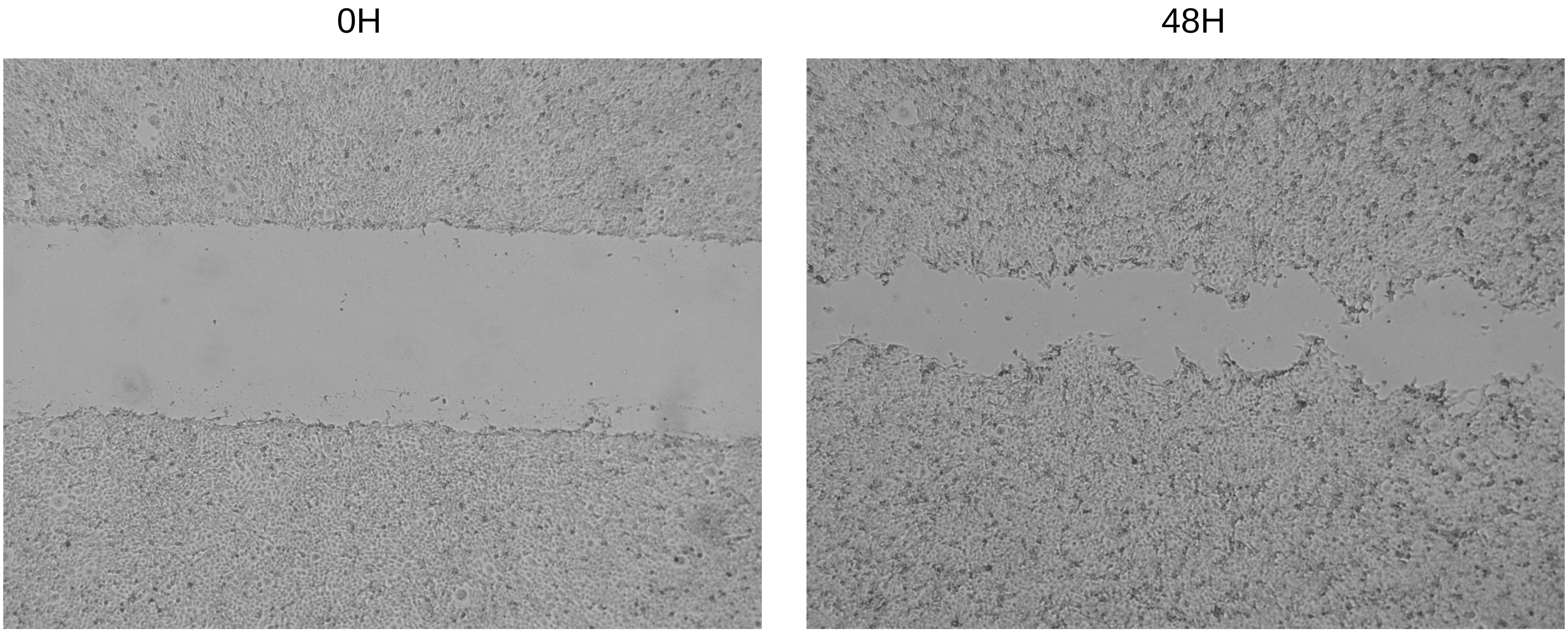

Figure s4E HSC-6 siNC

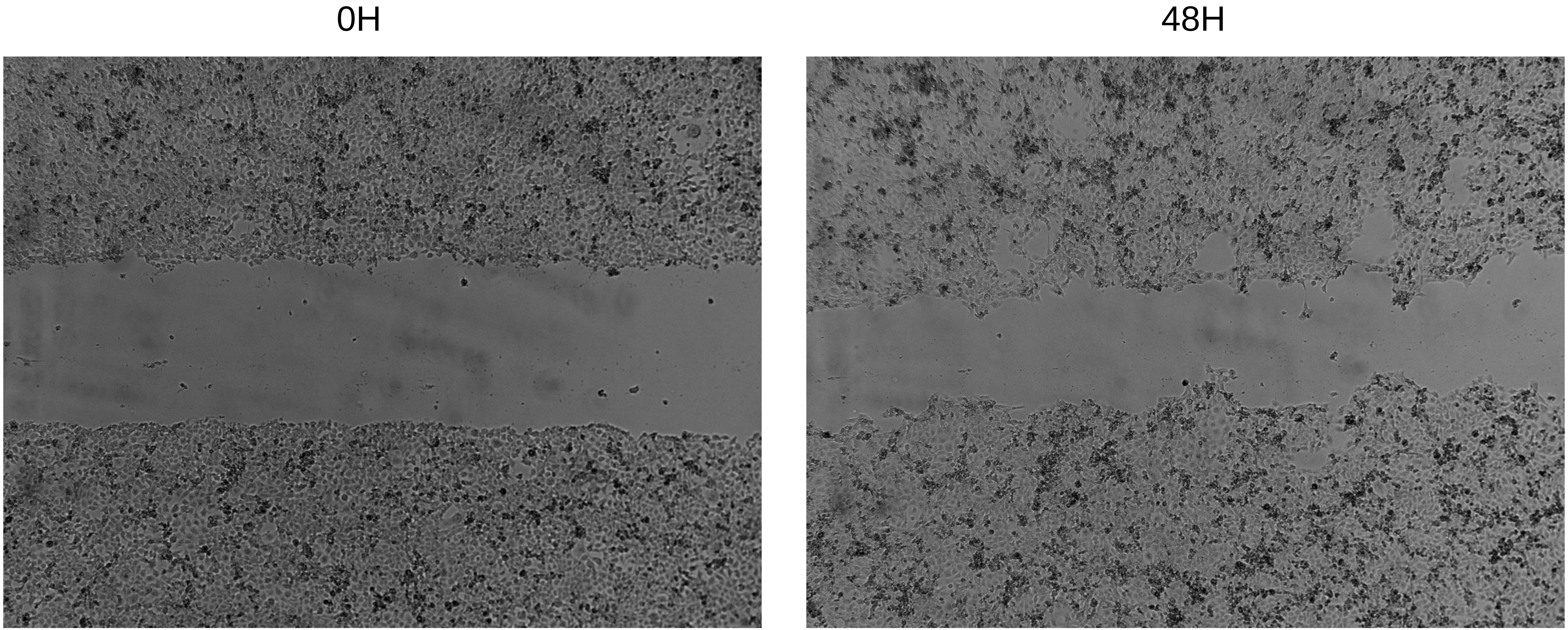

Figure s4E HSC-6 siBATF2-1

0H 48H

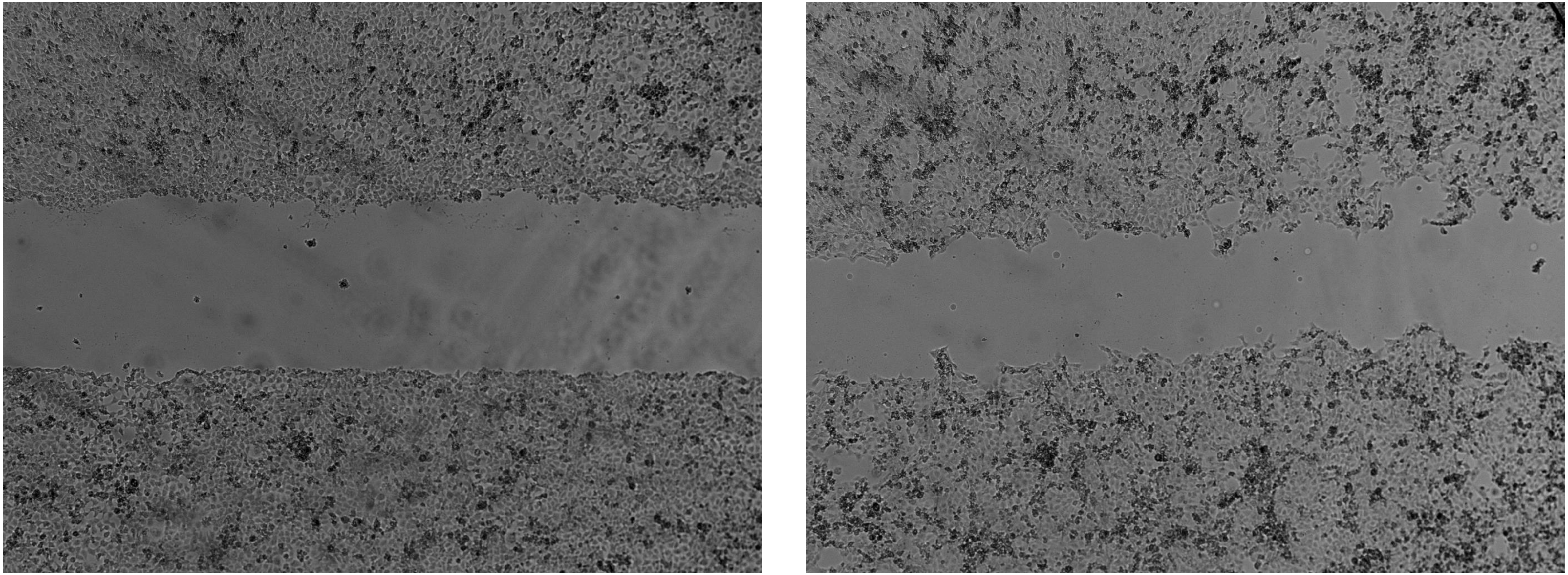

Figure s4E HSC-6 siBATF2-2

0H 48H

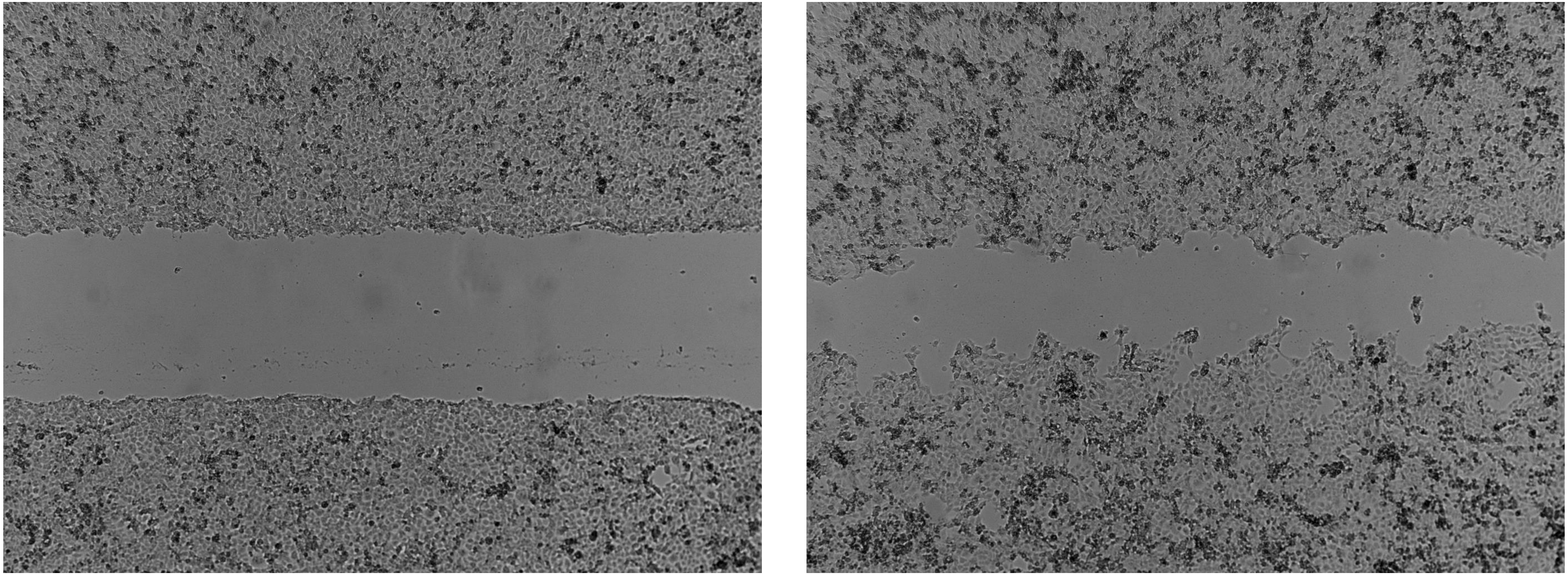

Figure s5A

Figure s5A CAL-27 siNC

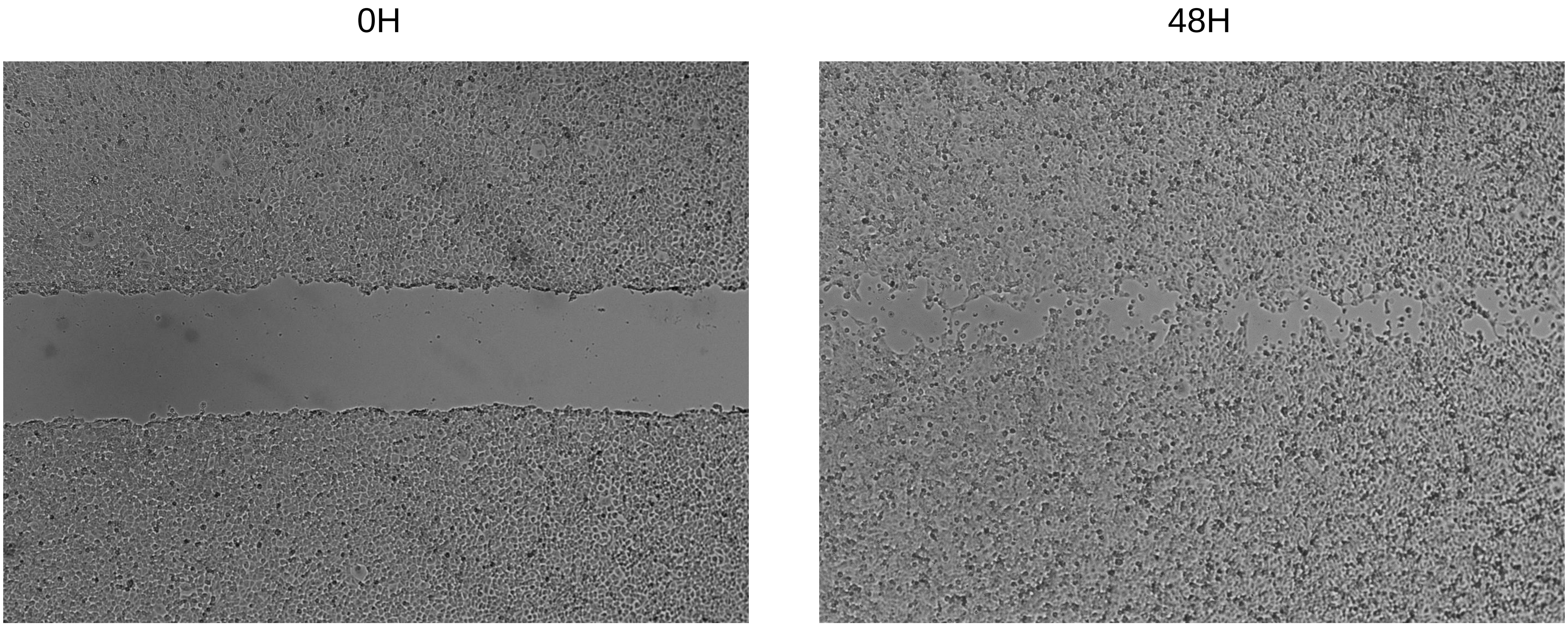

Figure s5A CAL-27 siGSDMD-1

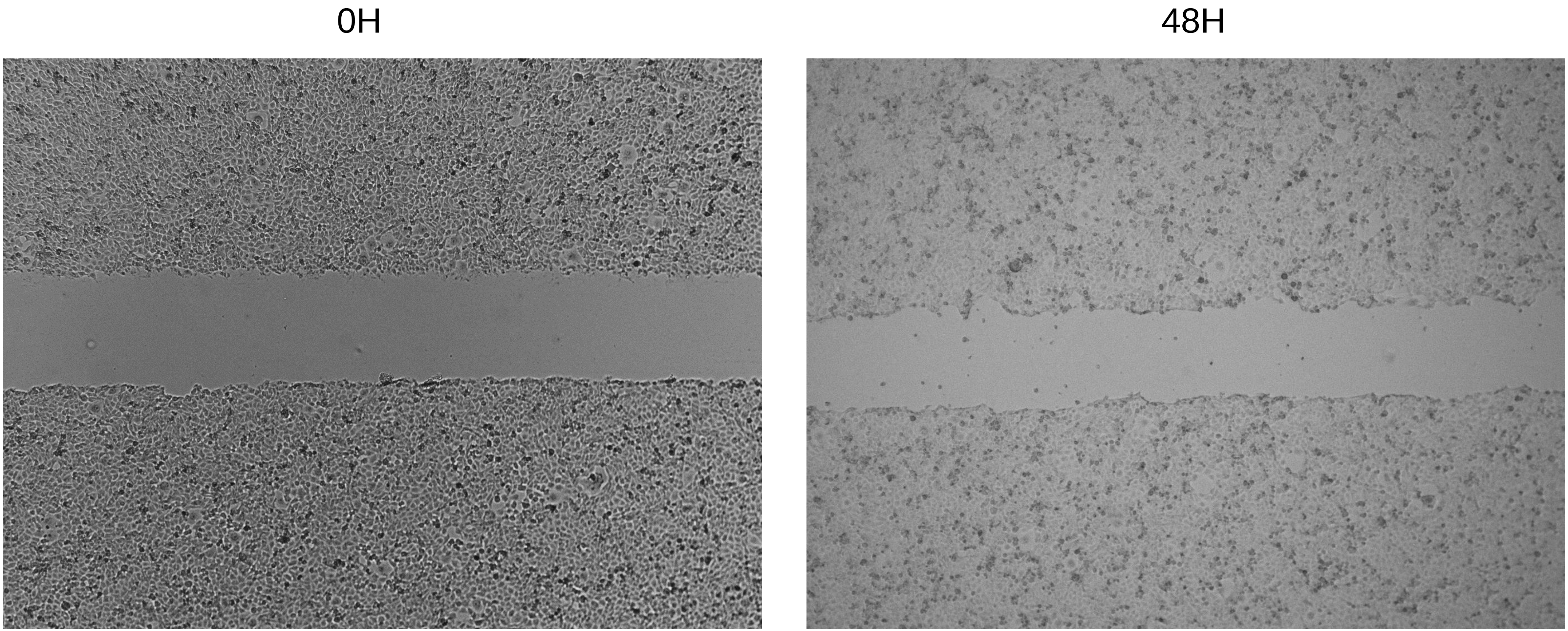

Figure s5A CAL-27 siGSDMD-1

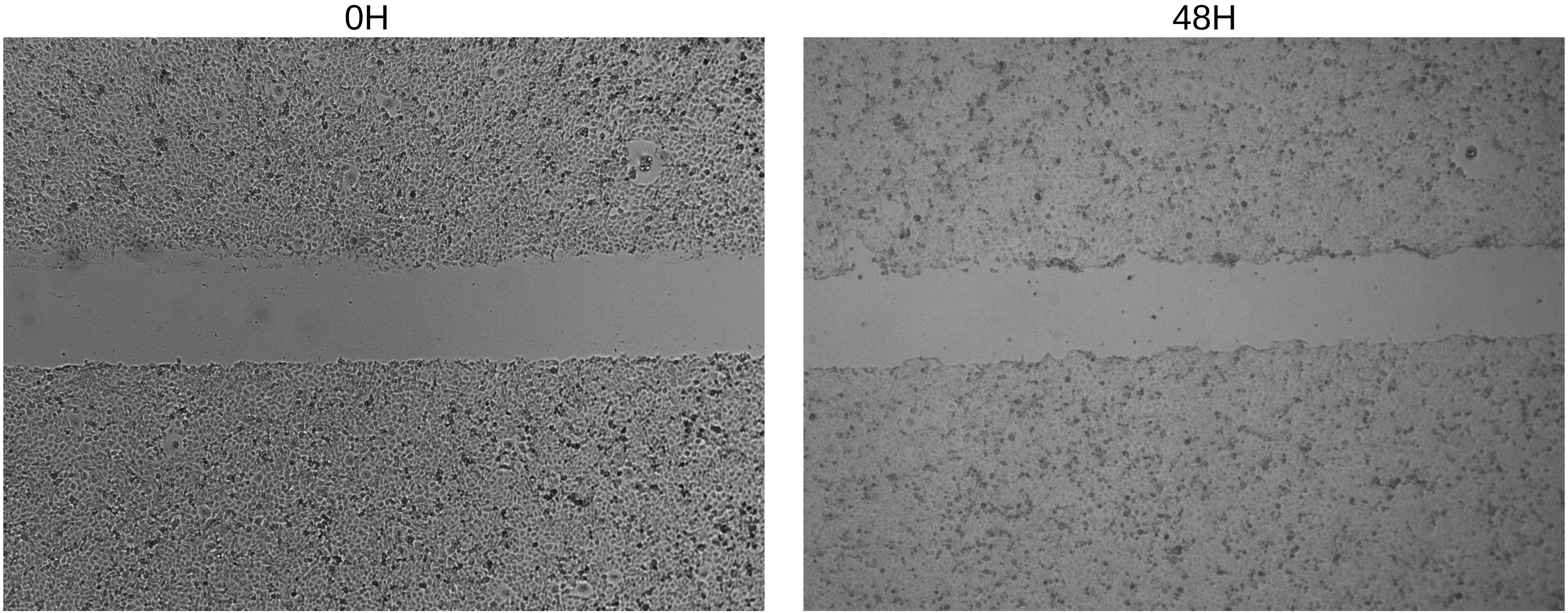

Figure s5A HSC-6 siNC

0H 48H

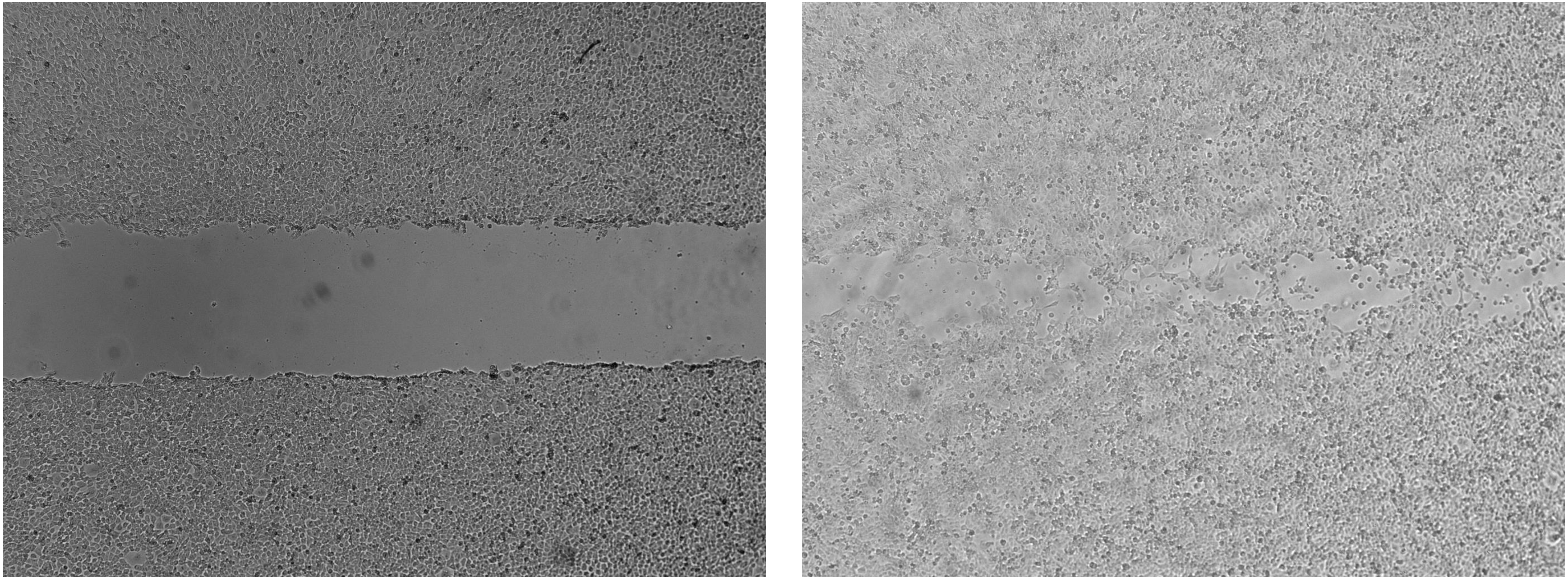

Figure s5A HSC-6 siGSDMD-1

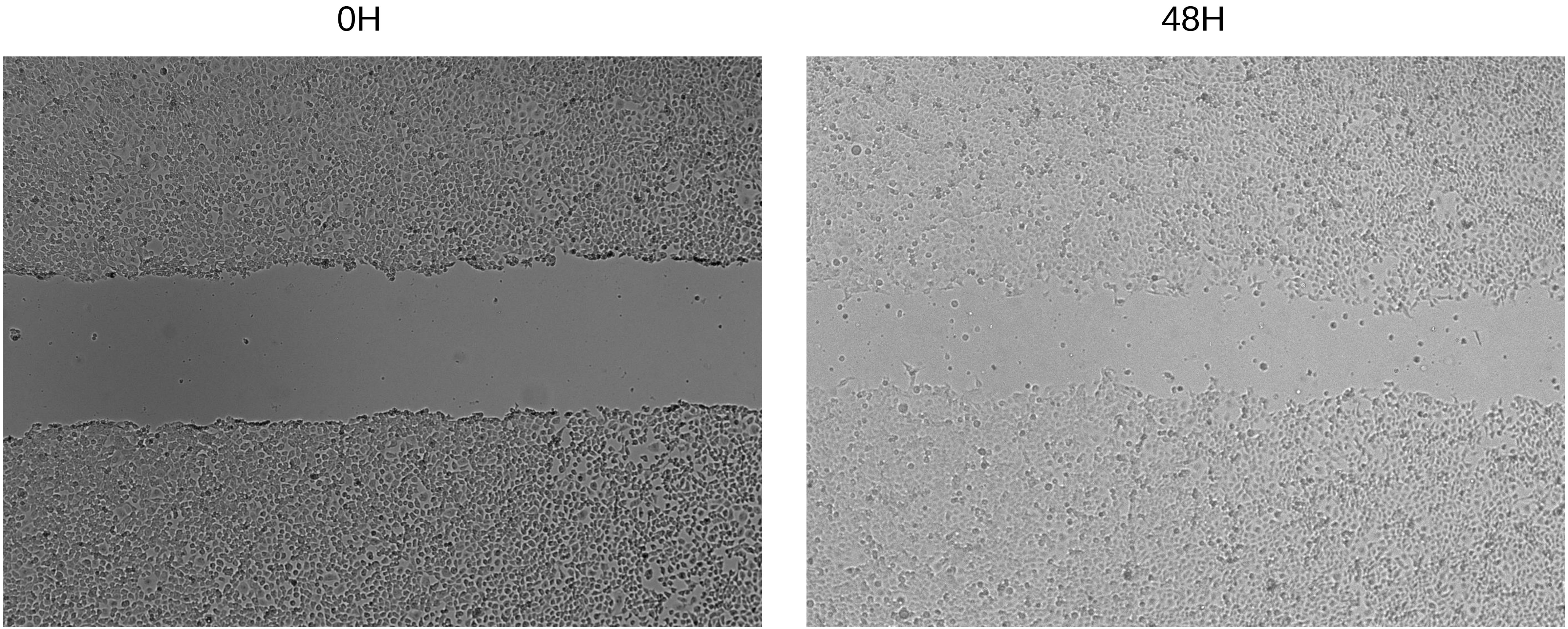

Figure s5A HSC-6 siGSDMD-2

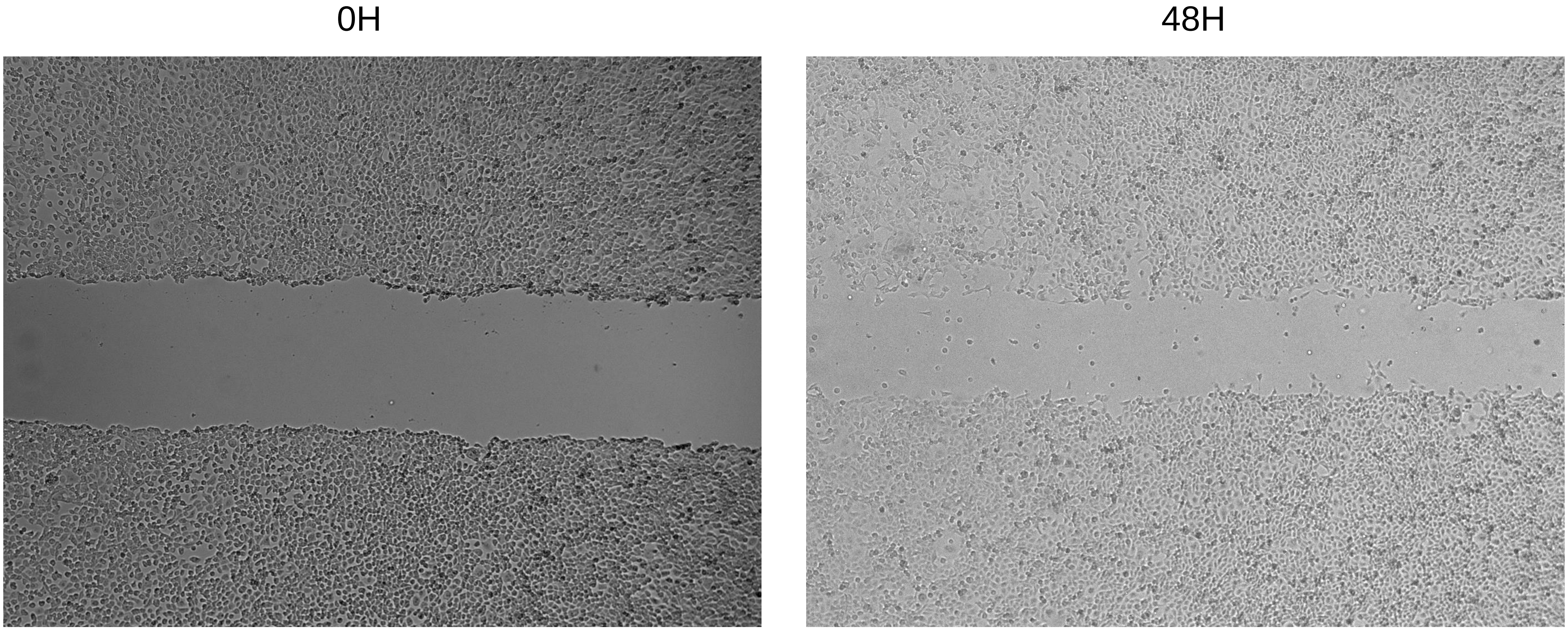

Figure s5B

Figure s5B CAL-27-vector

0H 48H

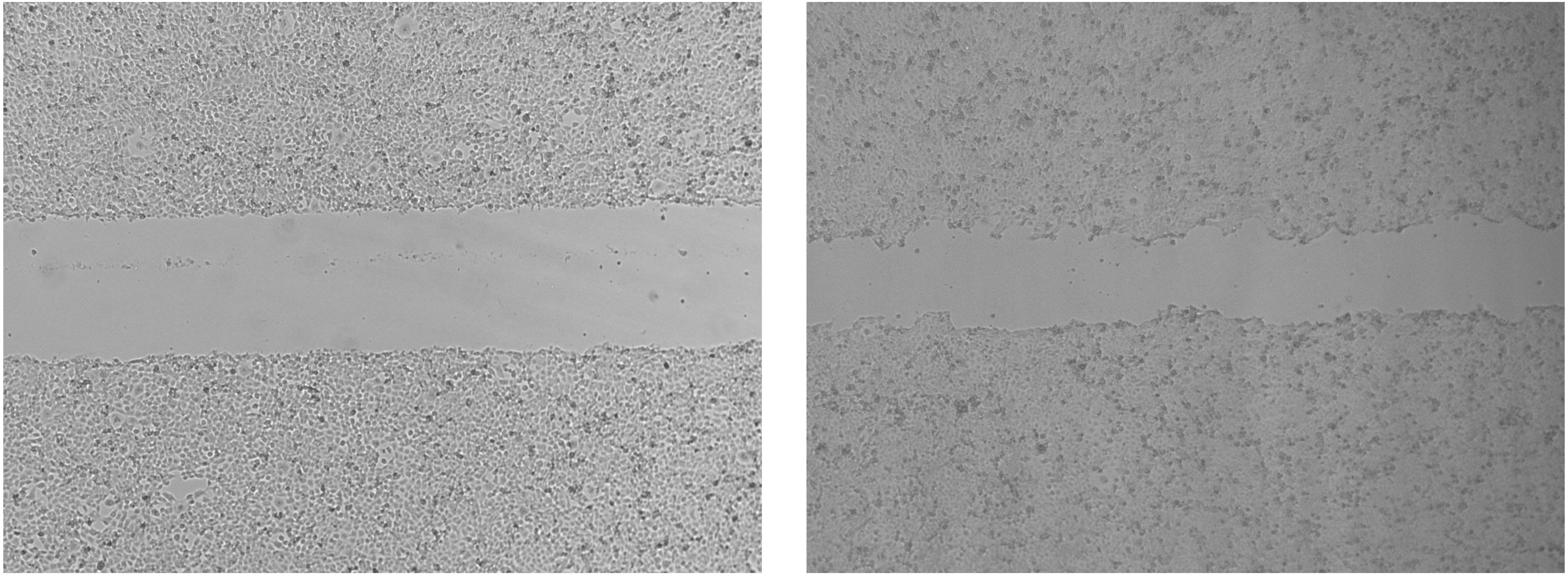

Figure s5B CAL-27-GSDMD-sh

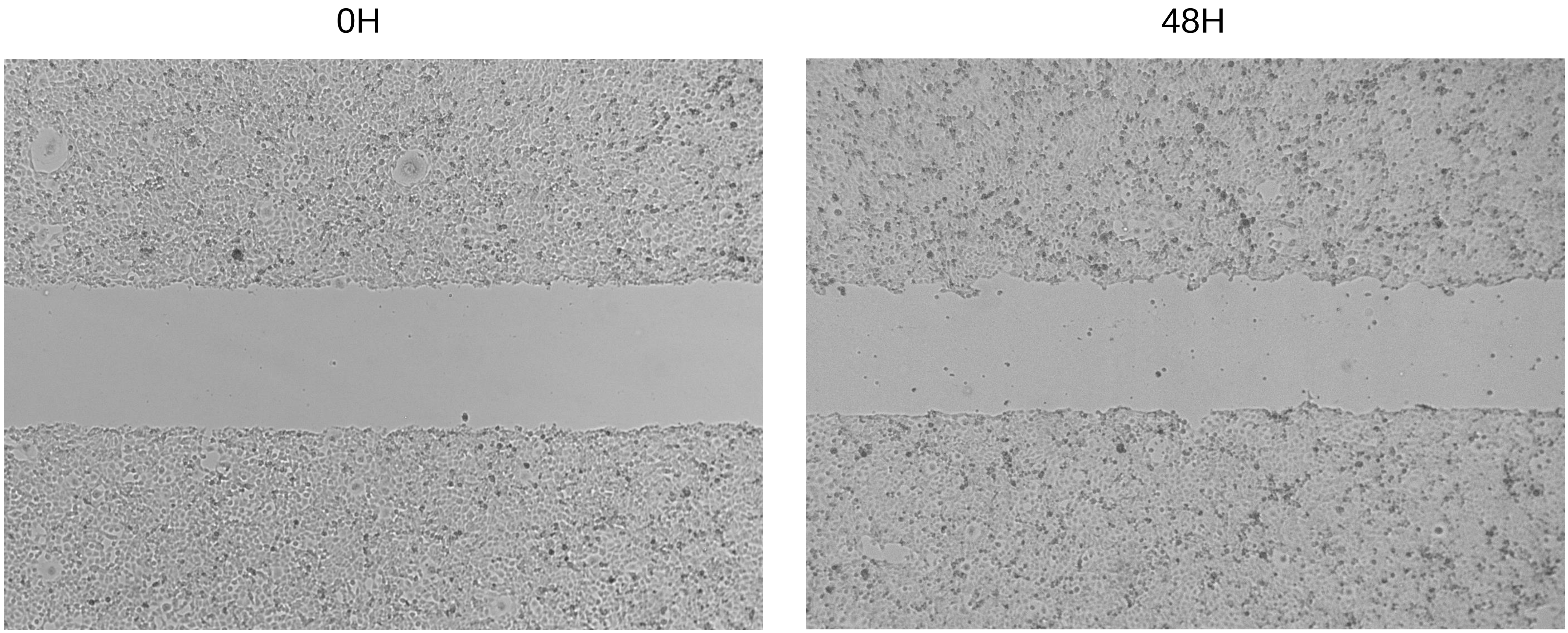

Figure s5B CAL-27-vector+Cis 1.25 μM

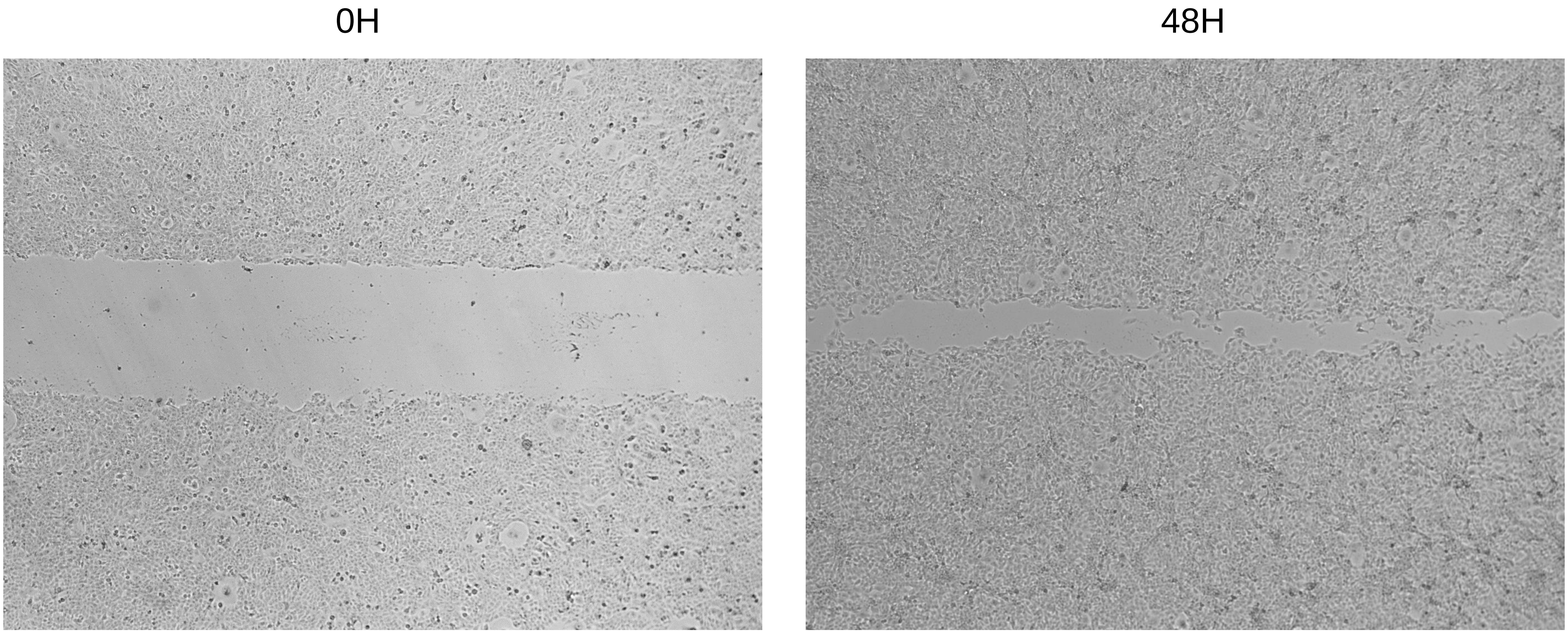

Figure s5B CAL-27-GSDMD-sh+Cis 1.25 μM

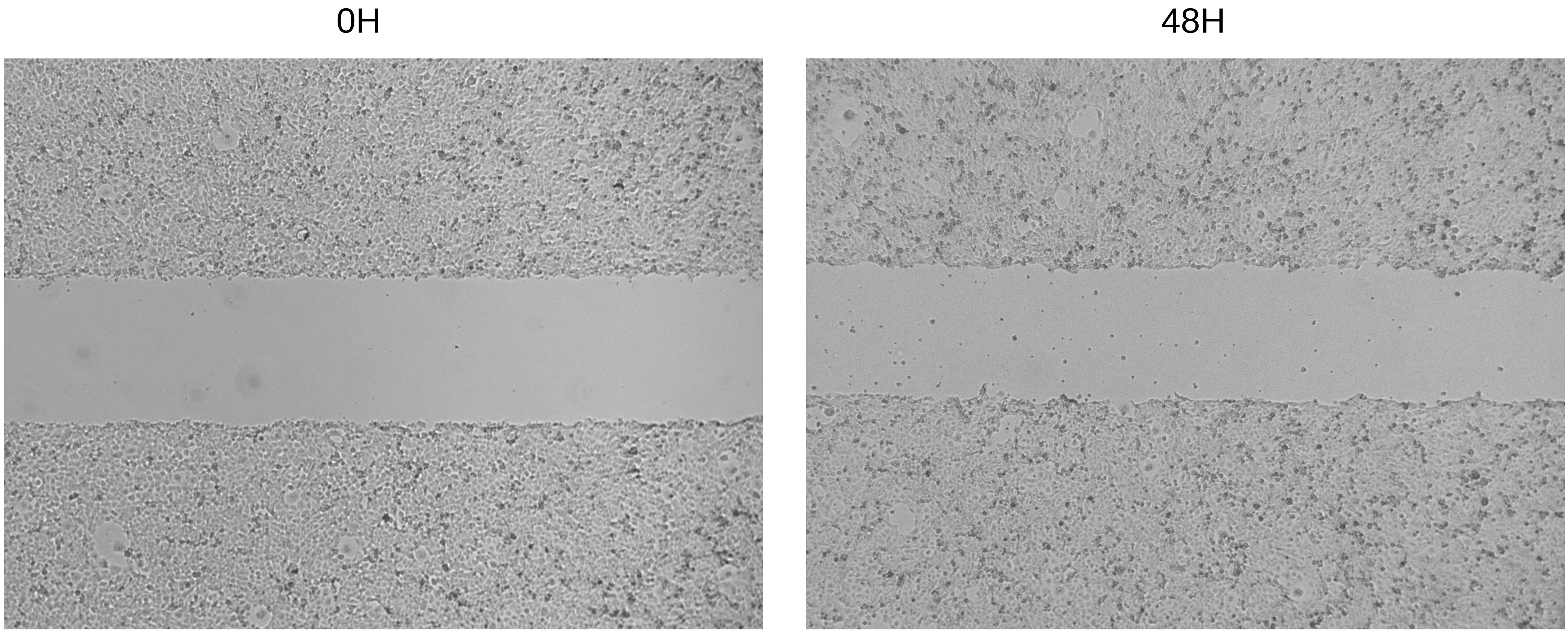

Figure s5B CAL-27-GSDMD-sh+Cis 2.5 μM

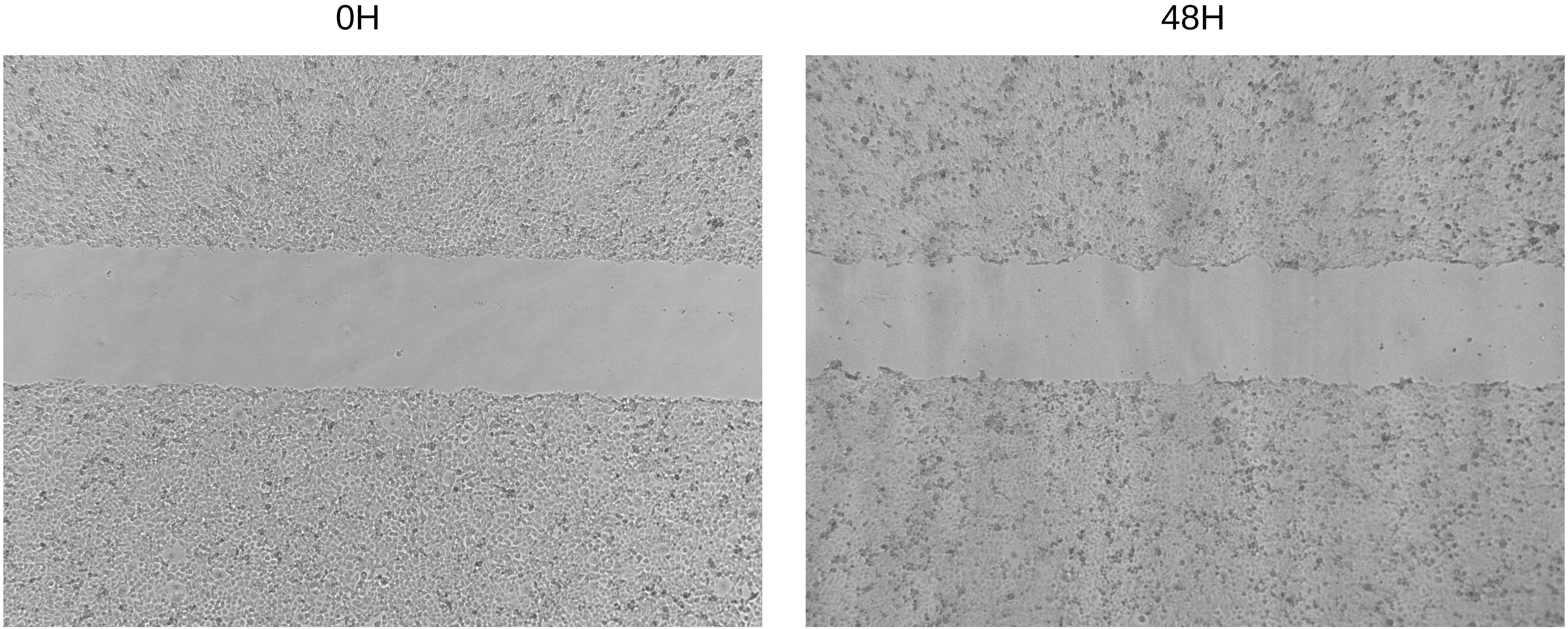

Figure s5B HSC-6-vector

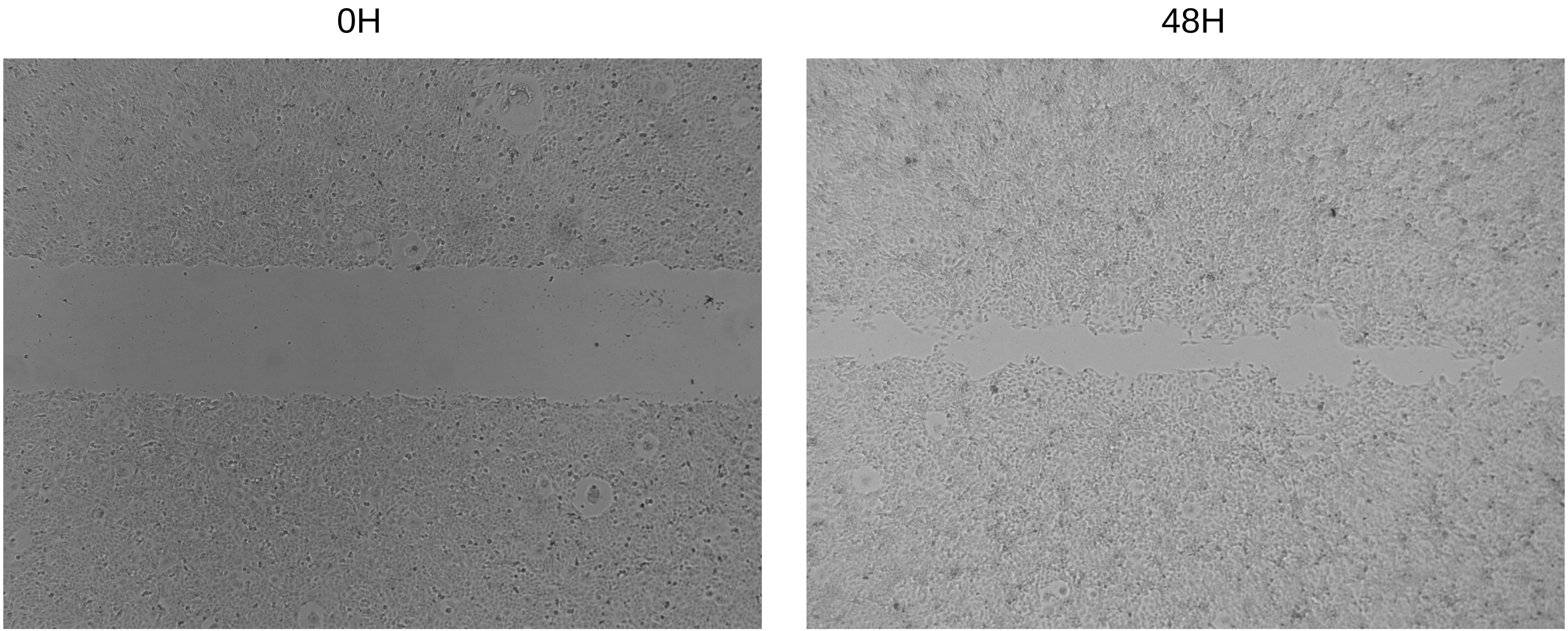

Figure s5B HSC-6-GSDMD-sh

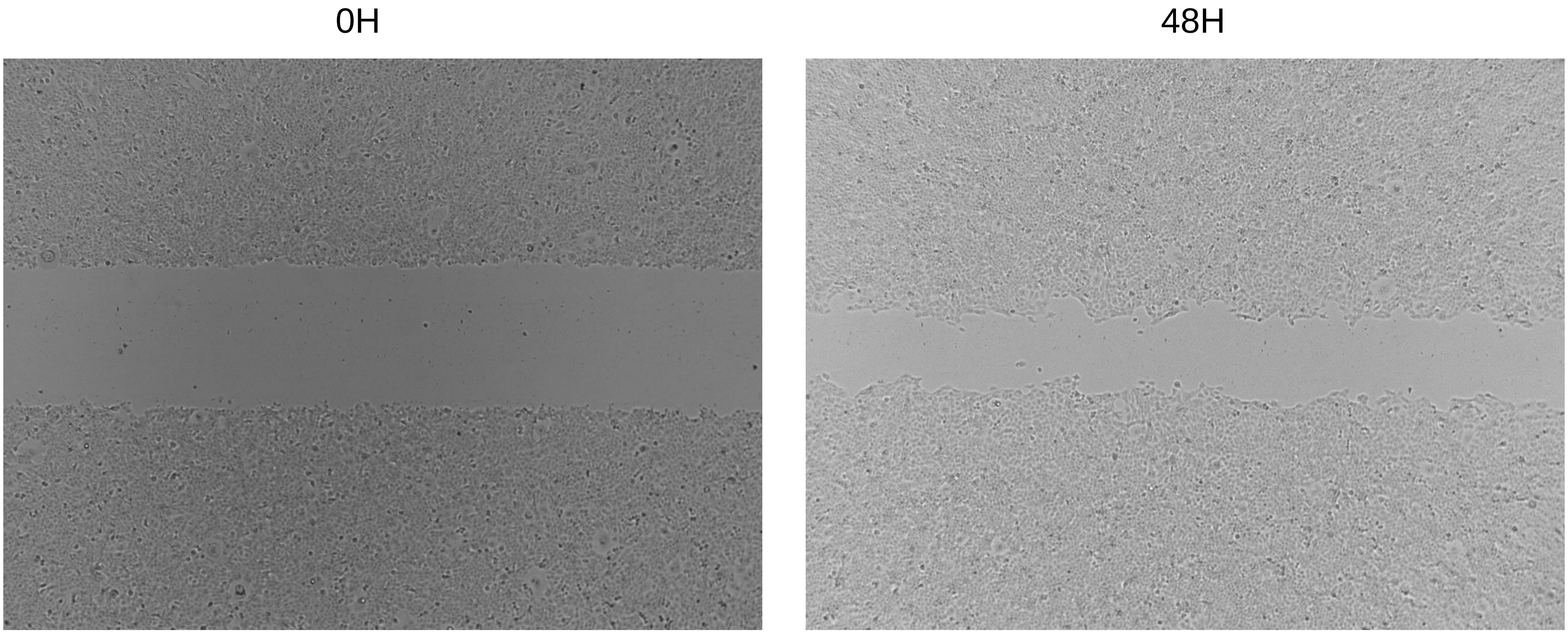

Figure s5B HSC-6-vector+Cis 1.25 μM

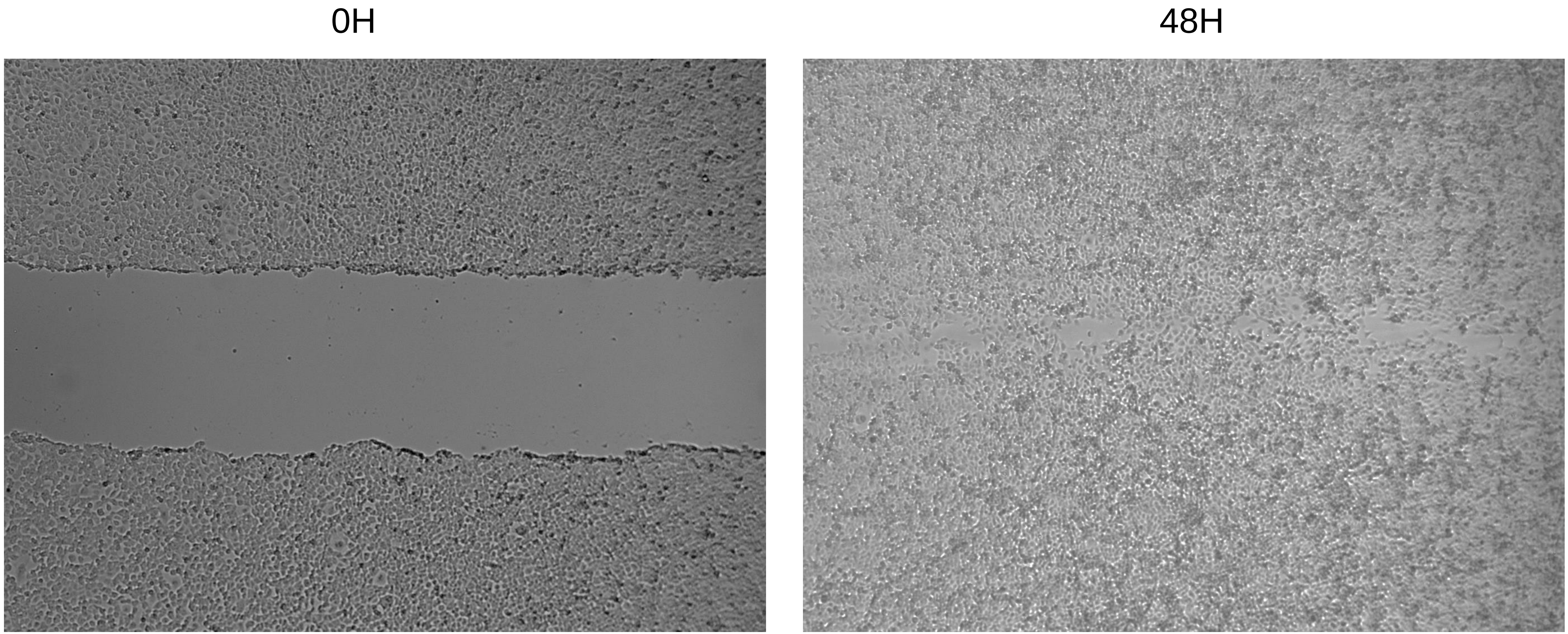

Figure s5B HSC-6-GSDMD-sh+Cis 1.25 μM

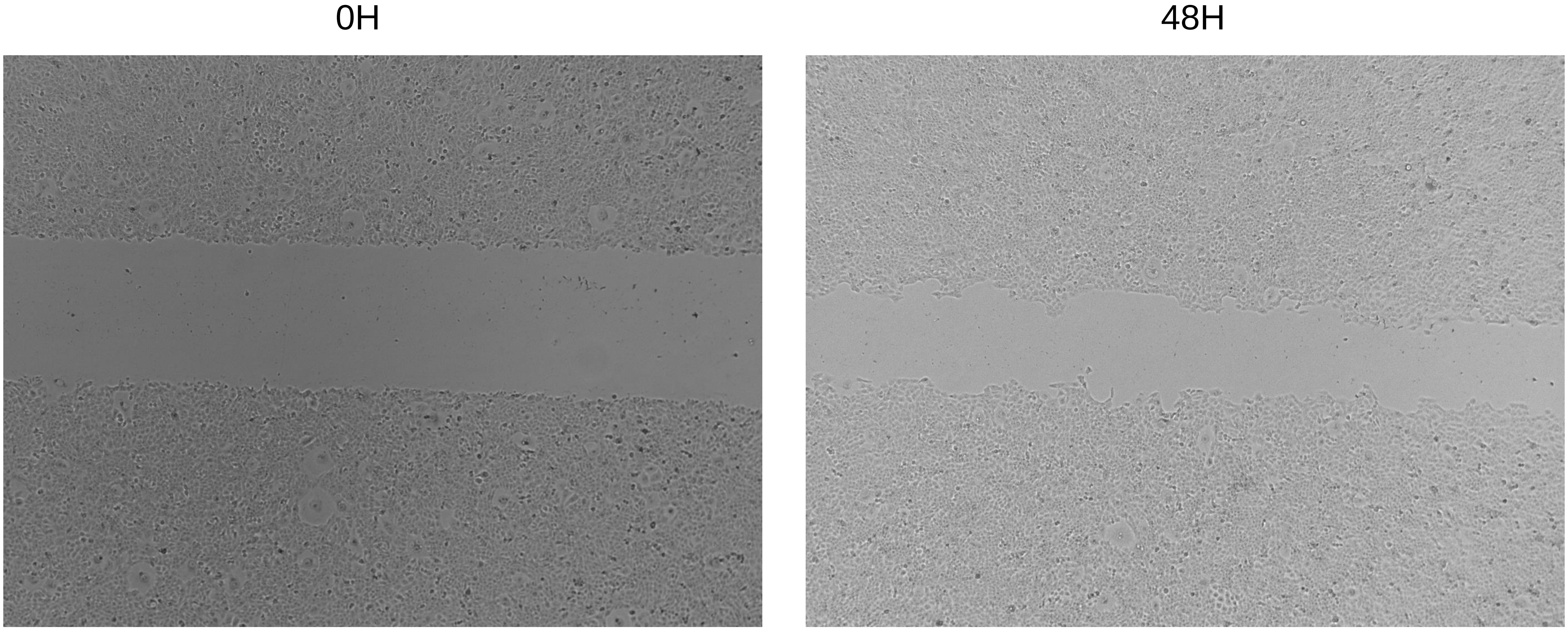

Figure s5B HSC-6-GSDMD-sh+Cis 2.5 μM

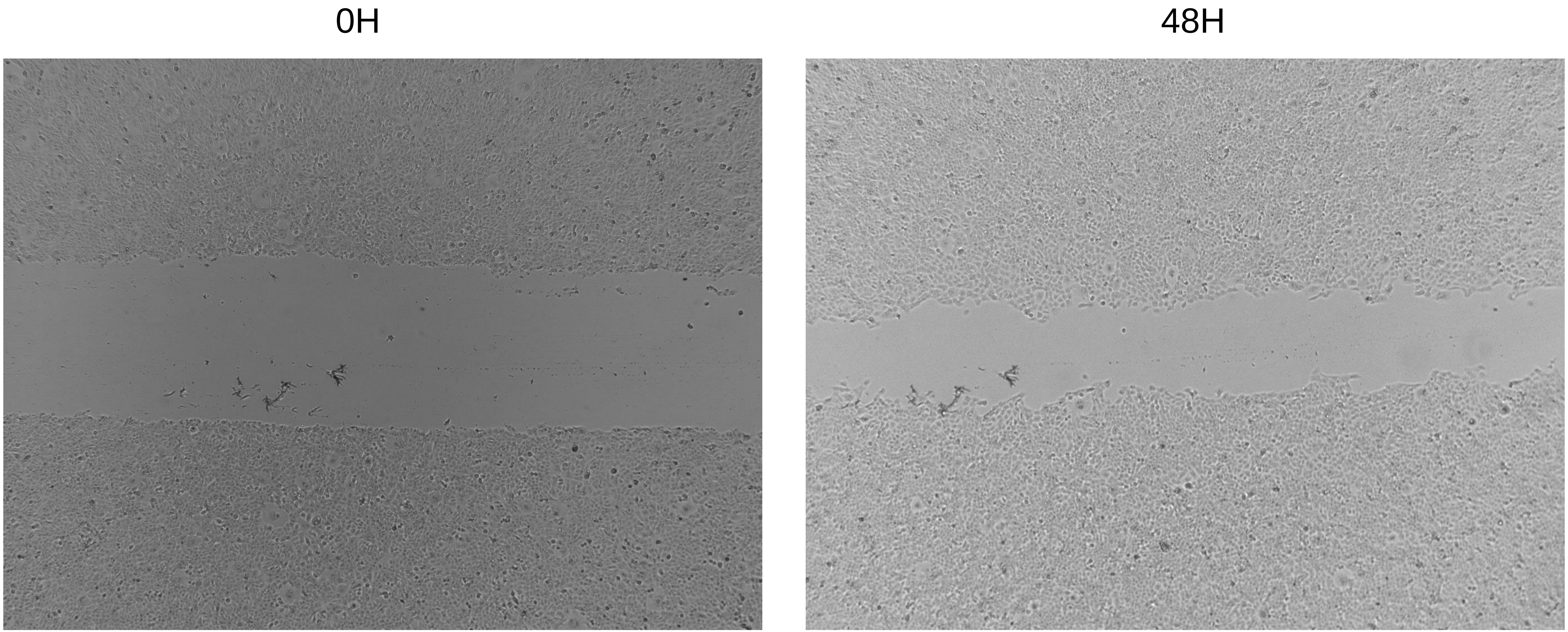

Figure s8C

Figure s8C CAL-27-vector

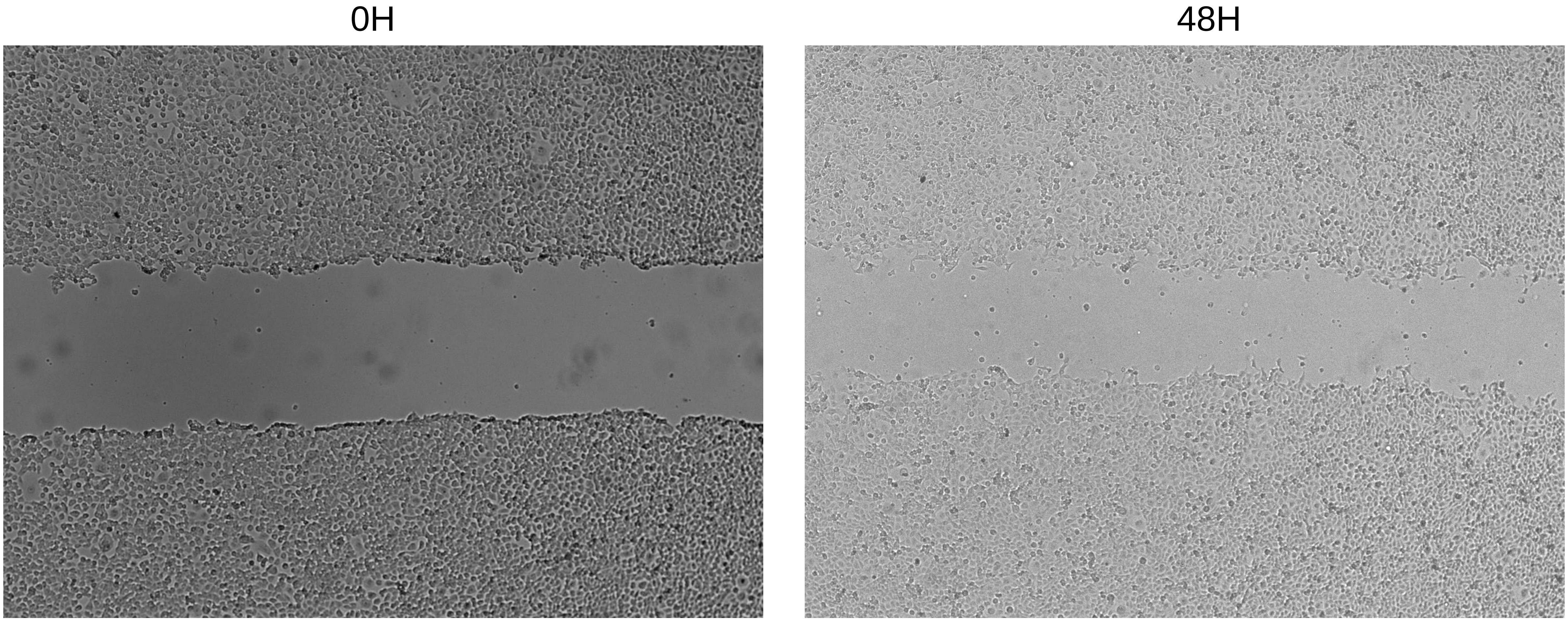

Figure s8C CAL-27-GSDMD-ov

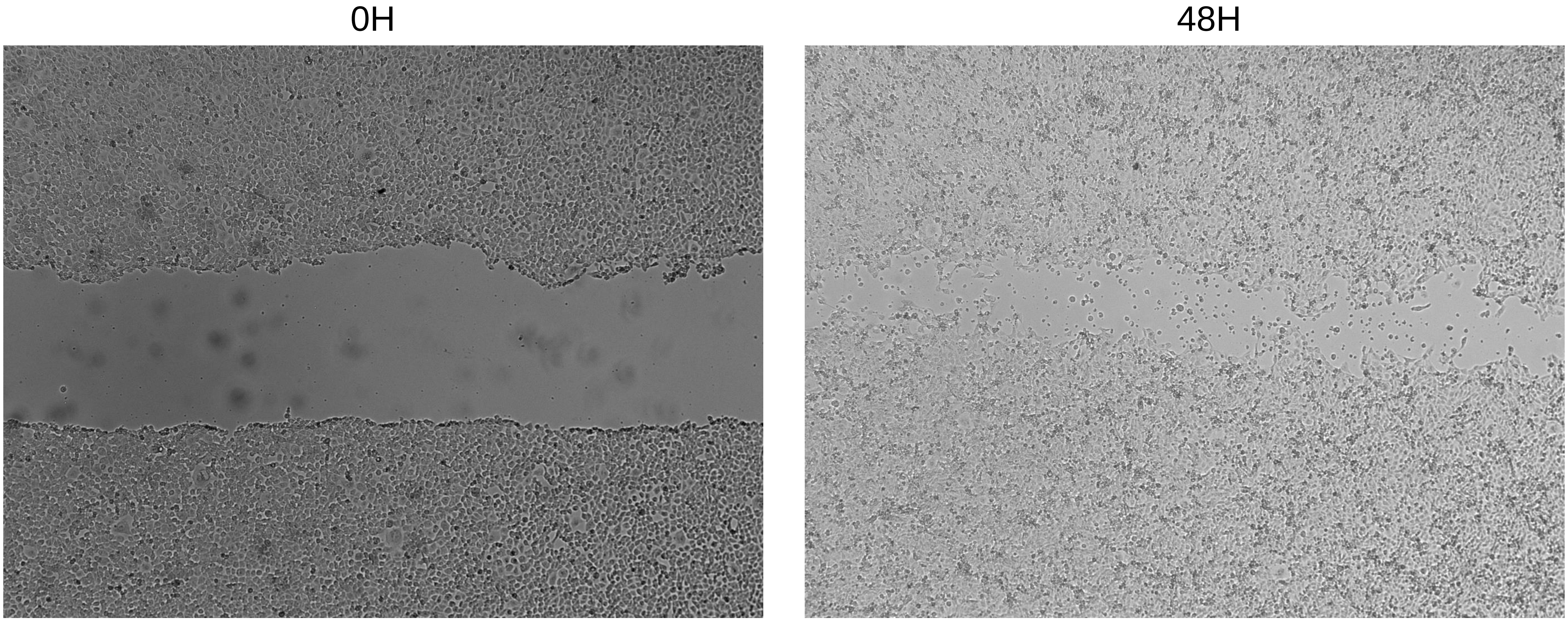

Figure s8C HSC-6-vector

0H

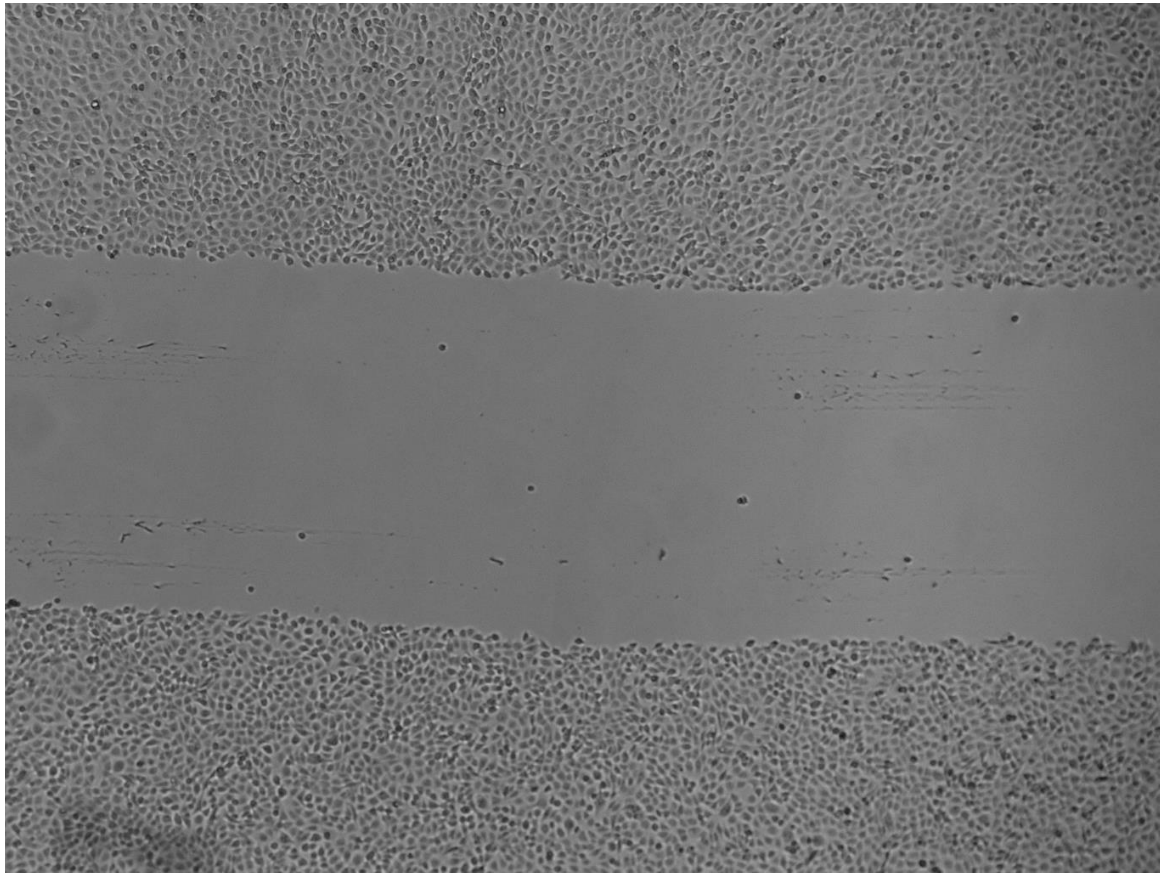

48H

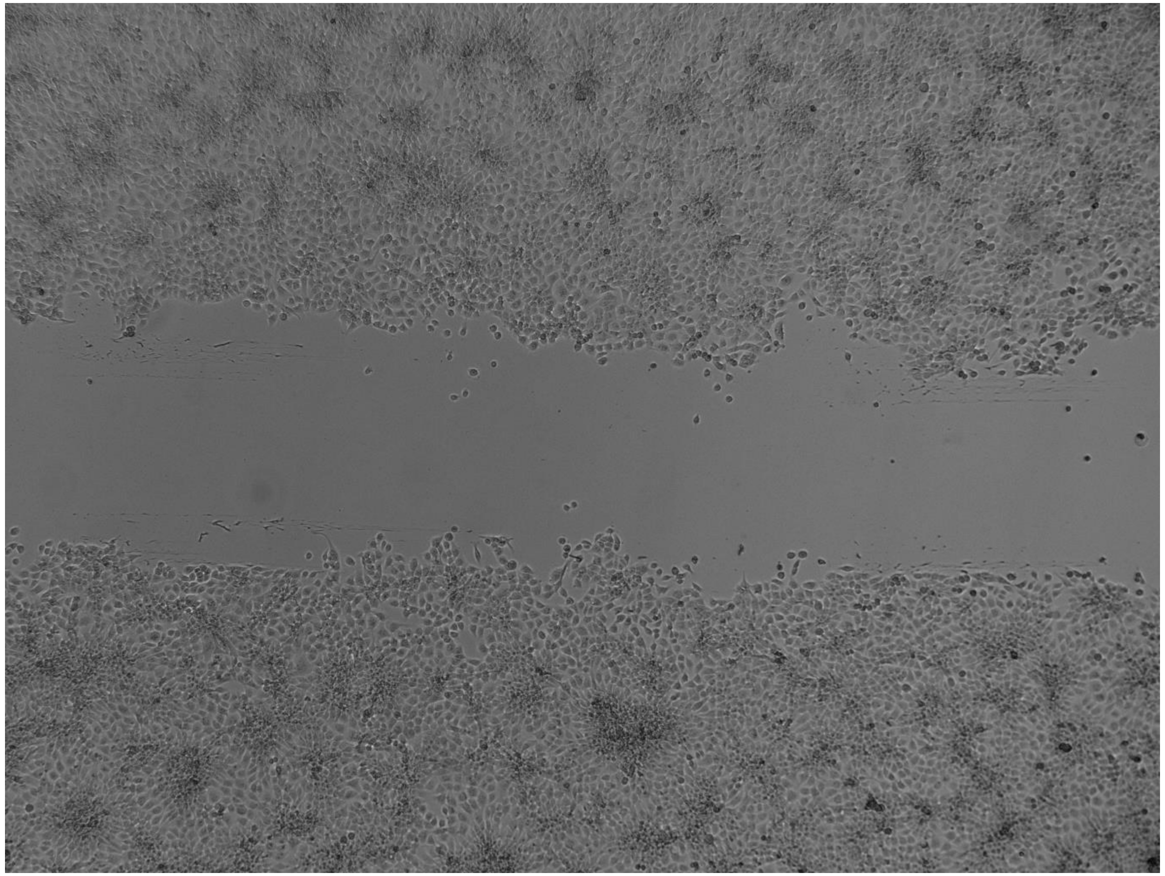

Figure s8C HSC-6-GSDMD-ov

0H

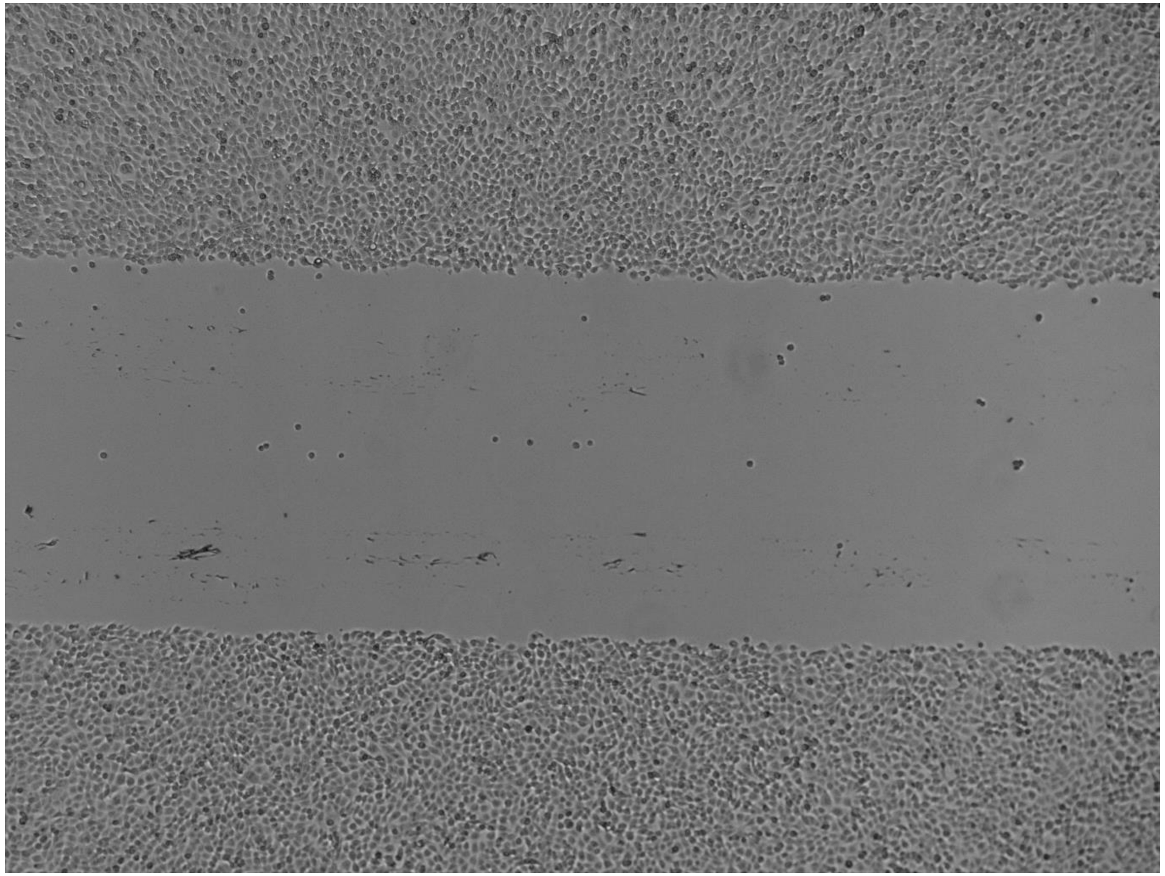

48H

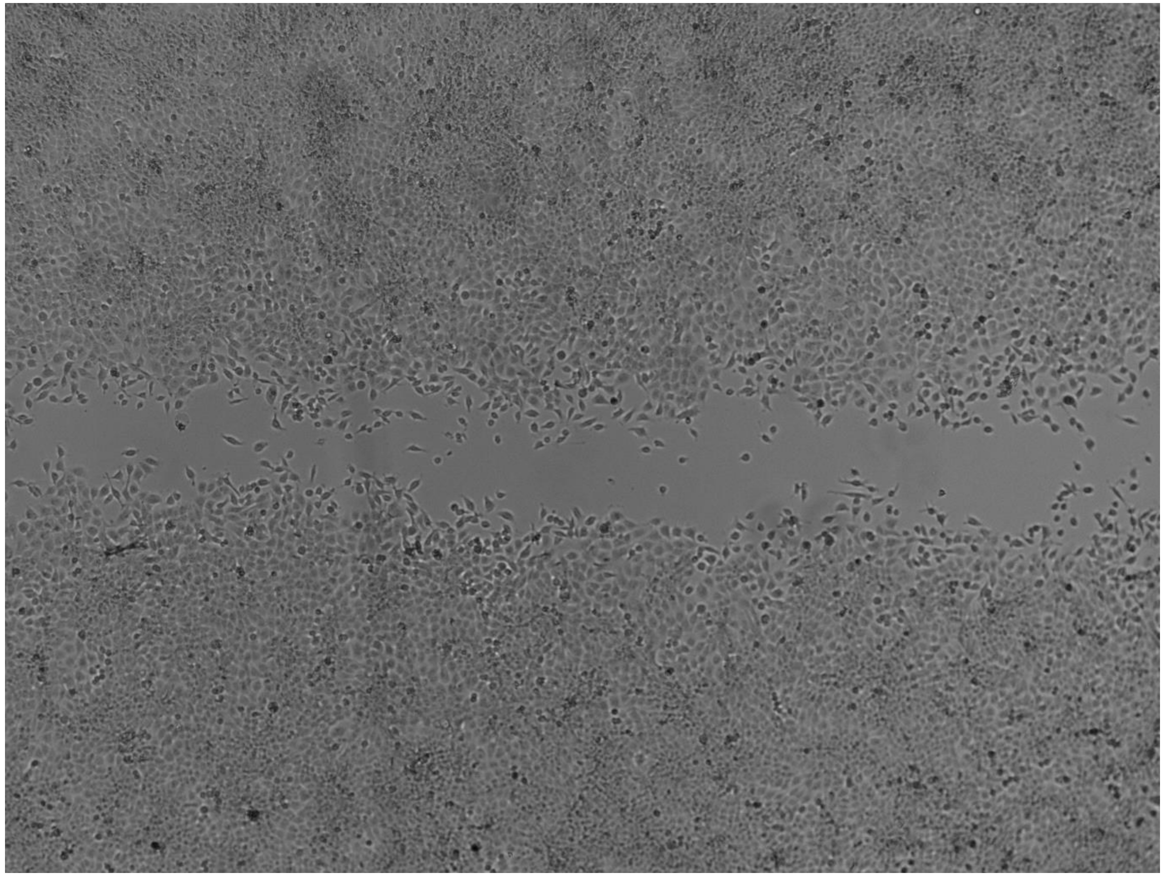

Figure s10E

Figure s10E CAL-27 siNC

0H 48H

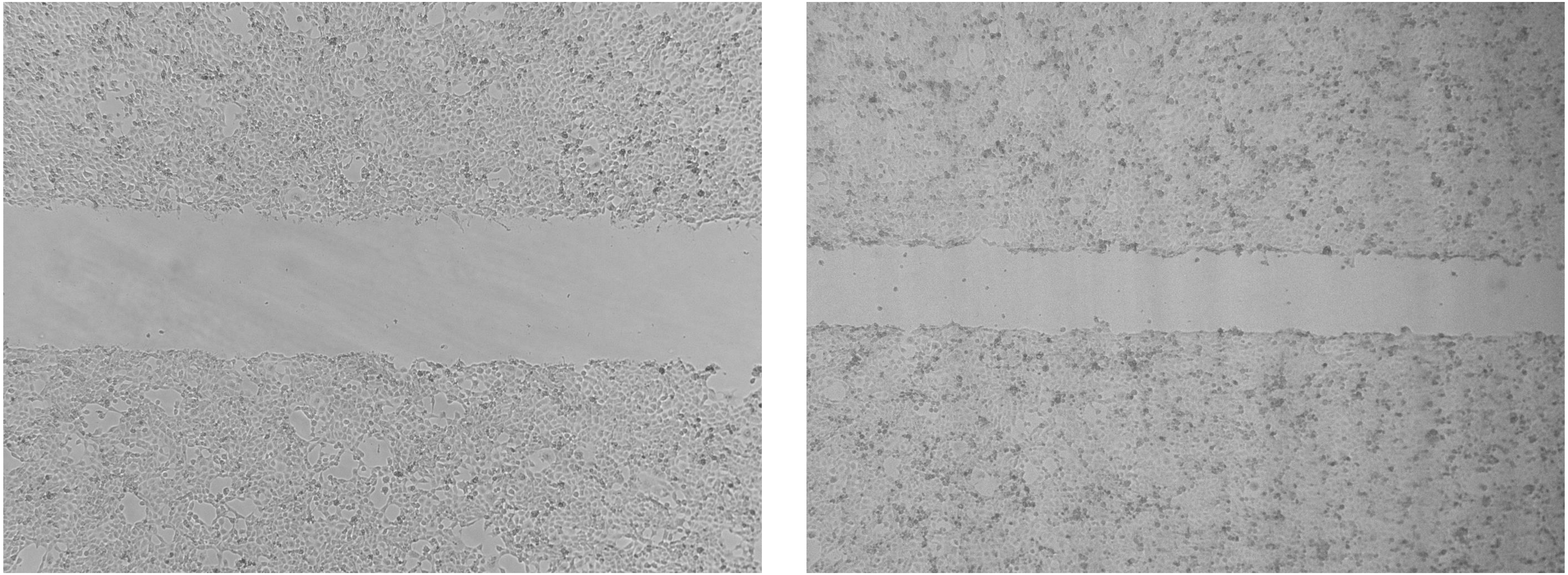

Figure s10E CAL-27 siMMP14-1

0H 48H

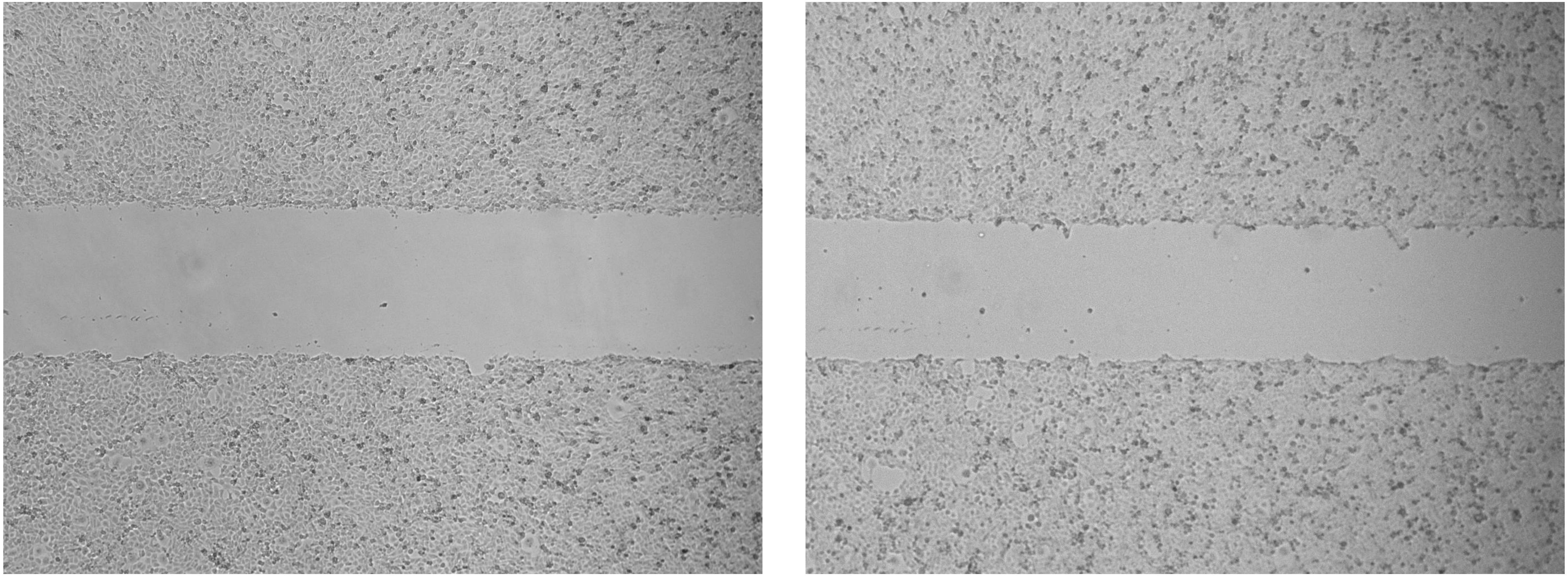

Figure s10E CAL-27 siMMP14-2

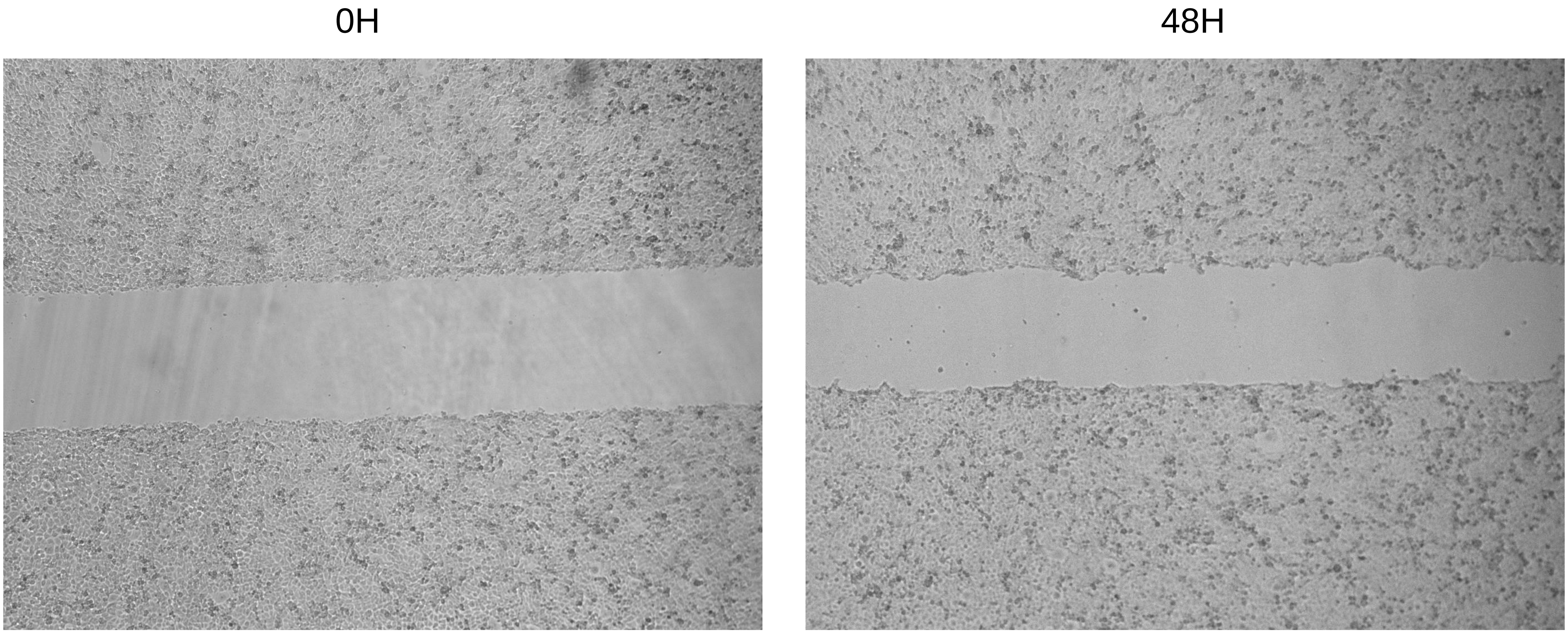

Figure s10E HSC-6 siNC

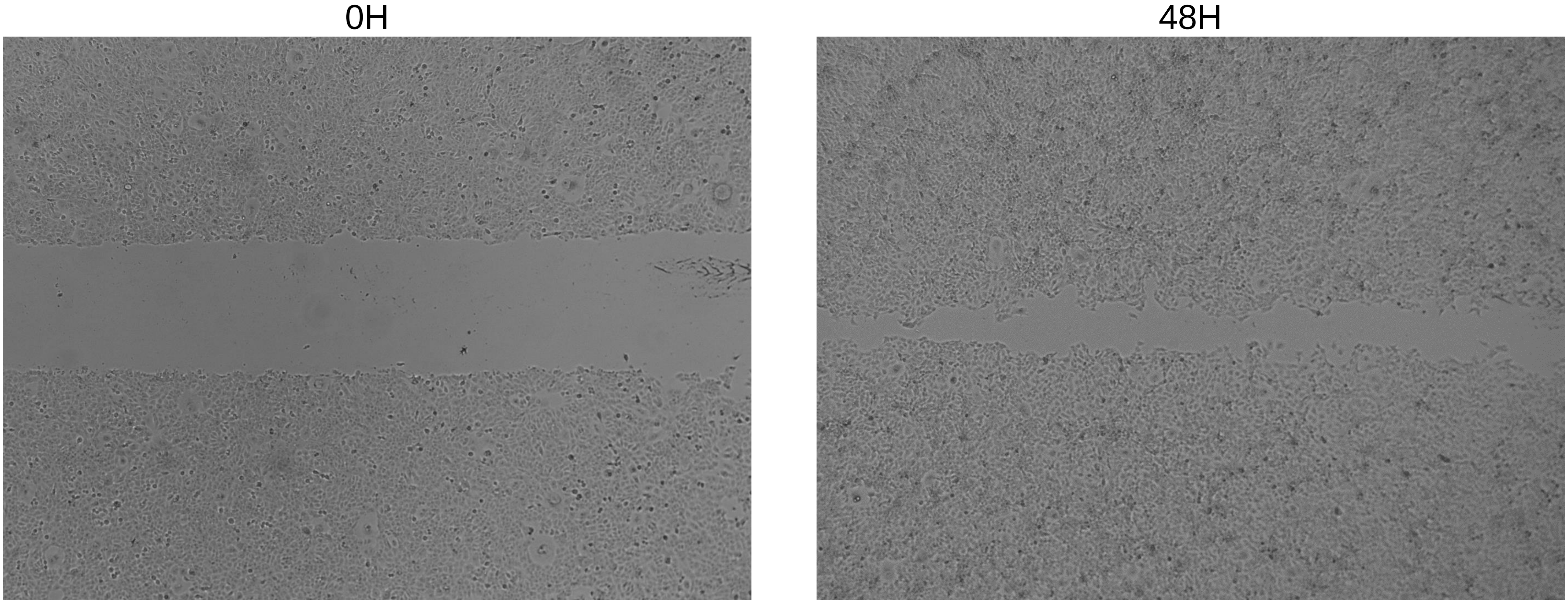

Figure s10E HSC-6 siMMP14-1

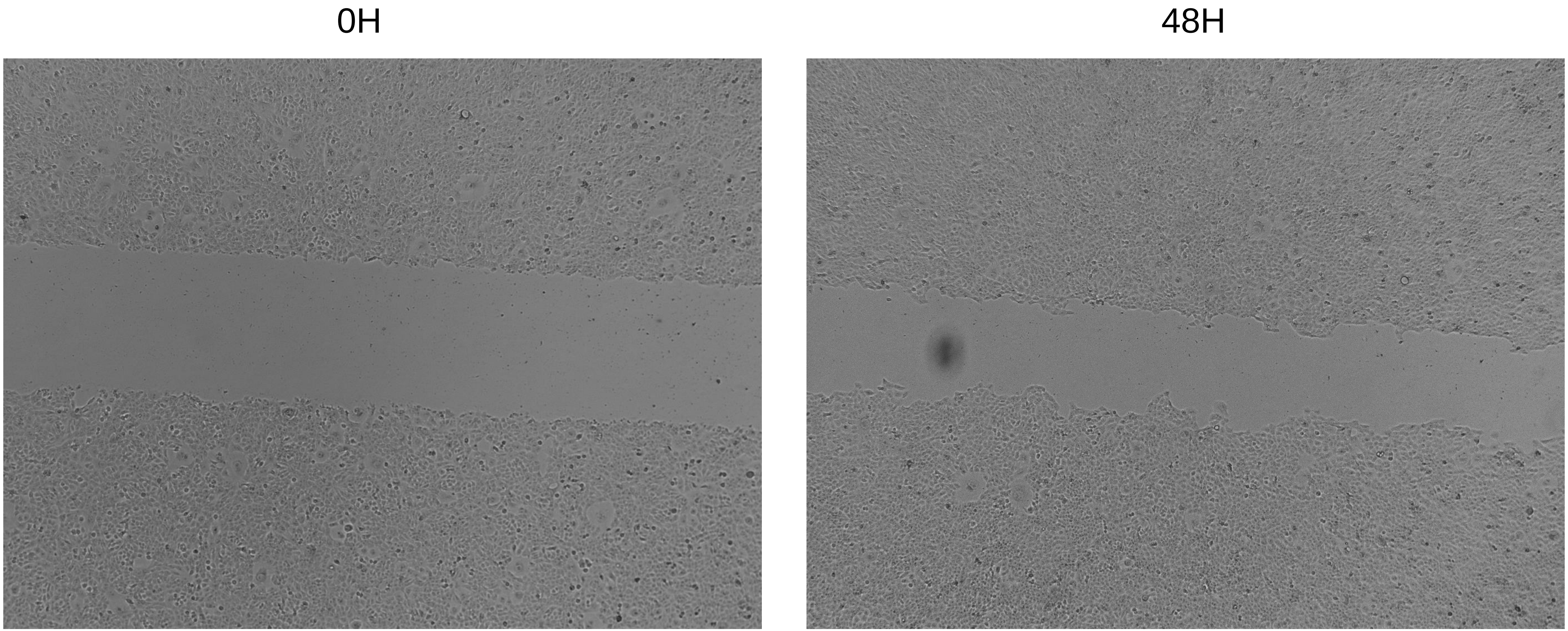

Figure s10E HSC-6 siMMP14-2

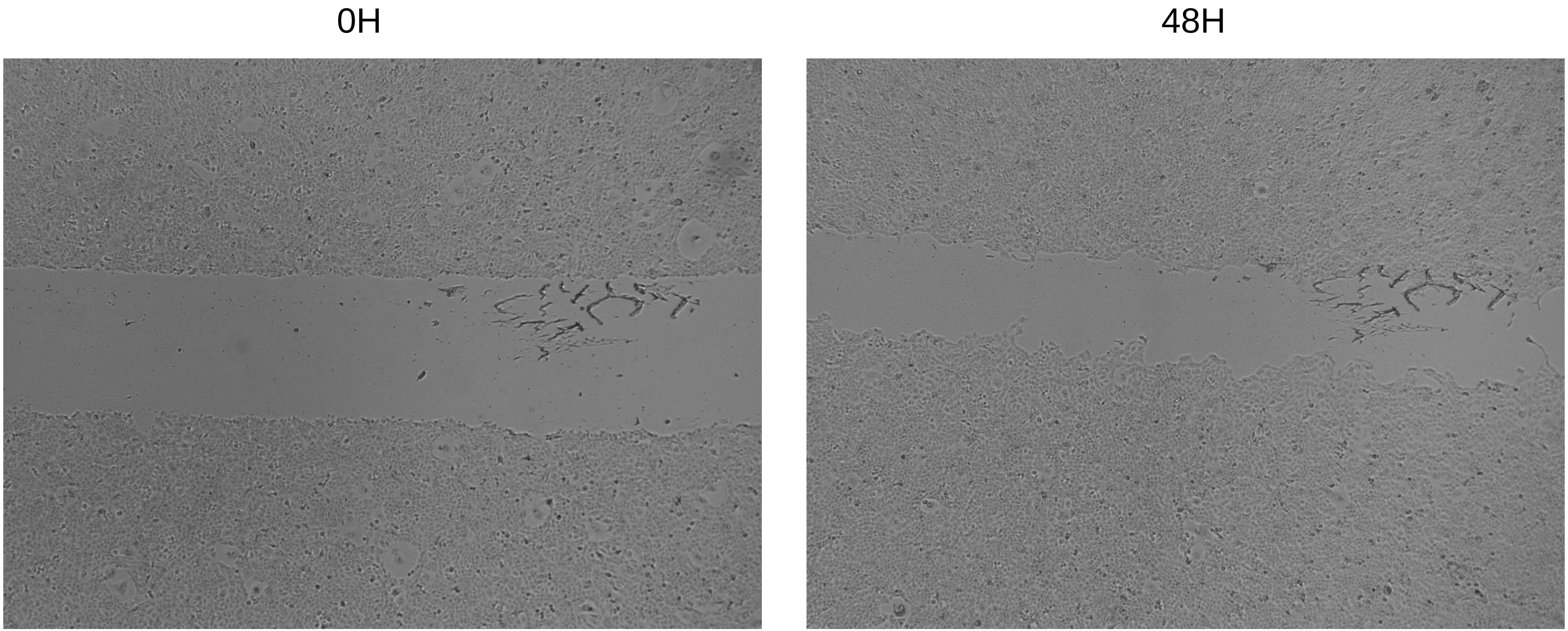

Figure s11

Figure s11 CAL-27-Ctrl

0H 48H

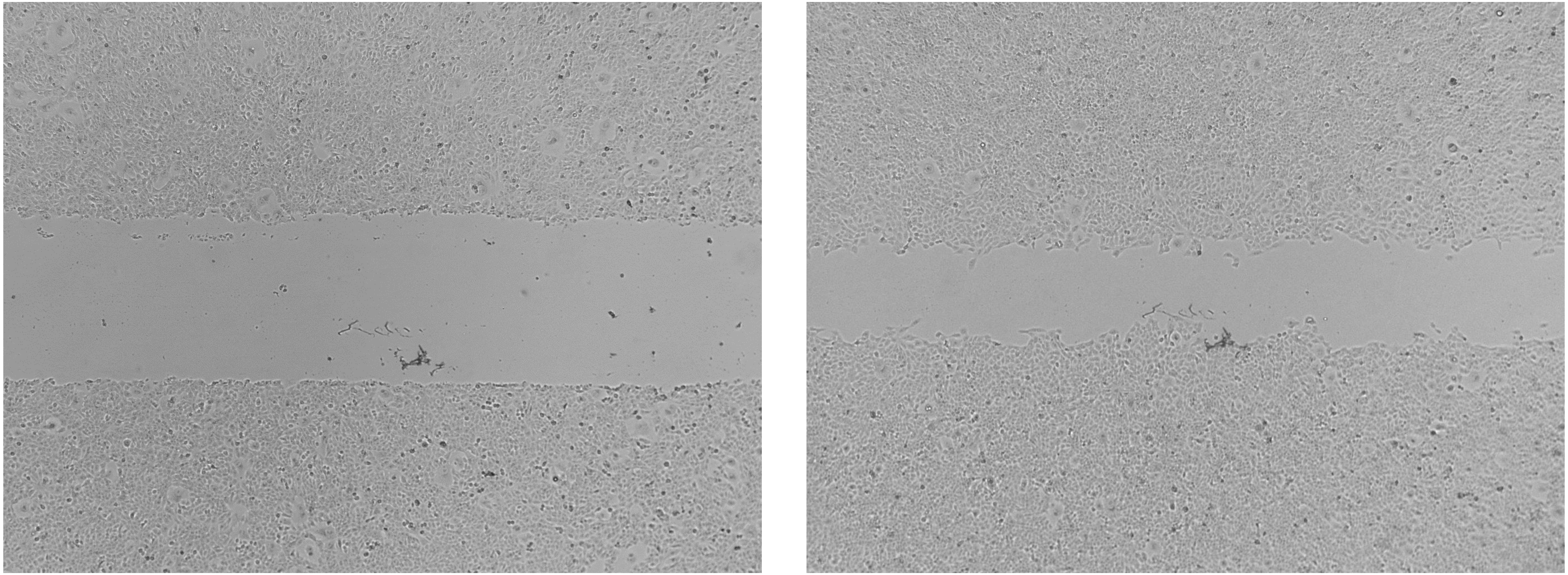

Figure s11 CAL-27-wt

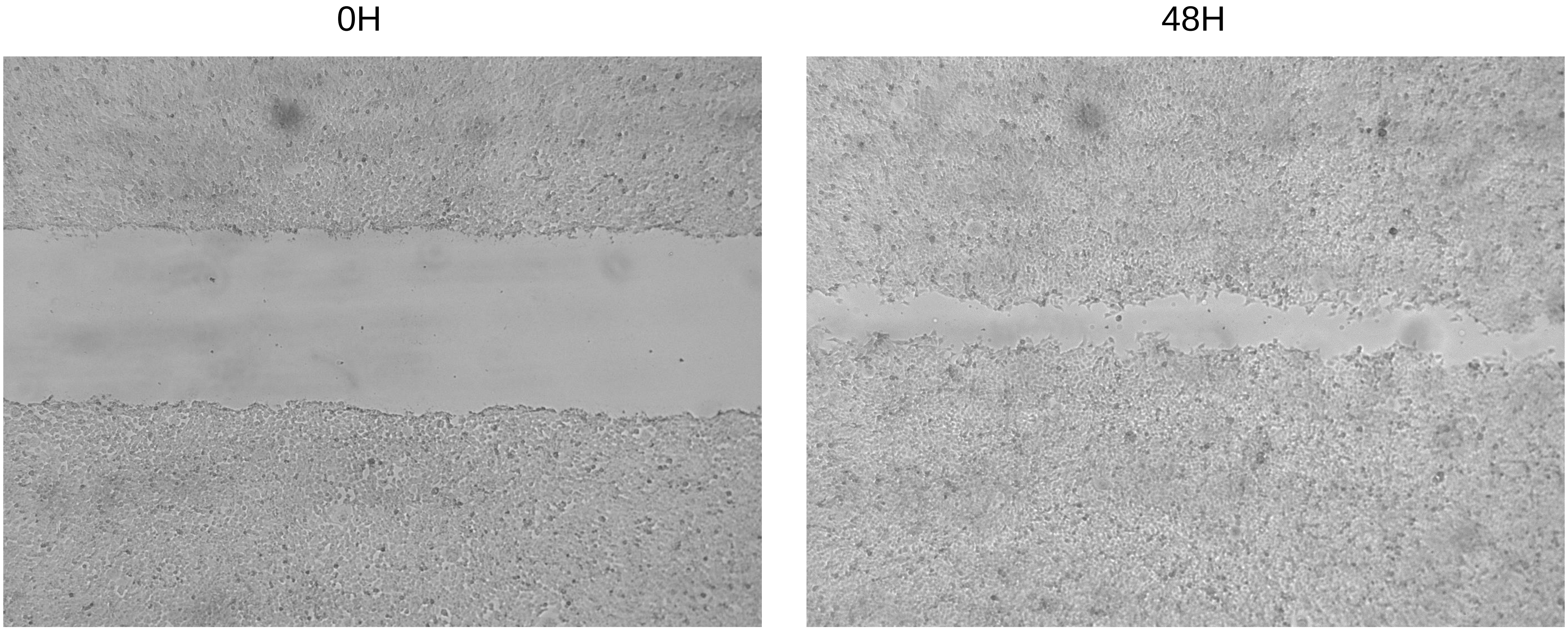

Figure s11 CAL-27-mut

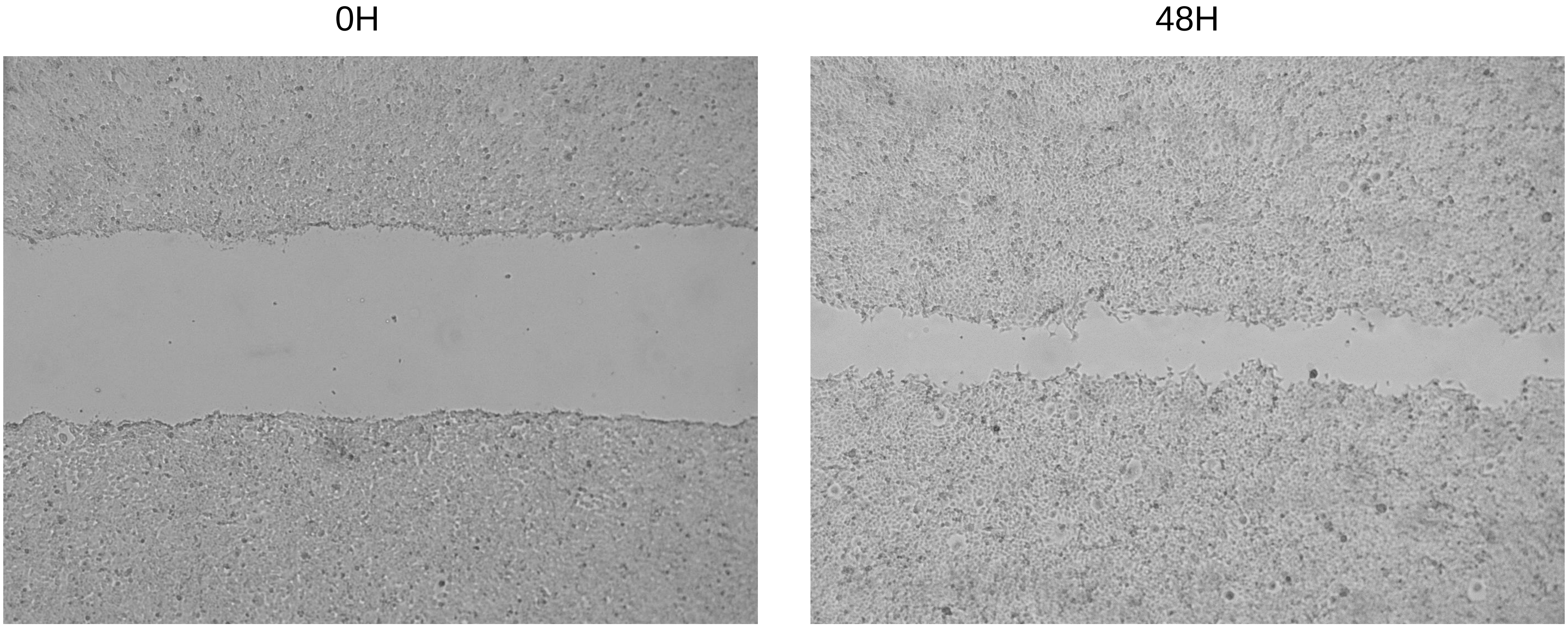

Figure s11 CAL-27-C

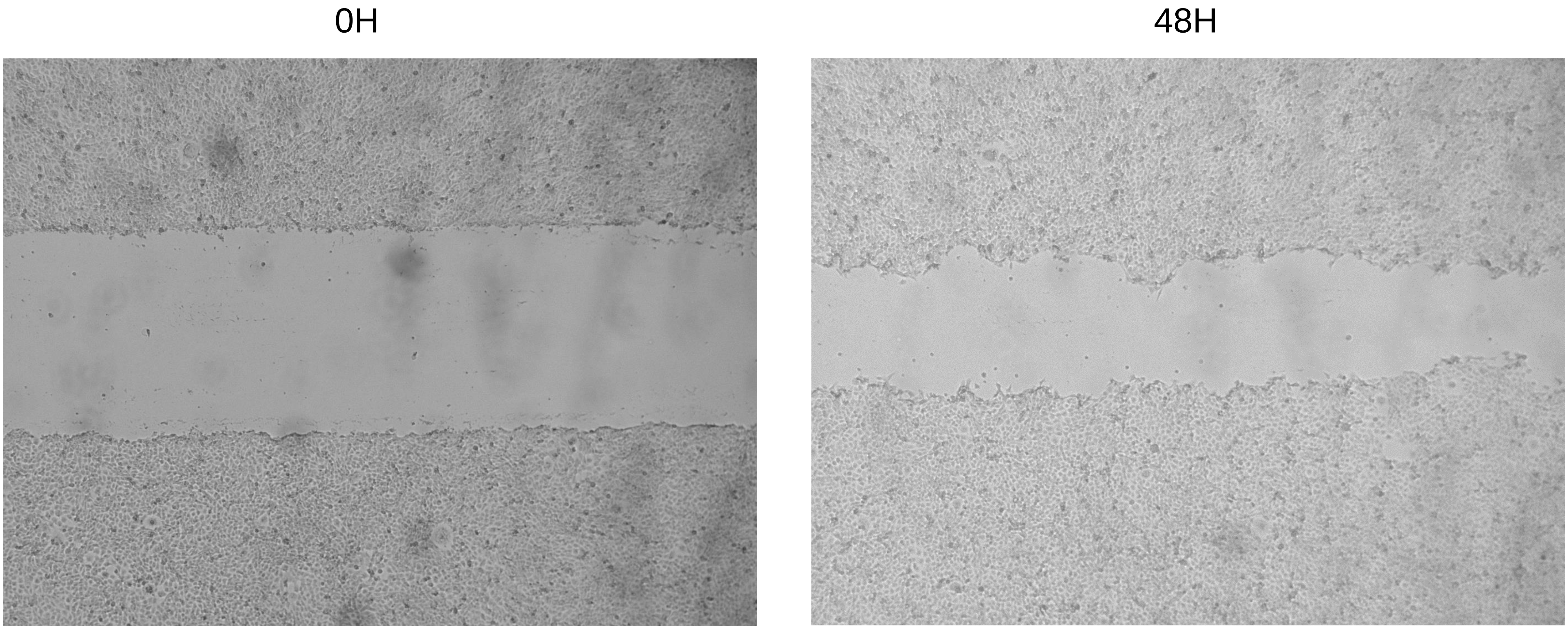

Figure s11 CAL-27-MCD

0H

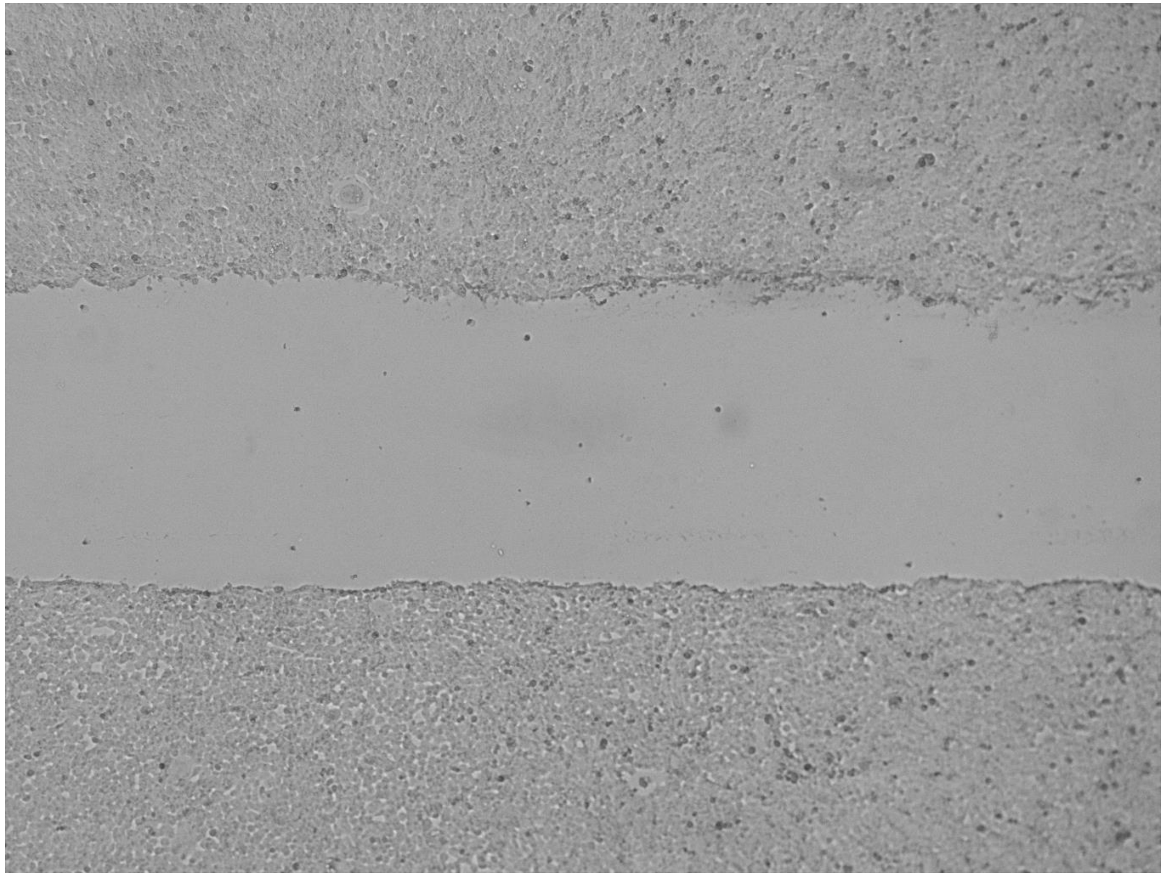

48H

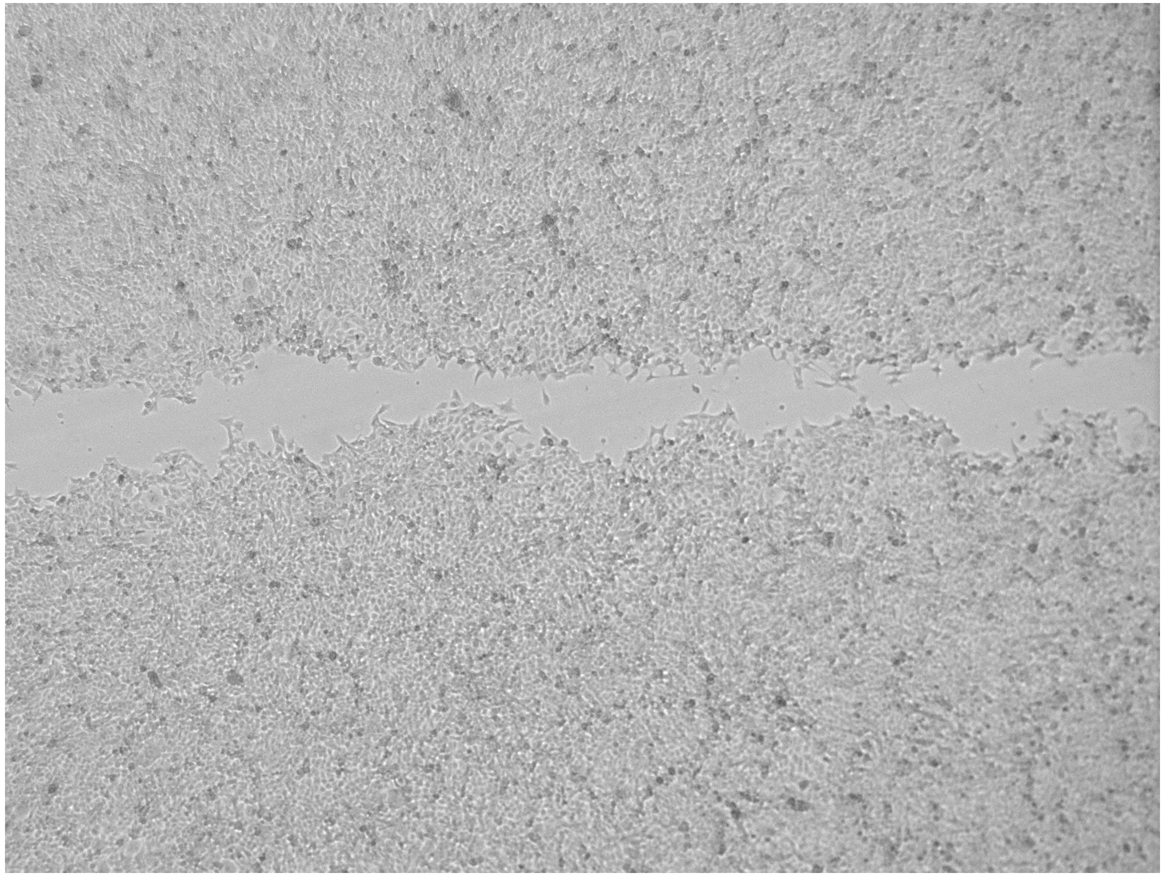

Figure s11 CAL-27-CD

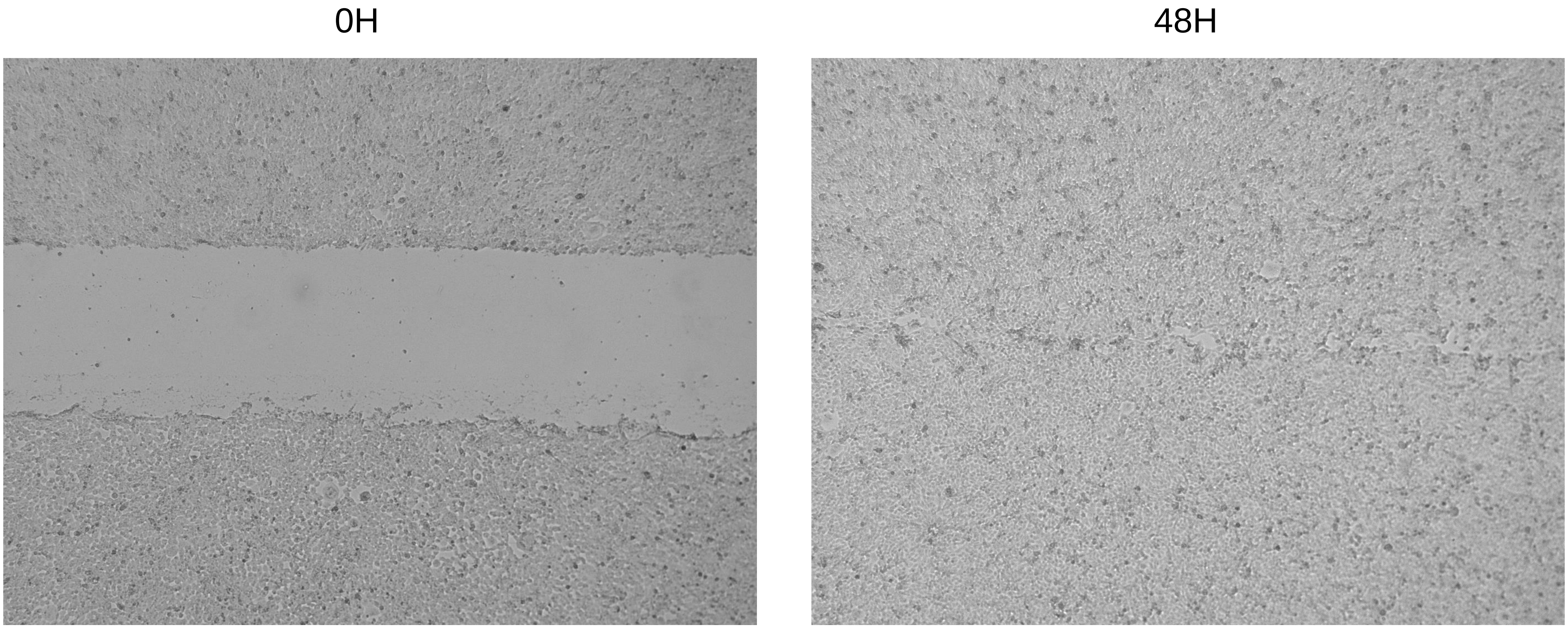

Figure s11 CAL-27-Cage-ctrl

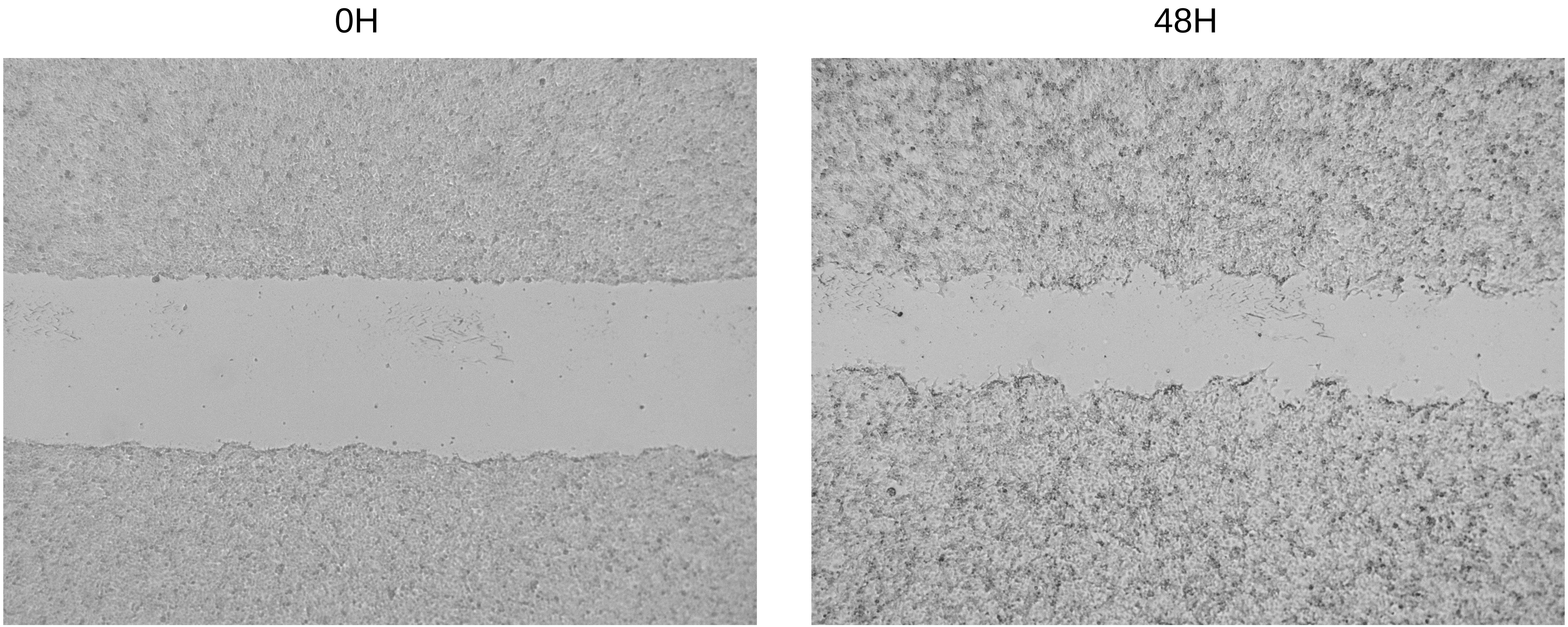

Figure s11 CAL-27-N-cage

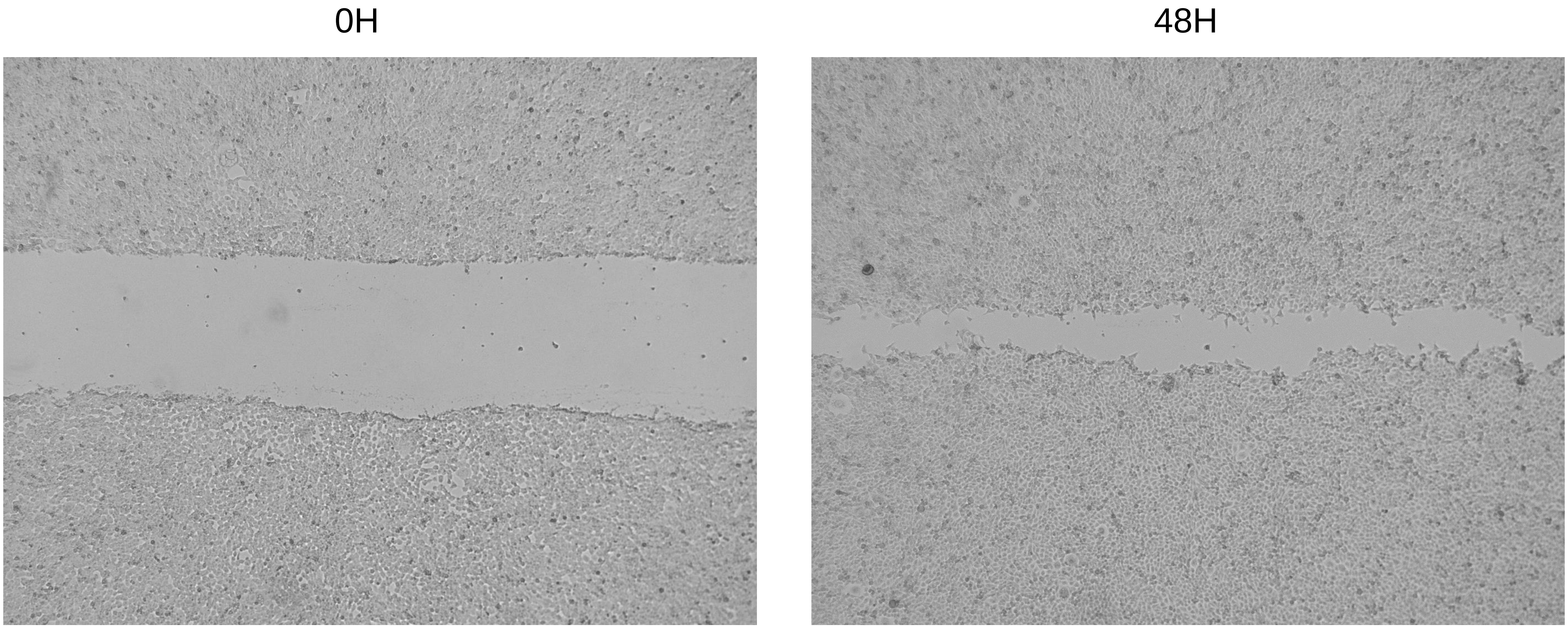

Figure s11 HSC-6-Ctrl

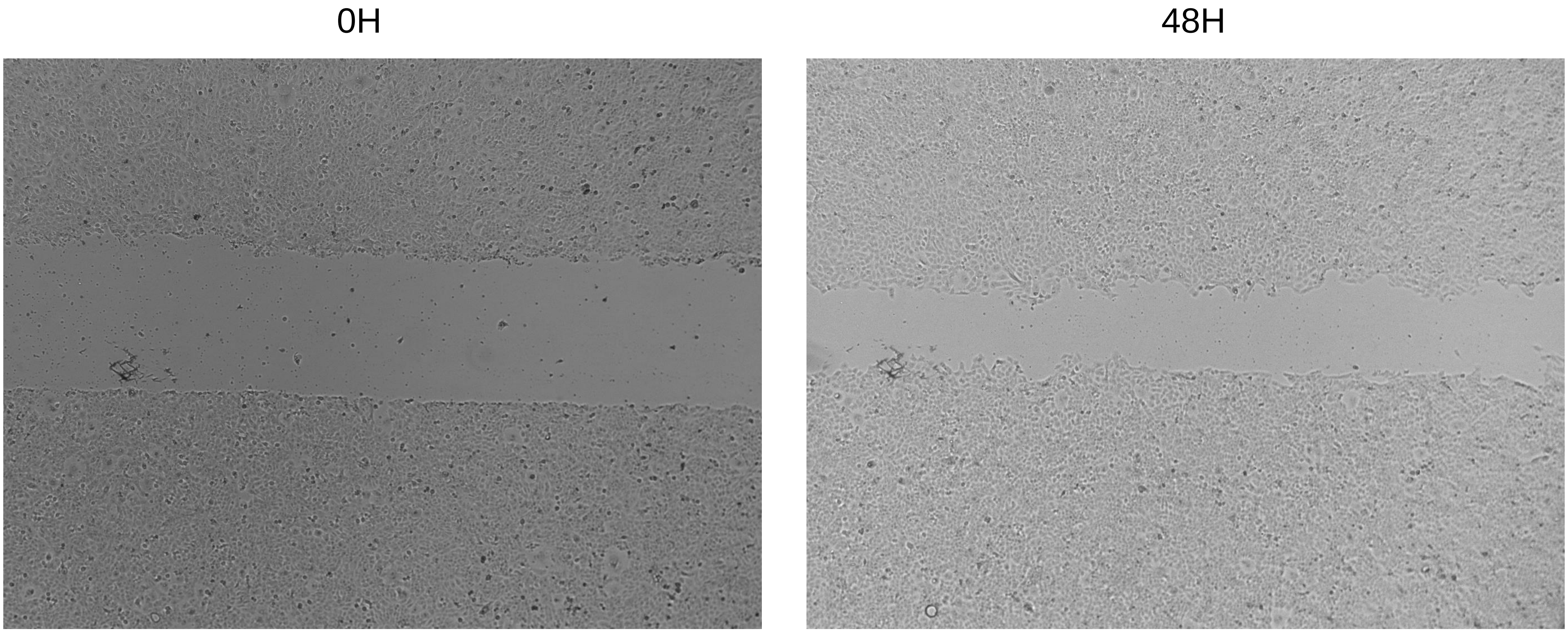

Figure s11 HSC-6-wt

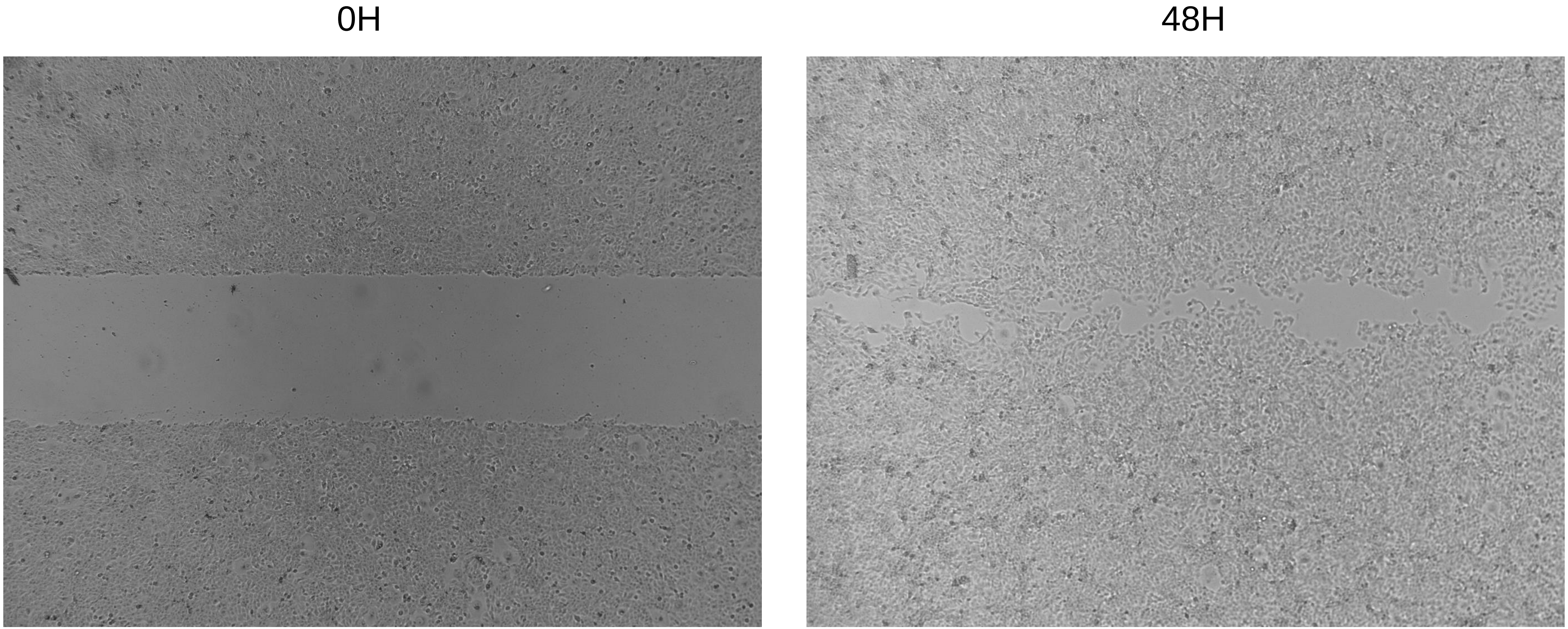

Figure s11 HSC-6-mut

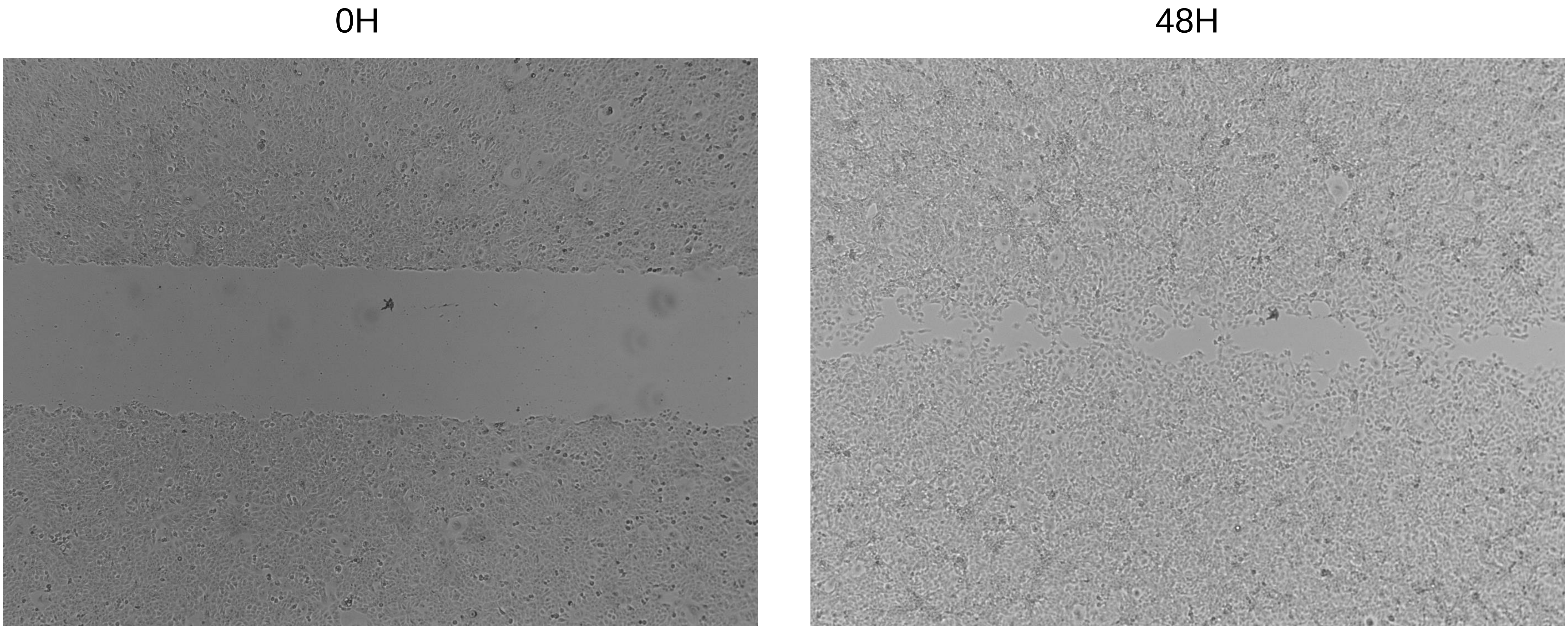

Figure s11 HSC-6-C

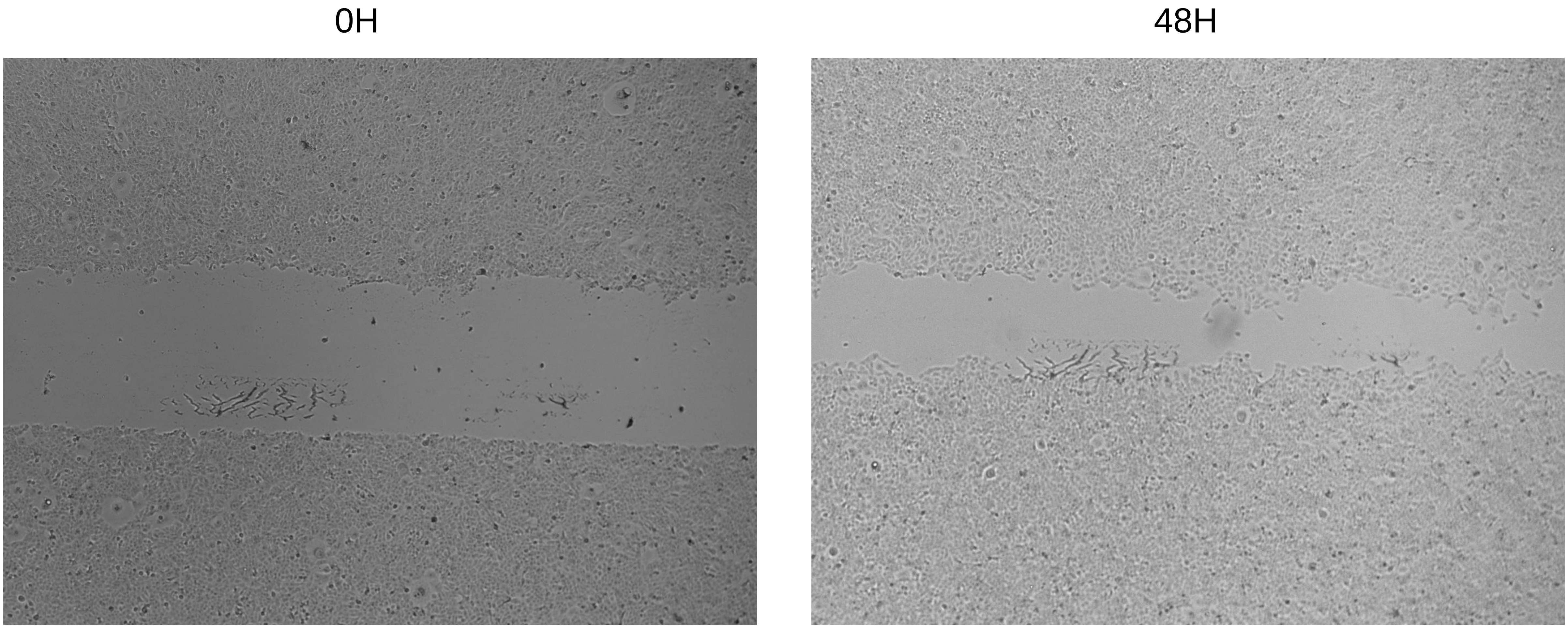

Figure s11 HSC-6-MCD

0H

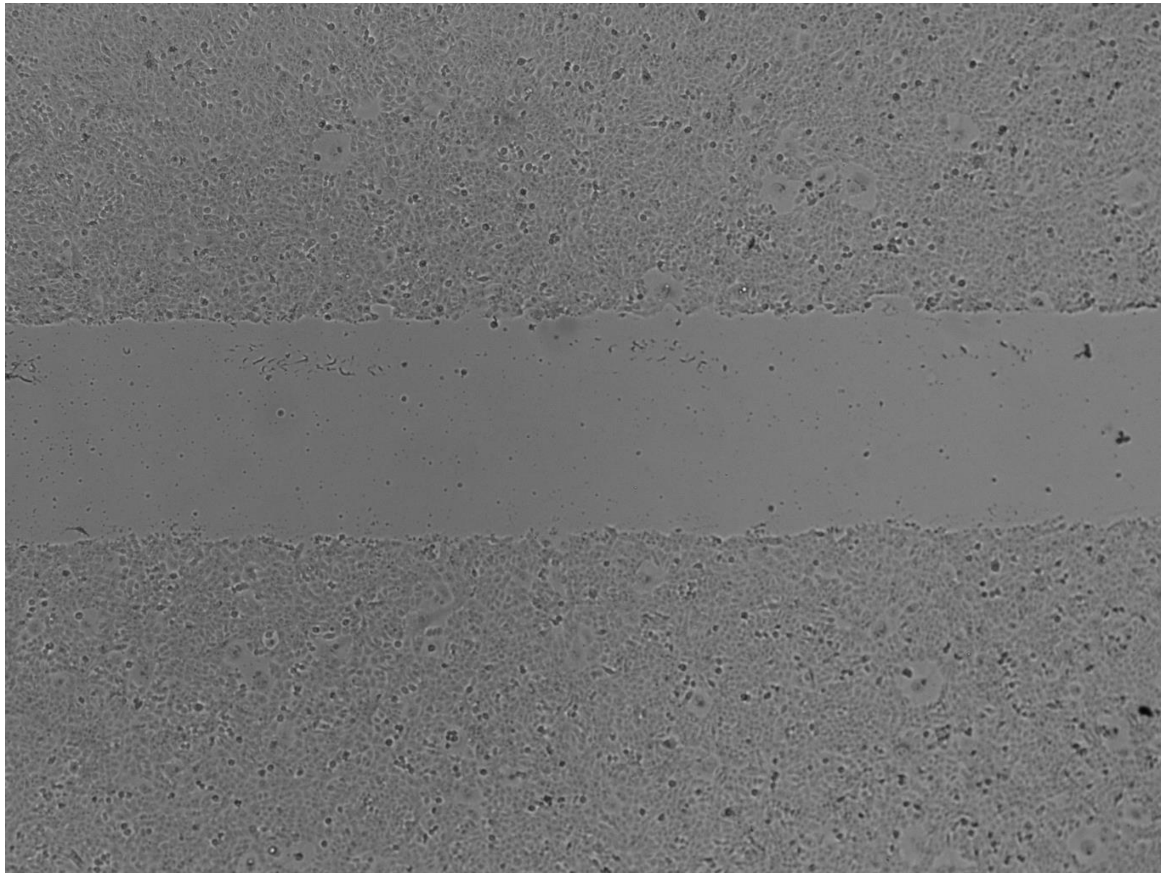

48H

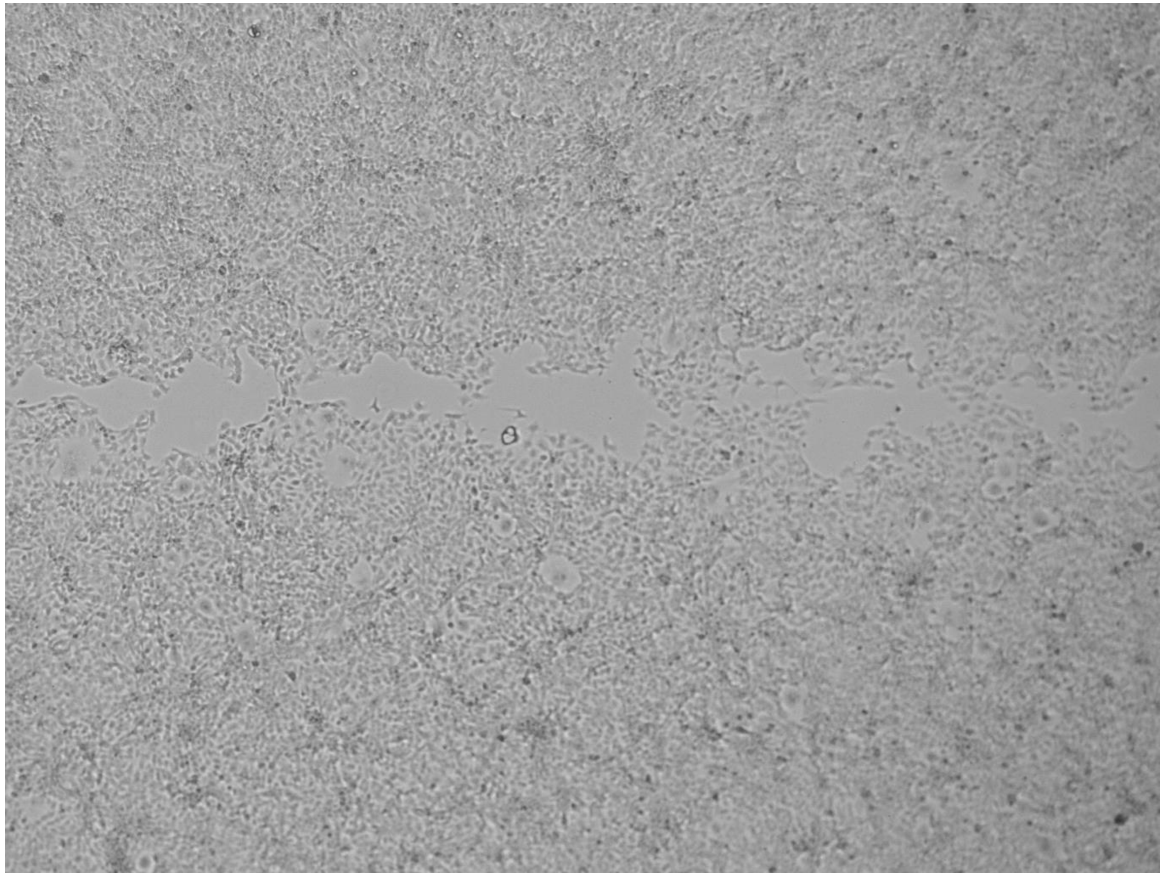

Figure s11 HSC-6-CD

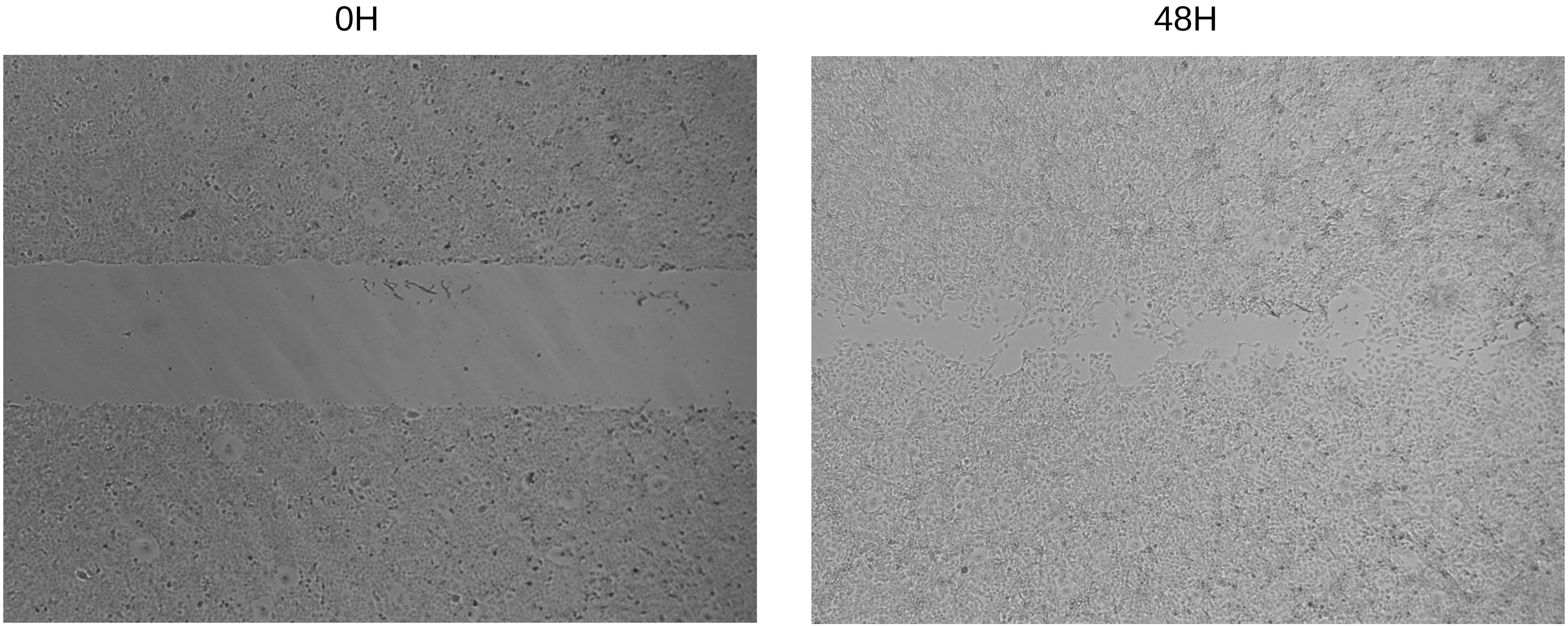

Figure s11 CAL-27-Cage-ctrl

0H 48H

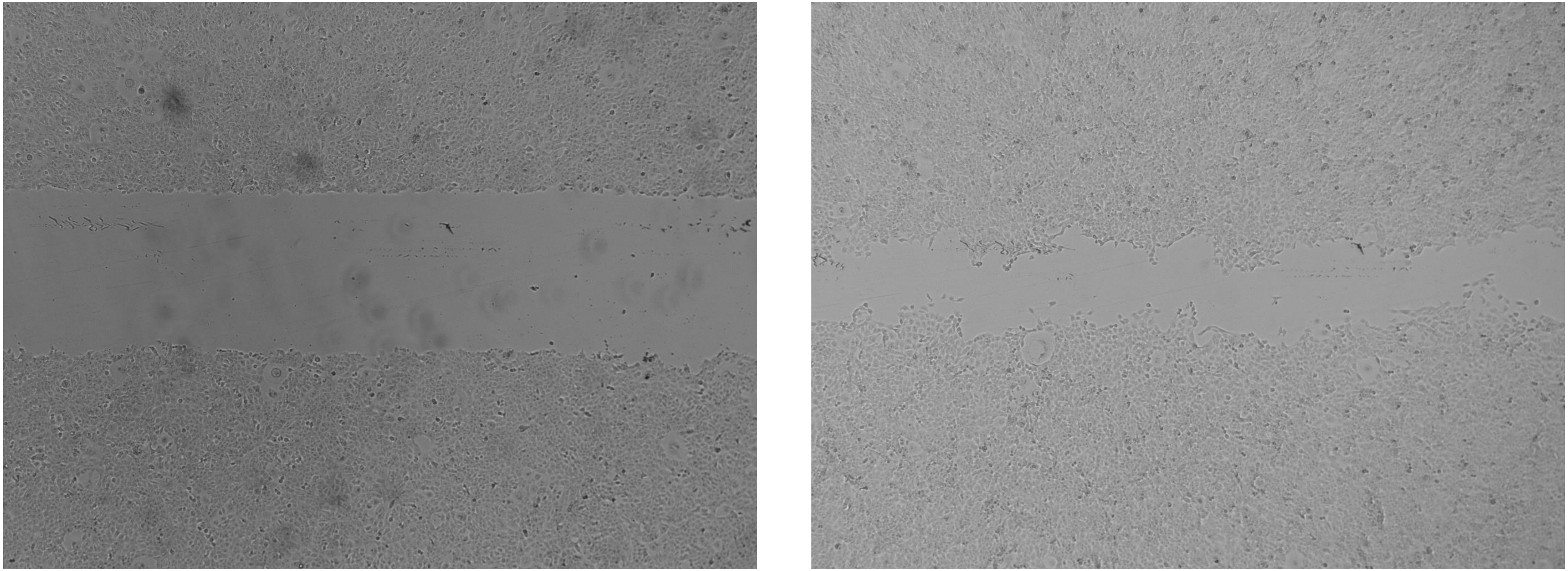

Figure s11 CAL-27-N-cage

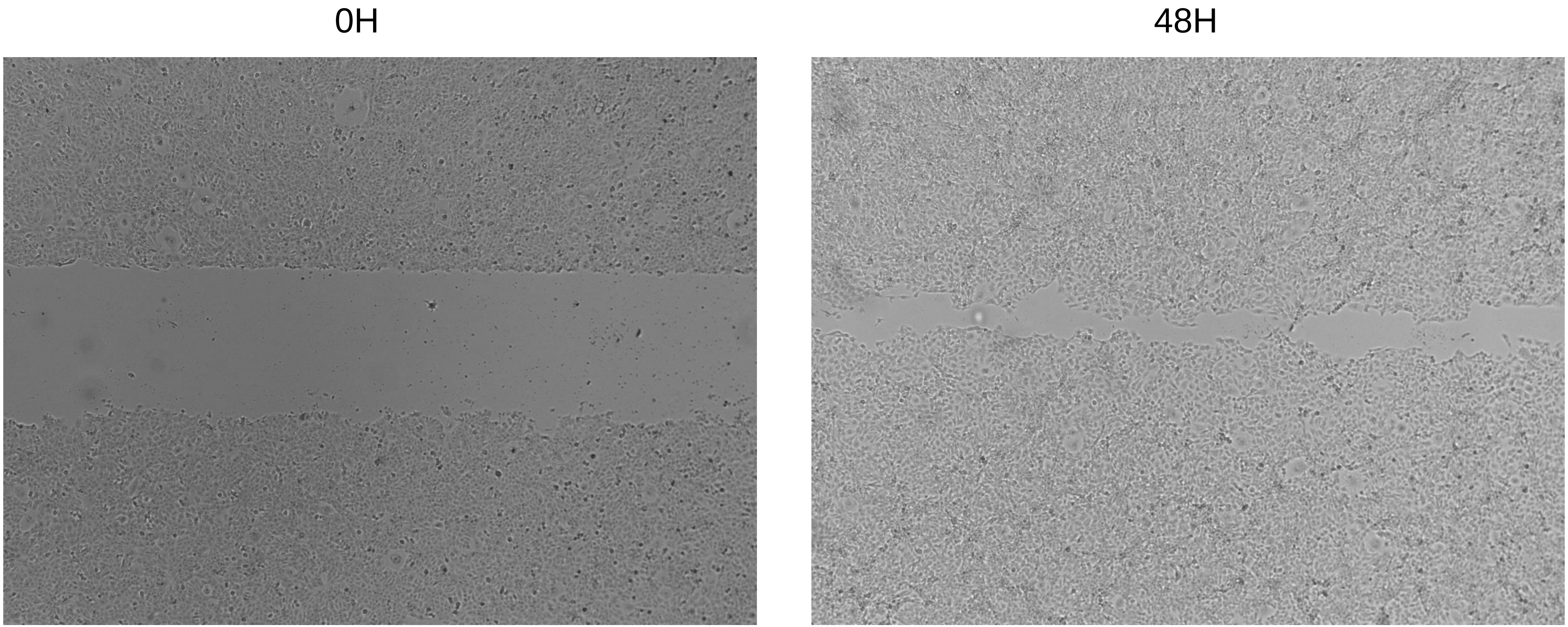

Supplement: Supplementary file 2 — Supporting Information [file ADVS-12-2501149-s003.pdf]
