# Supplementary material for: Neoadjuvant Chemotherapy With Cisplatin Up‐Regulates GSDMD to Enhance Oral Squamous Cell Carcinoma Metastasis Through MMP14‐Mediated EMT Activation
Source: Adv Sci (Weinh). 2025 Apr 3;12(25):2501149. doi: 10.1002/advs.202501149 (PMC12224931; doi:10.1002/advs.202501149)
Supplement: Supplementary file 3 — Supporting Information [file ADVS-12-2501149-s002.pdf]

ADVANCED SCIENCE
Open Access

Supporting Information

for *Adv. Sci.*, DOI 10.1002/advs.202501149

Neoadjuvant Chemotherapy With Cisplatin Up-Regulates GSDMD to Enhance Oral Squamous Cell Carcinoma Metastasis Through MMP14-Mediated EMT Activation

Zixian Huang, Qiming Jiang, Qianyu Zhang, Nan Lu, Xi Rui, Rui Chen, Yan Wang, Yuepeng Wang, Xiaoding Xu and Zhiquan Huang**

Neoadjuvant Chemotherapy with Cisplatin Up-regulates GSDMD to Enhance Oral Squamous Cell Carcinoma Metastasis through MMP14-Mediated EMT Activation

Supplementary Figures & Table

Figure S1

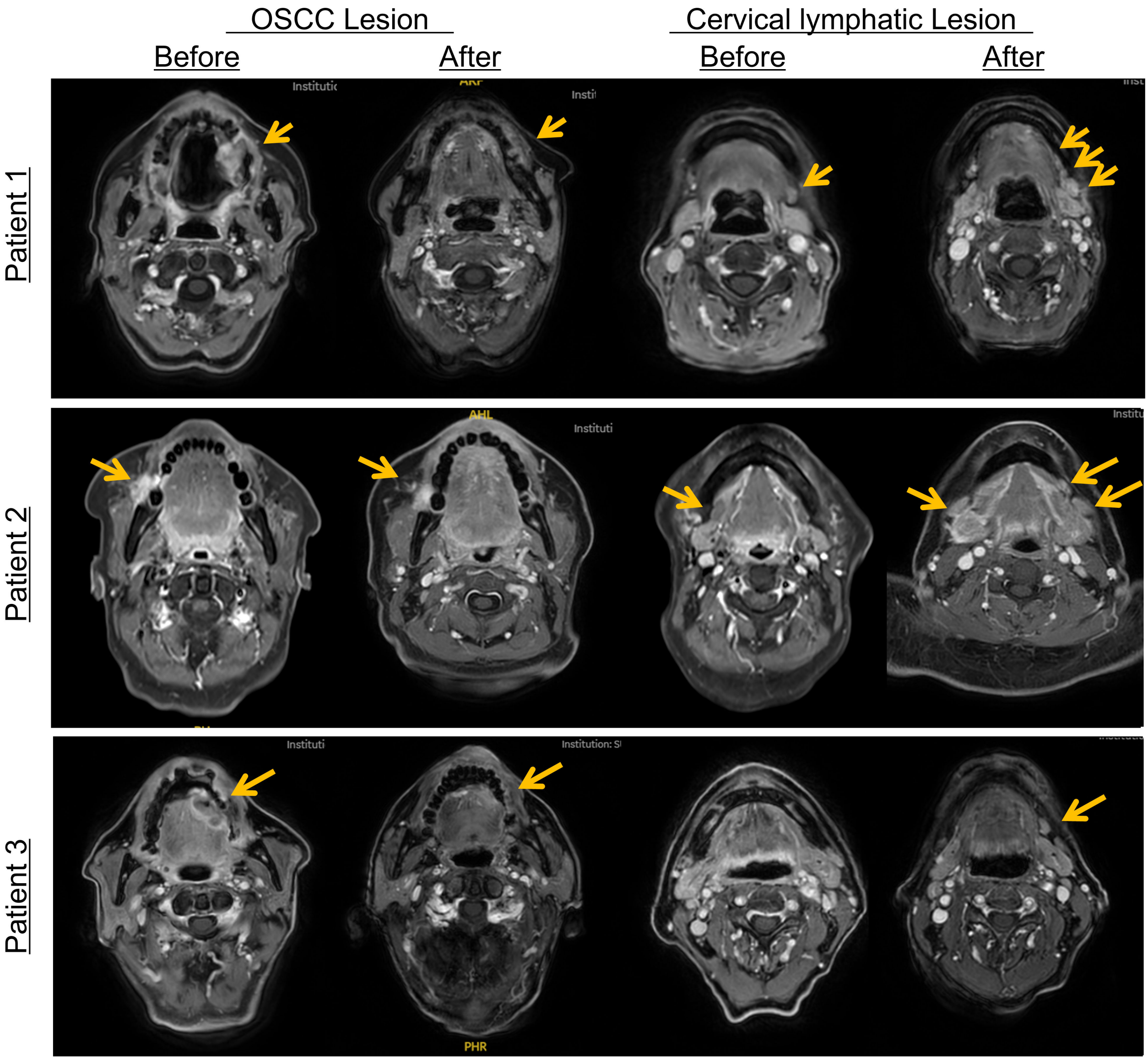

Figure S1. Lesion changes before and after neoadjuvant therapy in three oral cancer patients were sequenced.

2 cases revealed extensive new ipsilateral lymph node metastases on MRI, 1 case presented new contralateral lymph node metastases

Figure S2

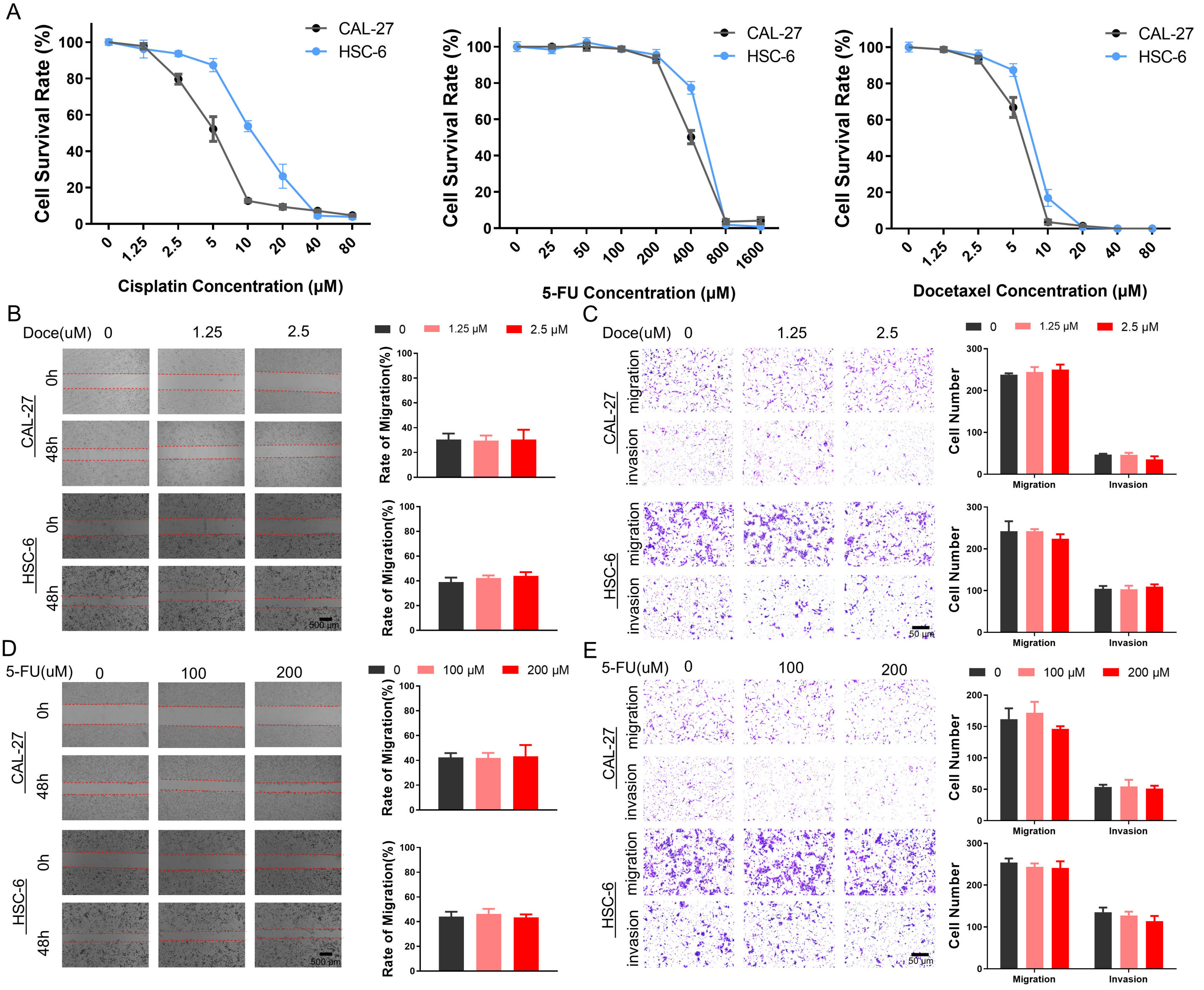

Figure S2. Chemotherapeutic sensitivity of oral squamous cell carcinoma (OSCC) cells and the effects of docetaxel and 5-FU on the migration and invasion abilities of OSCC cells.
A. Sensitivity of OSCC cells to cisplatin, docetaxel, and 5-fluorouracil chemotherapy.
B. Scratch healing assay demonstrated that migration ability of CAL-27 and HSC-6 cells was not significantly enhanced after docetaxel treatment.
C. Transwell assay showed that migration and invasion abilities of CAL-27 and HSC-6 cells were not significantly enhanced after docetaxel treatment.
D. Scratch assay demonstrated that migration ability of CAL-27 and HSC-6 cells was not significantly enhanced after 5-FU treatment.
E. Transwell assay showed that migration and invasion abilities of CAL-27 and HSC-6 cells were not significantly enhanced after 5-FU treatment.

Figure S3

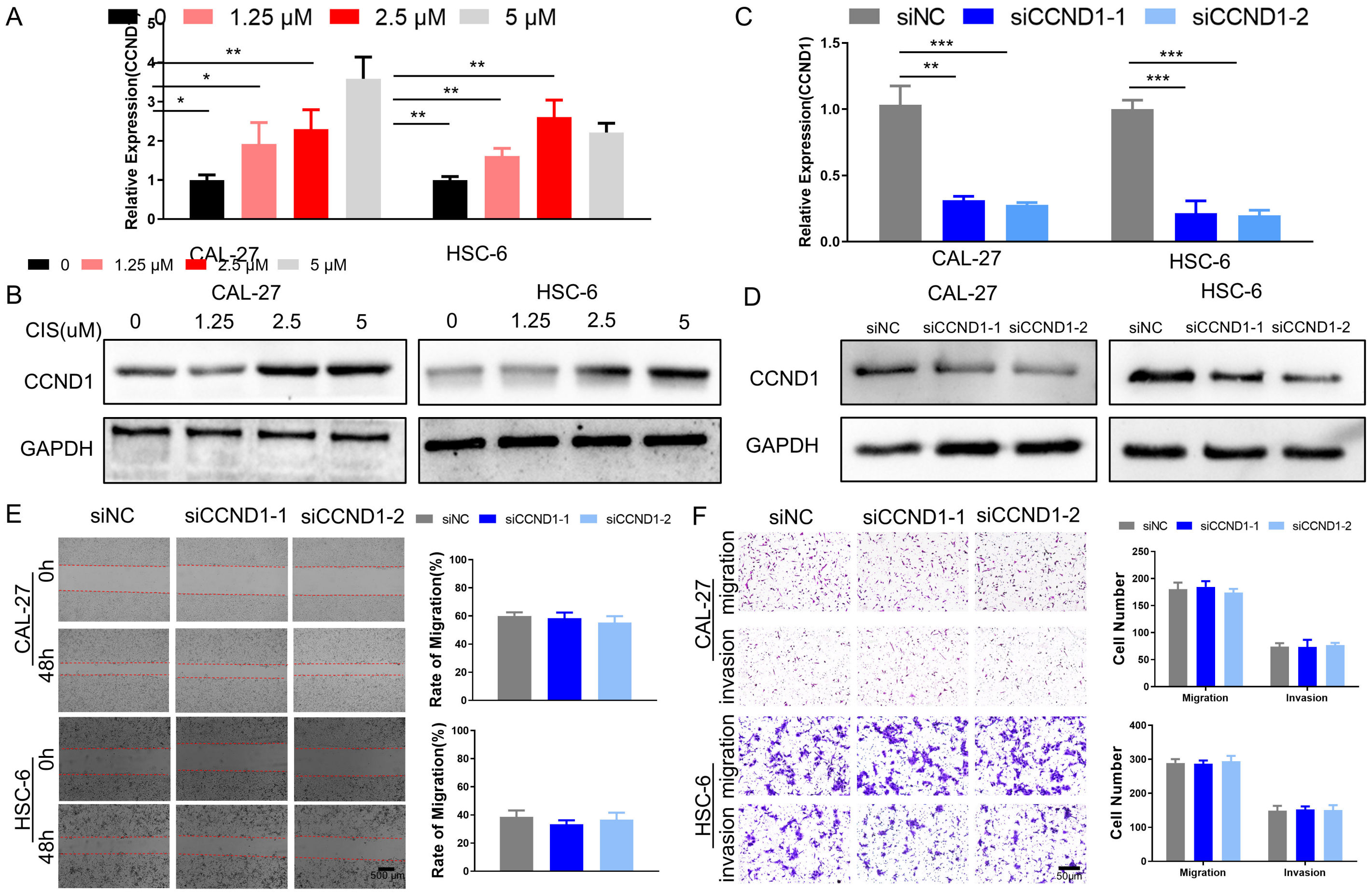

Figure S3. Knockdown of CCND1 fails to decrease the migration and invasion abilities of OSCC cells.

A. Upregulation of CCND1 transcription levels in CAL-27 and HSC-6 cells following cisplatin treatment.

B. Upregulation of CCND1 protein levels in CAL-27 and HSC-6 cells following cisplatin treatment.

C. Downregulation of CCND1 transcription levels in CAL-27 and HSC-6 cells following CCND1 knockdown.

D. Downregulation of CCND1 protein levels in CAL-27 and HSC-6 cells following CCND1 knockdown.

E. Scratch healing assay showed no significant decrease in migration ability of CAL-27 and HSC-6 cells following CCND1 knockdown.

F. Transwell assay showed no significant decrease in migration and invasion abilities of CAL-27 and HSC-6 cells following CCND1 knockdown.

Figure S4

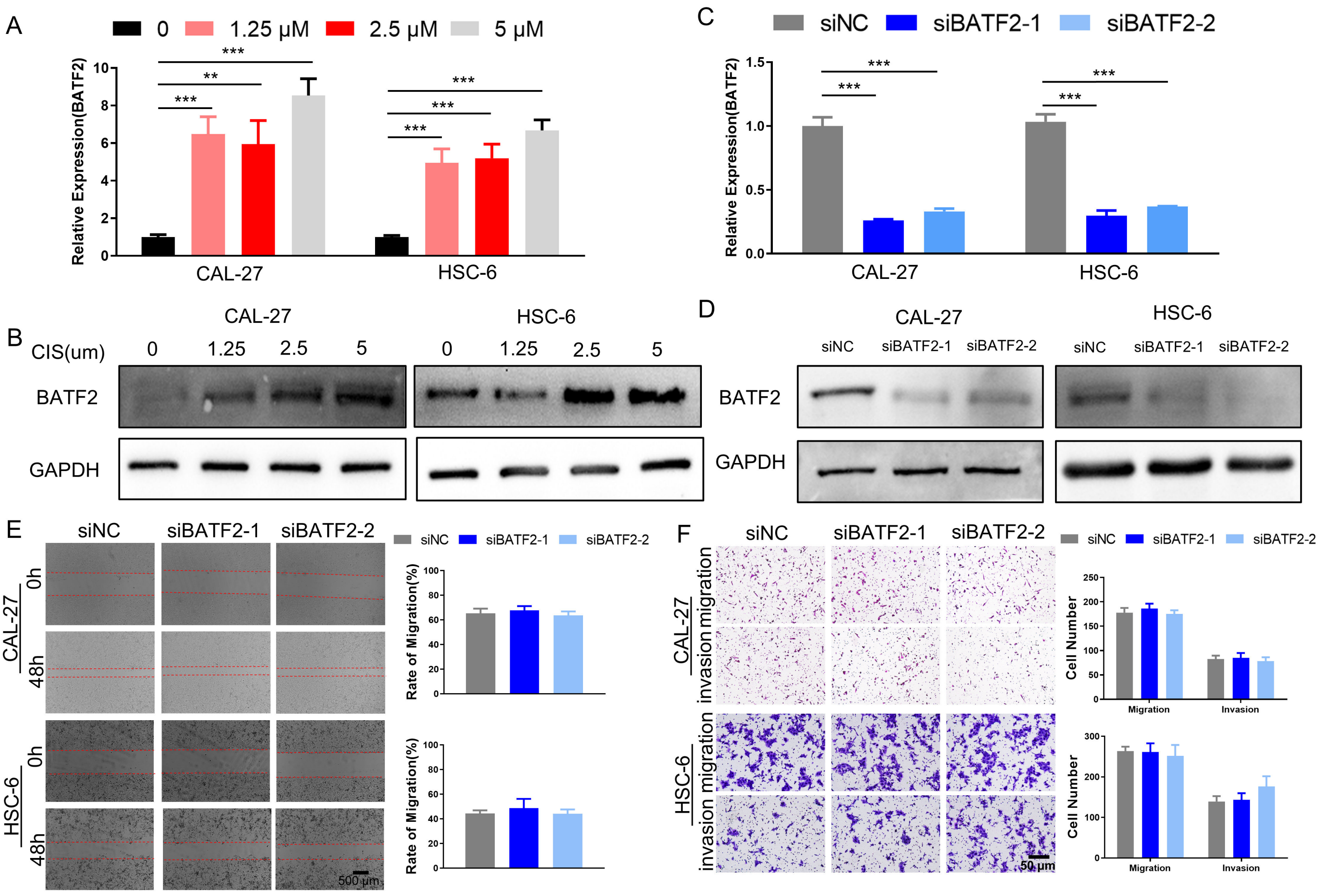

Figure S4. Knockdown of BATF2 fails to decrease the migration and invasion abilities of oral squamous cell carcinoma (OSCC) cells.

A. Upregulation of BATF2 transcription levels in CAL-27 and HSC-6 cells following cisplatin treatment.

B. Upregulation of BATF2 protein levels in CAL-27 and HSC-6 cells following cisplatin treatment.

C. Downregulation of BATF2 transcription levels in CAL-27 and HSC-6 cells following BATF2 knockdown.

D. Downregulation of BATF2 protein levels in CAL-27 and HSC-6 cells following BATF2 knockdown.

E. Scratch assay showed no significant decrease in migration ability of CAL-27 and HSC-6 cells following BATF2 knockdown.

F. Transwell assay showed no significant decrease in migration and invasion abilities of CAL-27 and HSC-6 cells following BATF2 knockdown.

Figure S5

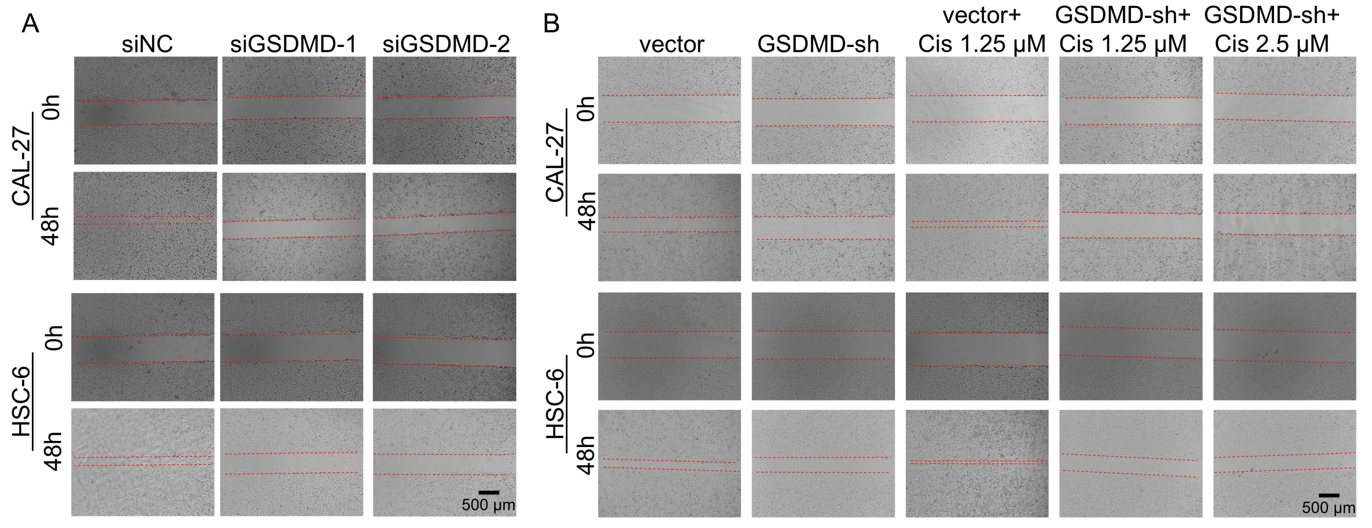

Figure S5. Knockdown of GSDMD downregulated the migration ability of OSCC cells.
A. Scratch healing assay demonstrated a significant decrease in migration ability of CAL-27 and HSC-6 cells following GSDMD knockdown.
B. Stable expression of shGSDMD resulted in a significant reduction in migration ability of CAL-27 and HSC-6 cells, and inhibited the promoting effect of cisplatin on the migration of OSCC cells.

Figure S6

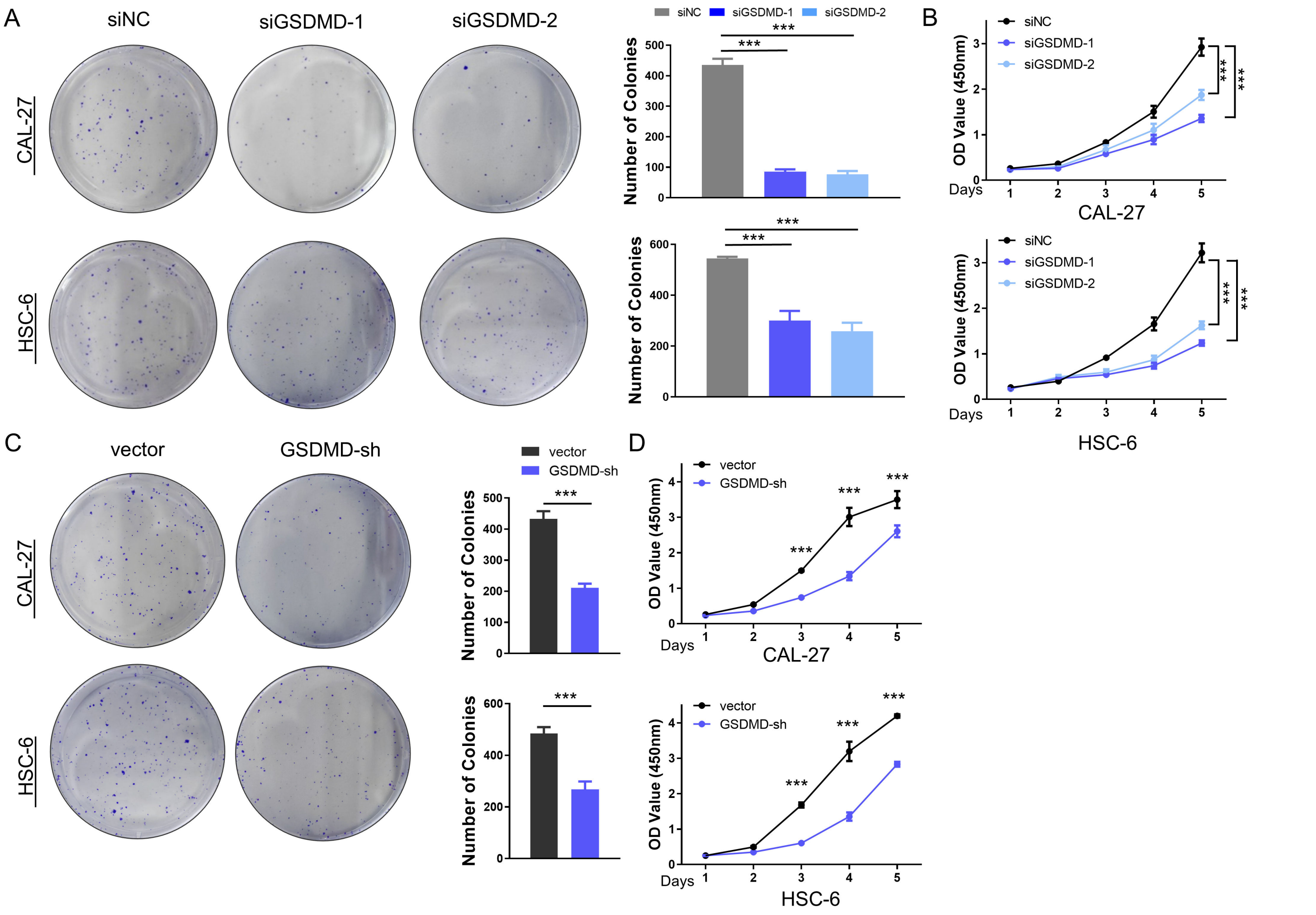

Figure S6. The effect of GSDMD knockdown on proliferation of OSCC.

A. Knockdown of GSDMD resulted in a decrease in colony formation in CAL-27 and HSC-6 cells.

B. Knockdown of GSDMD inhibited the proliferation of CAL-27 and HSC-6 cells.

C. Stable expression of shGSDMD resulted in a decrease in colony formation in CAL-27 and HSC-6 cells.

D. Stable expression of shGSDMD inhibits the proliferation of CAL-27 and HSC-6 cells.

Figure S7

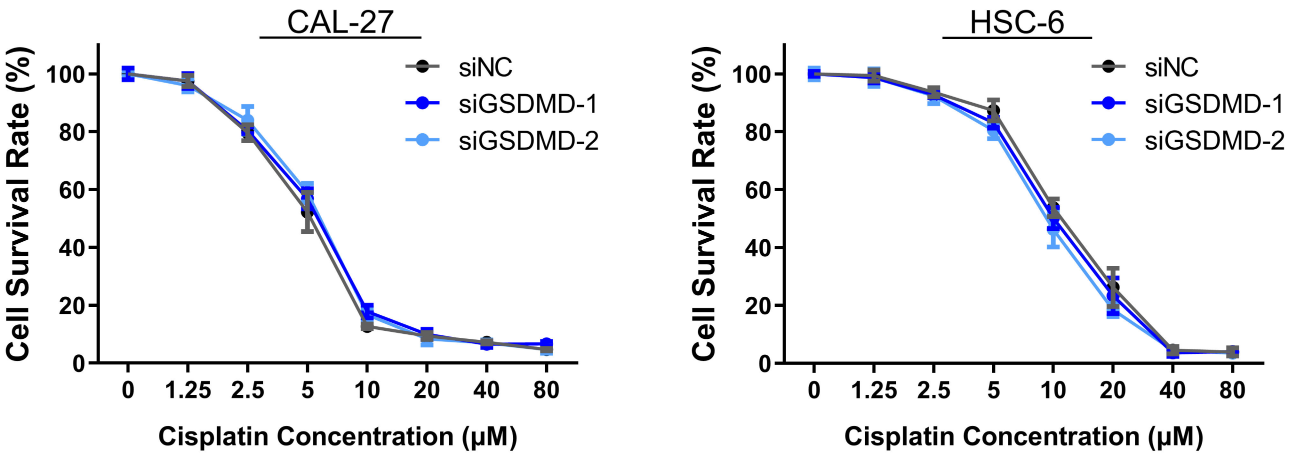

Figure S7. The effect of GSDMD knockdown on cisplatin sensitivity in OSCC. Knockdown of GSDMD did not result in significant changes in cisplatin sensitivity in CAL-27 and HSC-6 cells.

Figure S8

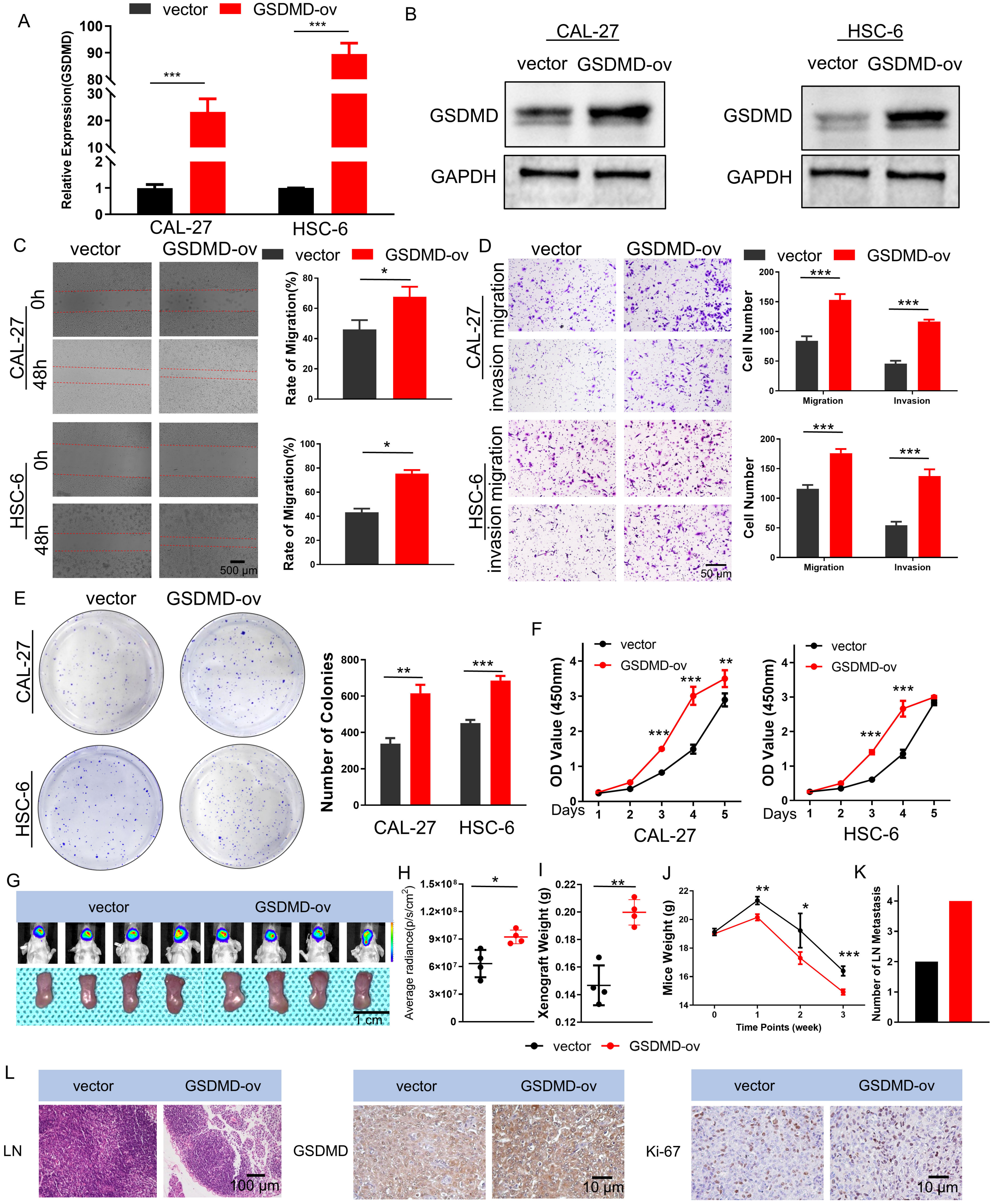

A
vector
GSDMD-ov
Relative Expression(GSDMD)
CAL-27
HSC-6
B
CAL-27
HSC-6
vector
GSDMD-ov
GSDMD
GAPDH
C
0h
48h
500 μm
Rate of Migration(%)
D
migration
invasion
50 μm
Cell Number
Migration
Invasion
E
Number of Colonies
F
OD Value (450nm)
Days
G
1 cm
H
Average radiance(p/s/cm2)
I
Xenograft Weight (g)
J
Mice Weight (g)
Time Points (week)
K
Number of LN Metastasis
L
LN
GSDMD
Ki-67
100 μm
10 μm

Figure S8. Overexpression of GSDMD Upregulated OSCC Migration, Invasion, and Lymph Node Metastasis Abilities

A. Transcription levels of GSDMD were upregulated in CAL-27 and HSC-6 cells after GSDMD overexpression.

B. Protein levels of GSDMD were upregulated in CAL-27 and HSC-6 cells after GSDMD overexpression.

C. Scratch assay showed a significant increase in migration ability of CAL-27 and HSC-6 cells after GSDMD overexpression.

D. Transwell assay demonstrated a significant increase in migration and invasion abilities of CAL-27 and HSC-6 cells after GSDMD overexpression.

E. Overexpression of GSDMD significantly increased the colony formation of CAL-27 and HSC-6 cells.

F. Overexpression of GSDMD promoted proliferation of CAL-27 and HSC-6 cells.

G. In vivo luminescense imaging and tumor image of GSDMD-ov tongue orthotopic xenograft models.

H. Higher luminescense intensity observed in tongue orthotopic xenografts in the GSDMD-ov group.

I. Mice in the GSDMD-ov group exhibited greater weight loss compared to the control group.

J. Tumor weight of tongue orthotopic xenografts was greater in the GSDMD-ov group compared to the control group.

K. Lymph node metastasis was observed in all mice in the GSDMD-ov group, while only 2 mice in the control group exhibited lymph node metastasis.

L. HE staining showed lymph node metastasis in all mice in the GSDMD-ov group, and immunohistochemistry indicated higher expression levels of GSDMD and Ki-67 in the GSDMD-ov group.

Figure S9

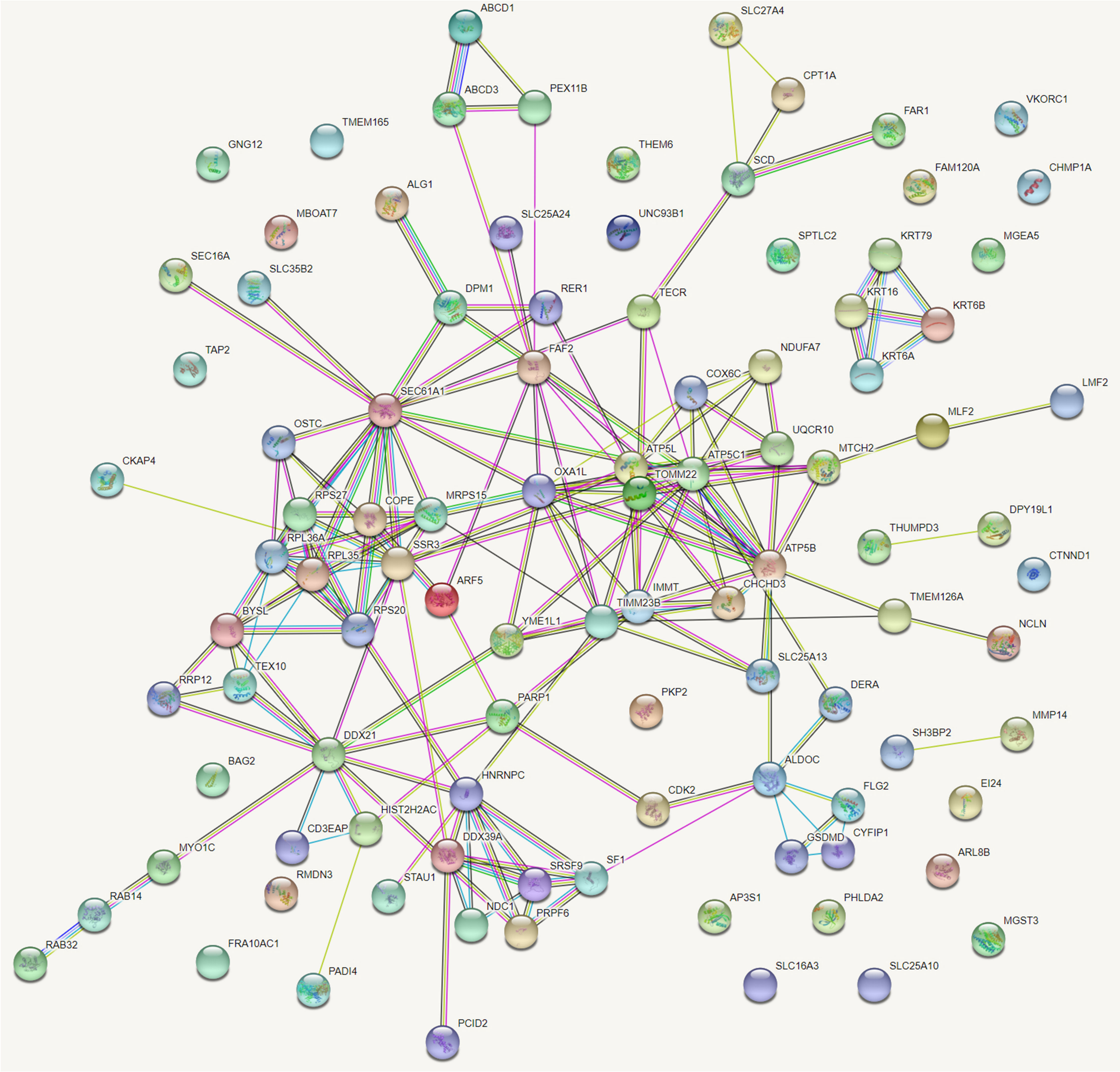

Figure S9. Network analysis of proteins that interact with GSDMD.

Figure S10

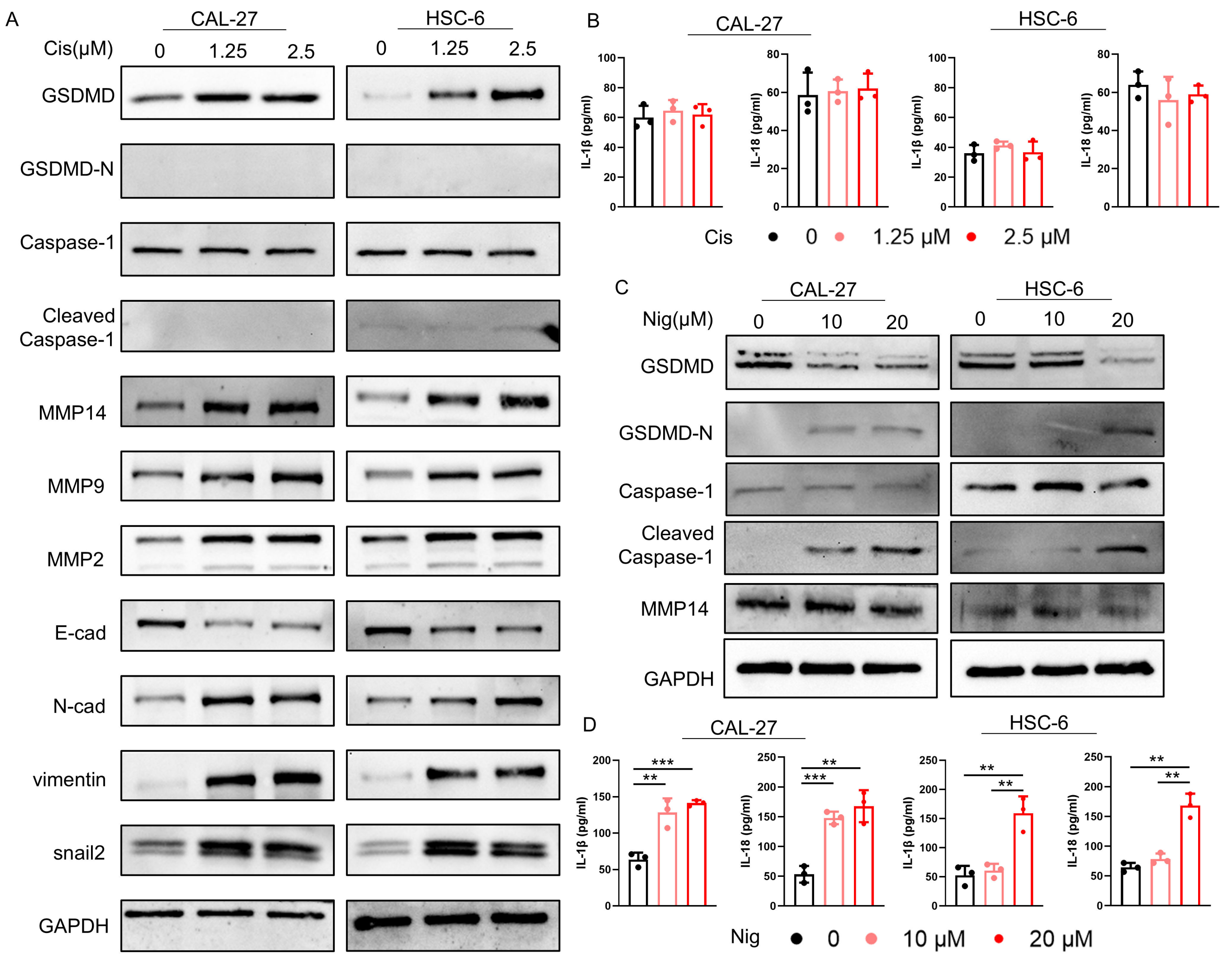

Figure S10. Protein expression and cytokine secretion in OSCC cells following treatment with cisplatin and nigericin.

A. Following stimulation with low-dose cisplatin, the protein levels of GSDMD and MMP14 in oral squamous cell carcinoma cells were synchronously upregulated, accompanied by upregulation of downstream MMP9, MMP2, N-cadherin, vimentin, and snail2, and downregulation of E-cadherin, suggesting activation of the EMT process, while GSDMD and Caspase-1 were not significantly cleaved.

B. Following stimulation with low-dose cisplatin, IL-1β and IL-18 secretion were not significantly increased.

C. Following nigericin activated pyroptosis, while Caspase-1 and GSDMD exhibited marked functional cleavage in OSCC cells, the MMP14 expression levels remained unaltered.

D. Following stimulation with nigericin, IL-1β and IL-18 secretion raised significantly, indicating activation of pyroptosis.

Figure S11

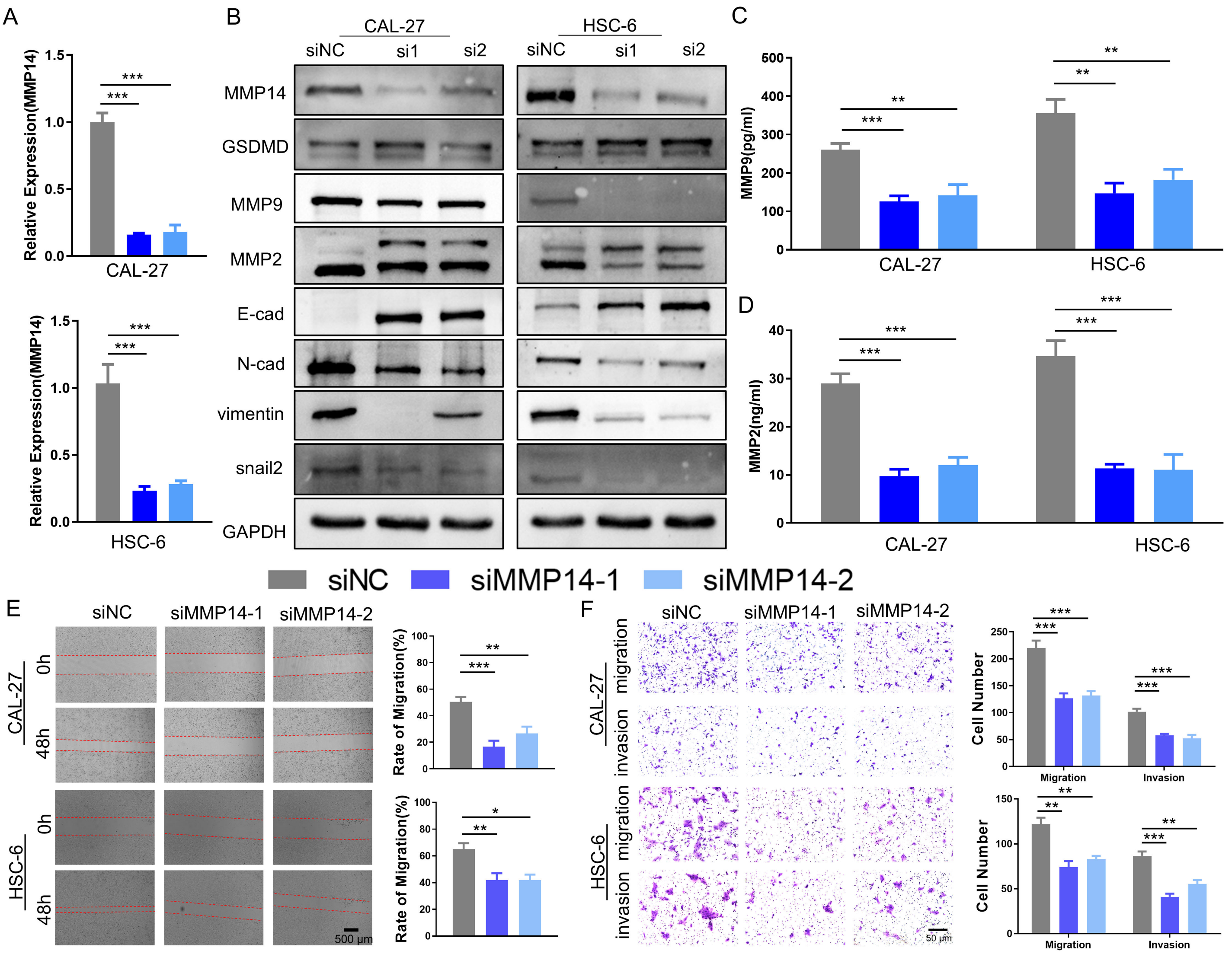

Figure S11. Knockdown of MMP14 Suppressed EMT Process and Downregulated Migration and Invasion Abilities in Oral Squamous Cell Carcinoma

A. Knockdown of MMP14 resulted in downregulation of GSDMD transcription levels in CAL-27 and HSC-6 cells.

B. Knockdown of MMP14 led to corresponding changes in the expression of EMT-related proteins in CAL-27 and HSC-6 cells, but did not alter the expression levels of GSDMD.

C. Knockdown of MMP14 led to decreased secretion levels of MMP9 in CAL-27 and HSC-6 cells.

D. Knockdown of MMP14 led to decreased secretion levels of MMP2 in CAL-27 and HSC-6 cells.

E. Scratch assay demonstrated a significant decrease in migration ability of CAL-27 and HSC-6 cells following knockdown of MMP14.

F. Transwell assay showed a significant decrease in migration and invasion abilities of CAL-27 and HSC-6 cells following knockdown of MMP14.

Figure S12

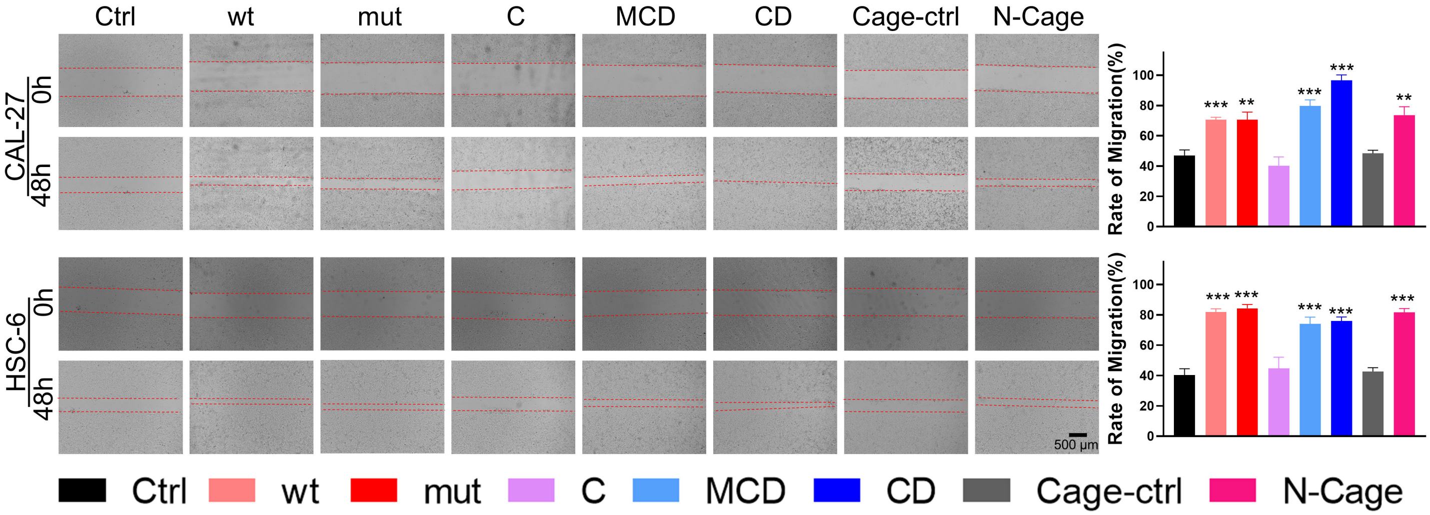

Figure S12. GSDMD Regulated Migration of Oral Squamous Cell Carcinoma Cells through Its N-terminal Domain

Scratch assay demonstrated a significant upregulation in migration ability of cells overexpressing GSDMD-wt, mut, MCD, CD, N-cage.

Figure S13

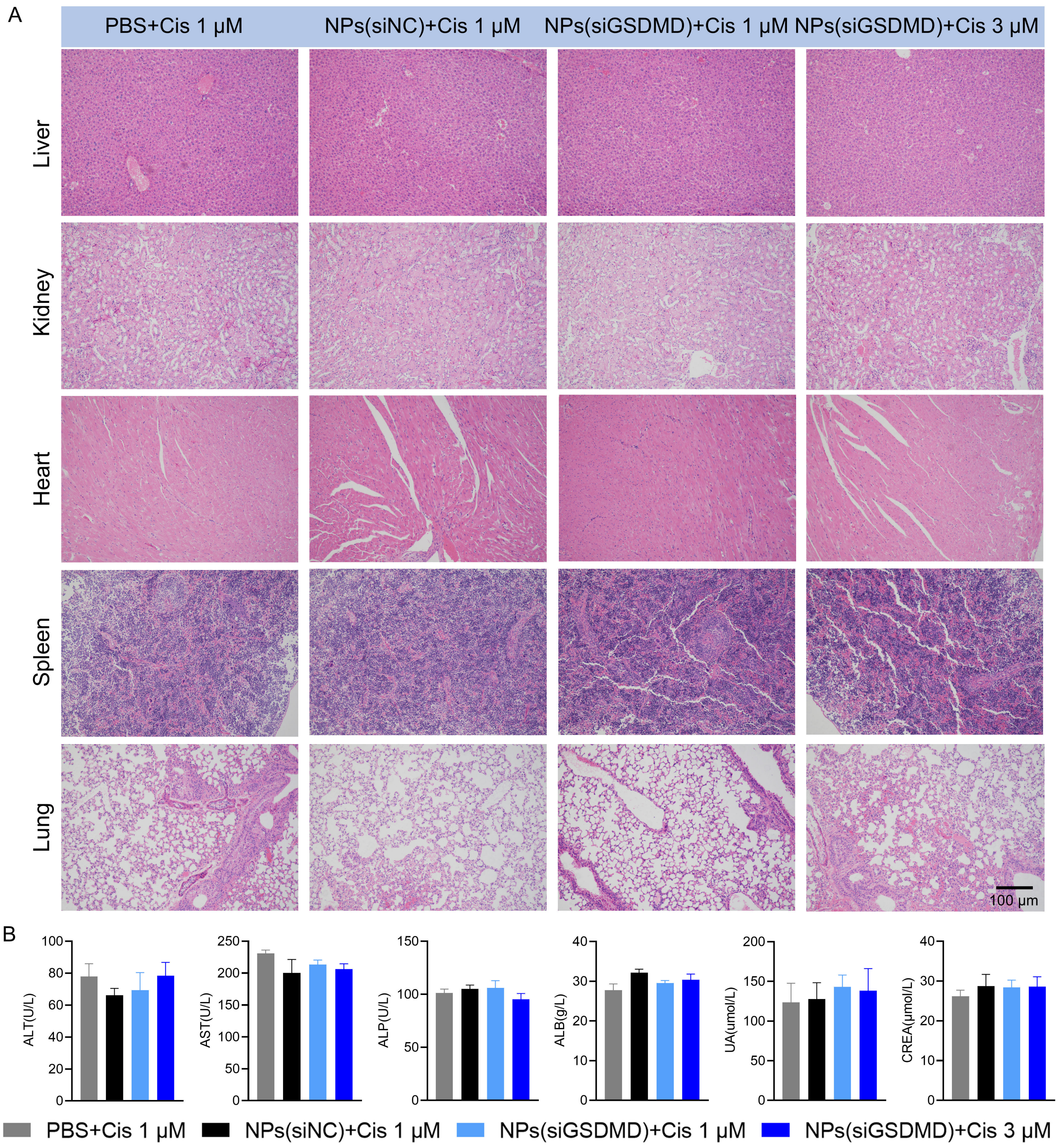

Figure S13. In Vivo Biosafety of NPs(siGSDMD)

A. No apparent pathological changes observed in major organs of mice after combination therapy of NPs(siGSDMD) with cisplatin.

B. No significant changes in liver and kidney function indicators in the blood of mice after combination therapy of NPs(siGSDMD) with cisplatin.

Table S1. Primary lesion and lymph node of 80 patients with neoadjuvant chemotherapy

Patient	Location	OSCC Lesion	Cervical lymph node
1	Right cheek	Remission	Sustain
2	Left tongue	Remission	Sustain
3	Left mandible	Remission	Sustain
4	Base of tongue	Remission	Sustain
5	Base of tongue	Remission	Sustain
6	Left tongue	Remission	Sustain
7	Left mandible	Remission	Sustain
8	Left cheek	Remission	Sustain
9	Left tongue	Remission	Sustain
10	Left mandible	Remission	Sustain
11	Left mandible	Sustain	Sustain
12	Base of tongue	Remission	Sustain
13	Left tongue	Remission	Sustain
14	Right tongue	Remission	Sustain
15	Tongue	Remission	Sustain
16	Right tongue	Remission	Sustain
17	Left tongue	Remission	Sustain
18	Right tongue	Remission	Sustain
19	Left tongue	Remission	Sustain
20	Right tongue	Sustain	Sustain
21	Left tongue	Progression	Sustain
22	Right tongue	Remission	Sustain
23	Left mandible	Remission	Sustain
24	Left tongue	Remission	Sustain
25	Left tongue	Remission	Sustain
26	Left tongue	Remission	Sustain
27	Left tongue	Remission	Remission
28	Left tongue	Remission	Remission
29	Left maxillary	Remission	Remission
30	Right tongue	Remission	Remission
31	Right tongue	Remission	Remission
32	Left tongue	Remission	Remission
33	Left tongue	Remission	Remission
34	Right mandible	Remission	Remission
35	Right tongue	Remission	Remission
36	Right tongue	Remission	Remission
37	Left maxillary	Remission	Remission
38	Left tongue	Remission	Remission
39	Right tongue	Remission	Remission
40	Right tongue	Remission	Remission

41	Right tongue	Remission	Remission
42	Left mandible	Remission	Remission
43	Left tongue	Remission	Remission
44	Right tongue	Remission	Remission
45	Right tongue	Remission	Remission
46	Left mandible	Sustain	Remission
47	Left tongue	Remission	Remission
48	Right tongue	Remission	Remission
49	Right tongue	Remission	Remission
50	Right tongue	Remission	Remission
51	Right mandible	Sustain	Remission
52	Left tongue	Remission	Remission
53	Left maxillary	Remission	Progression
54	Right cheek	Remission	Progression
55	Left tongue	Remission	Progression
56	Right cheek	Remission	Progression
57	Left mandible	Remission	Progression
58	Right mandible	Remission	Progression
59	Right tongue	Remission	Progression
60	Left mandible	Remission	Progression
61	Left cheek	Remission	Progression
62	Left tongue	Remission	Remission
63	Right mandible	Remission	Remission
64	Right tongue	Remission	Remission
65	Right tongue	Remission	Remission
66	Left tongue	Remission	Remission
67	Left tongue	Remission	Sustain
68	Right tongue	Remission	Remission
69	Left tongue	Remission	Remission
70	Right tongue	Remission	Remission
71	Left tongue	Remission	Remission
72	Left tongue	Remission	Sustain
73	Left tongue	Remission	Sustain
74	Right cheek	Remission	Remission
75	Right tongue	Remission	Sustain
76	Right tongue	Remission	Sustain
77	Right tongue	Remission	Remission
78	Right tongue	Remission	Remission
79	Left tongue	Remission	Sustain
80	Right tongue	Remission	Remission
